# Supplementary material for: A shorter splicing isoform antagonizes ZBP1 to modulate cell death and inflammatory responses
Source: EMBO J. 2024 Sep 19;43(21):12. doi: 10.1038/s44318-024-00238-7 (PMC11535224; doi:10.1038/s44318-024-00238-7)
Supplement: Supplementary file 1 — Table EV1 [file 44318_2024_238_MOESM1_ESM.docx]

Table EV1

| **Name** | **Sequence (5' - 3')** |
| --- | --- |
| zbp1-flag-fwd | AGATCGCCTGGAGAATTGGCTAGCGCCATGGACTaCaaaG  AcgATgACGACaAGGAATTCGCAGAAGCTCCTGTTGACTT |
| zbp1-flag-rev | GCAACCCCAACCCCGGATCCTCATTGCTTGCTCAGTCCTG |
| flag-zbp1L-mNG-fwd | AcgATgACGACaAGGAATTCGCAGAAGCTCCTGTTGACTT |
| flag-zbp1L-mNG-rev | atAGAACCTCCggaGCCgccTTGCTTGCTCAGTCCTGTGT |
| flag-zbp1S-mS-fwd | AcgATgACGACaAGGAATTCGCAGAAGCTCCTGTTGACTT |
| flag-zbp1S-mS-rev | acAGAACCTCCggaGCCgccatcccagaatttaataggca |
| mNG-Fwd | AAggcGGCtccGGAGGTTCTatggtgagcaagggcgagga |
| mNG-Rev | ACCCCAACCCCGGATCCTCActtgtacagctcgtccatgc |
| mS-Fwd | atggcGGCtccGGAGGTTCTgtgagcaagggcgaggcagt |
| mS-Rev | ACCCCAACCCCGGATCCttacttgtacagctcgtccatgc |
| pcw-cuo-zbp1short-mscarlet-blast-F1 | ggggacaattctcgacctcgagacaaatggcagtattcatcc |
| pcw-cuo-zbp1short-mscarlet-blast-R1 | ttaccgtaagttatgtaacggacatatcgattcgcggcca |
| pcw-cuo-zbp1short-mscarlet-blast-F2 | cgttacataacttacggtaa |
| pcw-cuo-zbp1short-mscarlet-blast-R2 | TcATcgTCtttGtAGTCCATggtgctagctctagaccaaa |
| pcw-cuo-zbp1short-mscarlet-blast-F3 | ATGGACTaCaaaGAcgATgA |
| pcw-cuo-zbp1short-mscarlet-blast-R3 | AAAGGCGCAACCCCAACCCCACGCGTttacttgtacagctcgtcca |
| pcw-cuo-zbp1short-mscarlet-blast-F4 | GGGGTTGGGGTTGCGCCTTT |
| pcw-cuo-zbp1short-mscarlet-blast-R4 | acatcgtatgggtaagccatGGTGAATTGCTGGGGAGAGA |
| pcw-cuo-zbp1short-mscarlet-blast-F5 | atggcttacccatacgatgt |
| pcw-cuo-zbp1short-mscarlet-blast-R5 | tgagacaaaggcttggccatagggccgggattctcctcca |
| pcw-cuo-zbp1short-mscarlet3-blast-F1 | CaaaGAcgATgACGACaAGGAATTCGCAGAAGCTCCTGTT |
| pcw-cuo-zbp1short-mscarlet3-blast-R1 | atAGAACCTCCggaGCCgccatcccagaatttaataggca |
| pcw-cuo-zbp1short-mscarlet3-blast-F2 | atggcGGCtccGGAGGTTCTatggatagcaccgaggcagt |
| pcw-cuo-zbp1short-mscarlet3-blast-R2 | GCAACCCCAACCCCACGCGTttaggagccaccggagccgc |
| qPCR: ZBP1 Short F | CTCCTGCAATCCCTGAGAACT |
| qPCR: ZBP1 Short R | GGCTACATGGCAAGACTATGTC |
| qPCR: ZBP1 Long F | AAGAGTCCCCTGCGATTATTTG |
| qPCR: ZBP1 Long R | TCTGGATGGCGTTTGAATTGG |
| qPCR: ZBP1 L+S F | TCCCCAGAGCCTGCAACATGGA |
| qPCR: ZBP1 L+S R | CGTCATTCCCAGAGCCTTGGCG |
| qPCR: TBP F | CCTTGTACCCTTCACCAATGAC |
| qPCR: TBP R | ACAGCCAAGATTCACGGTAGA |
