# Supplementary figures and images for: A shorter splicing isoform antagonizes ZBP1 to modulate cell death and inflammatory responses
Source: EMBO J. 2024 Sep 19;43(21):12. doi: 10.1038/s44318-024-00238-7 (PMC11535224; doi:10.1038/s44318-024-00238-7)

## Slide 1
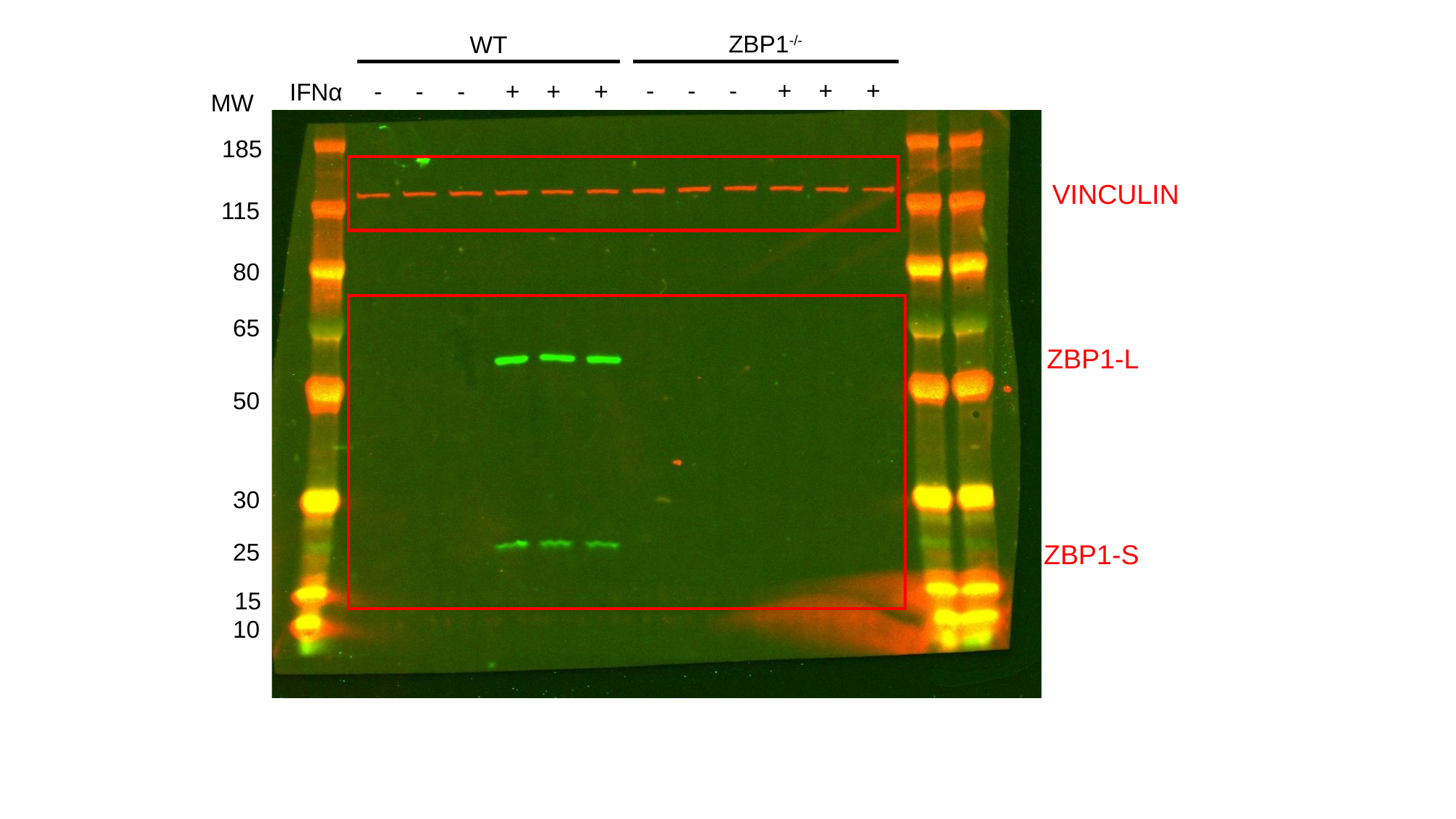

ZBP1-/-
WT
- - - + + +
- - - + + +
IFNα
MW
185
VINCULIN
115
80
65
ZBP1-L
50
30
25
ZBP1-S
15
10

Supplement: Supplementary file 3 — Source data Fig. 1 [file 44318_2024_238_MOESM3_ESM.zip › Figure 1/1E/western ZBP1 VINCULIN.pptx]

## Slide 1
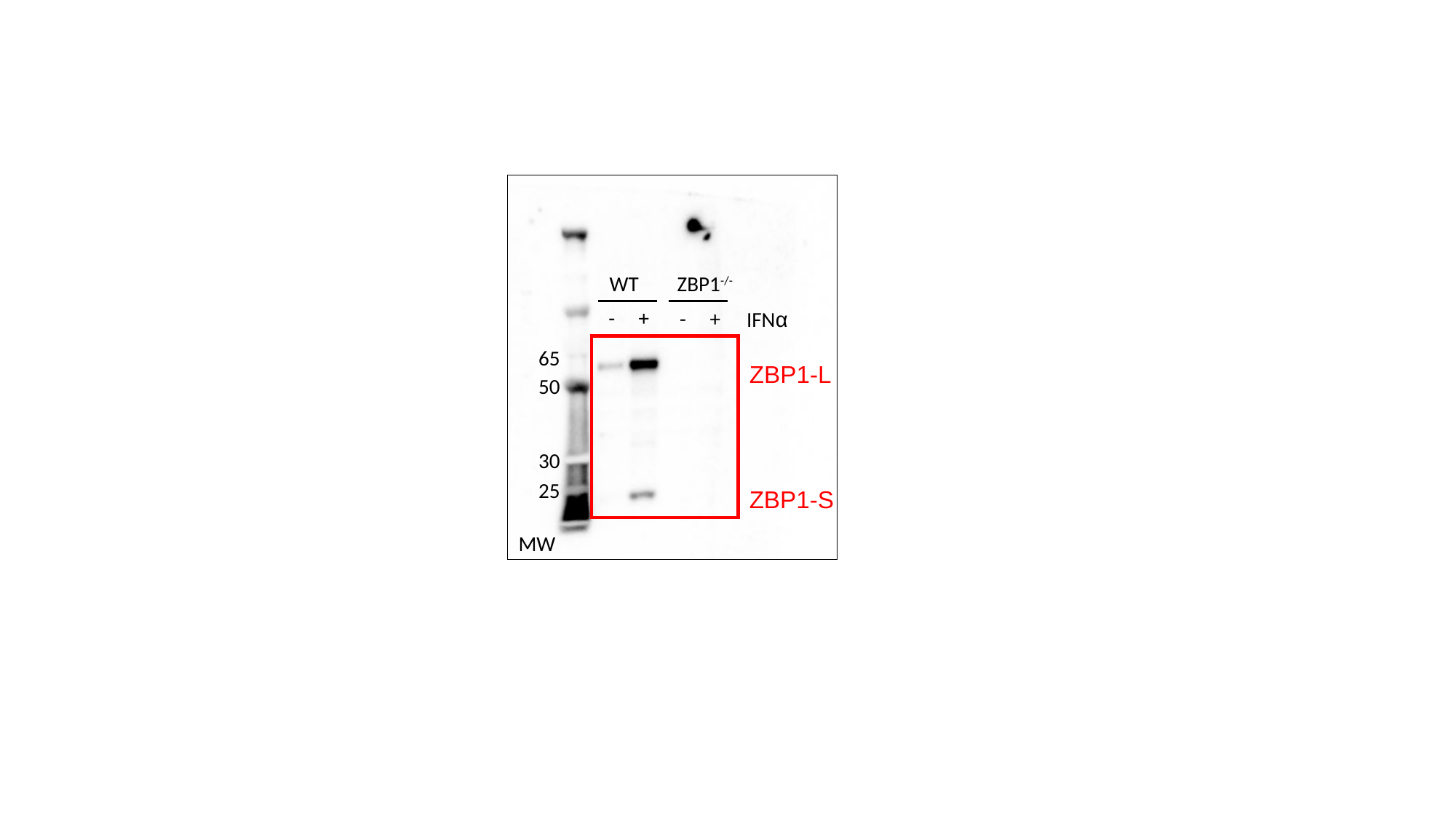

WT
ZBP1-/-
- +
- +
IFNα
65
ZBP1-L
50
30
25
ZBP1-S
MW

Supplement: Supplementary file 3 — Source data Fig. 1 [file 44318_2024_238_MOESM3_ESM.zip › Figure 1/1D/western ZBP1.pptx]

## Slide 1
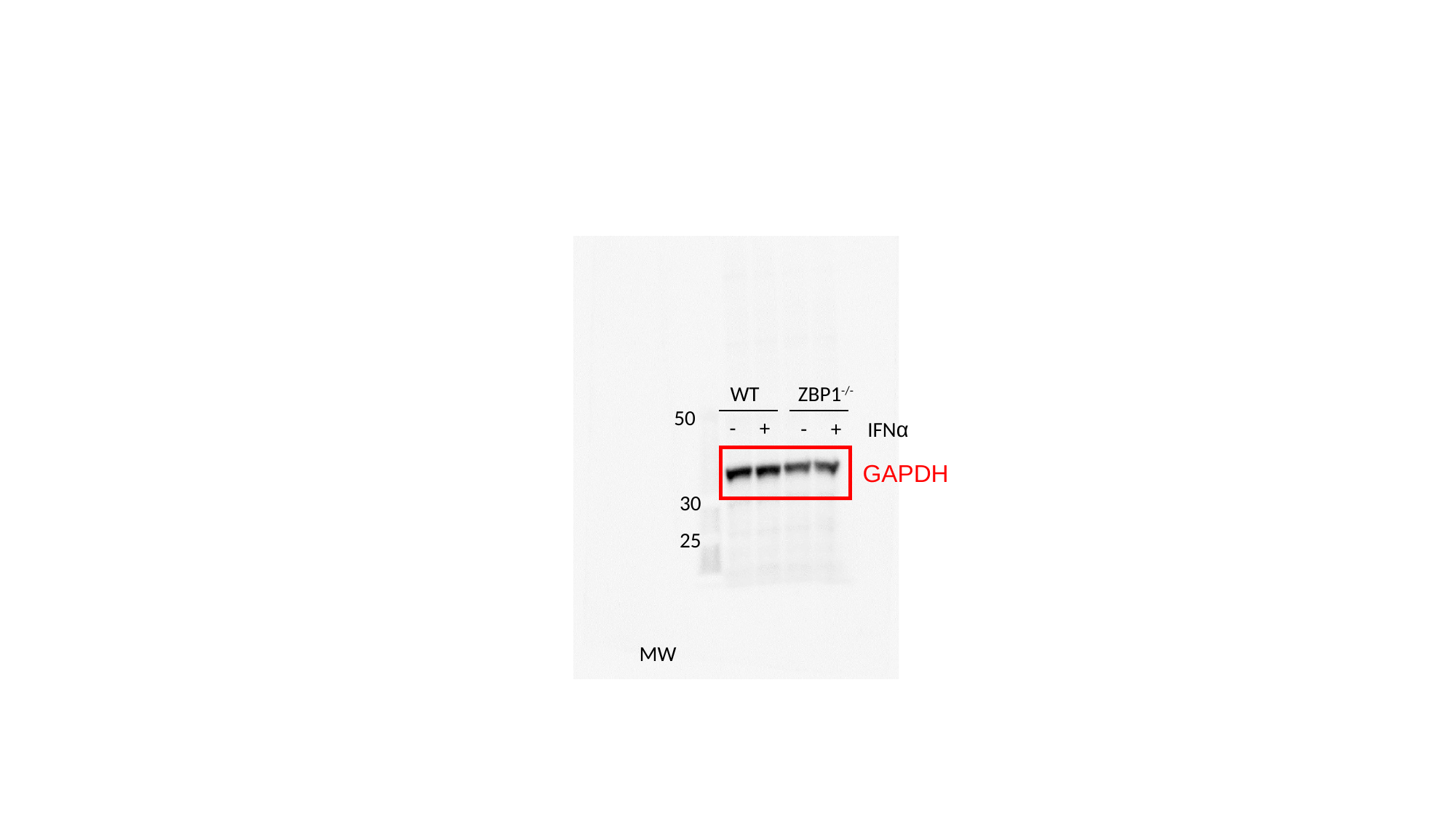

WT
ZBP1-/-
50
- +
- +
IFNα
GAPDH
30
25
MW

Supplement: Supplementary file 3 — Source data Fig. 1 [file 44318_2024_238_MOESM3_ESM.zip › Figure 1/1D/western GAPDH.pptx]

## Slide 1
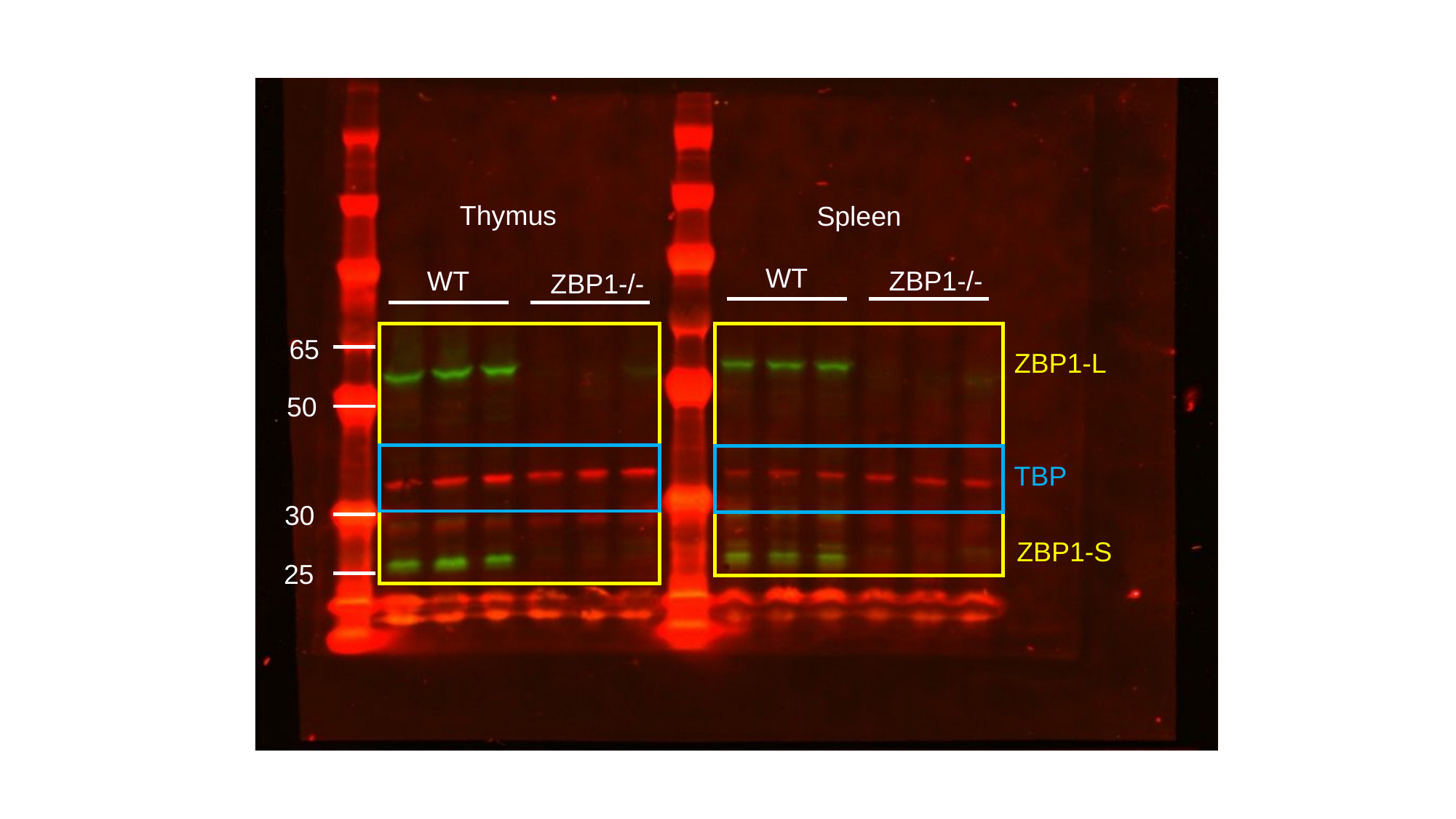

Thymus
Spleen
WT
ZBP1-/-
WT
ZBP1-/-
65
ZBP1-L
50
TBP
30
ZBP1-S
25

Supplement: Supplementary file 3 — Source data Fig. 1 [file 44318_2024_238_MOESM3_ESM.zip › Figure 1/1C/western ZBP1 TBP.pptx]

## Slide 1
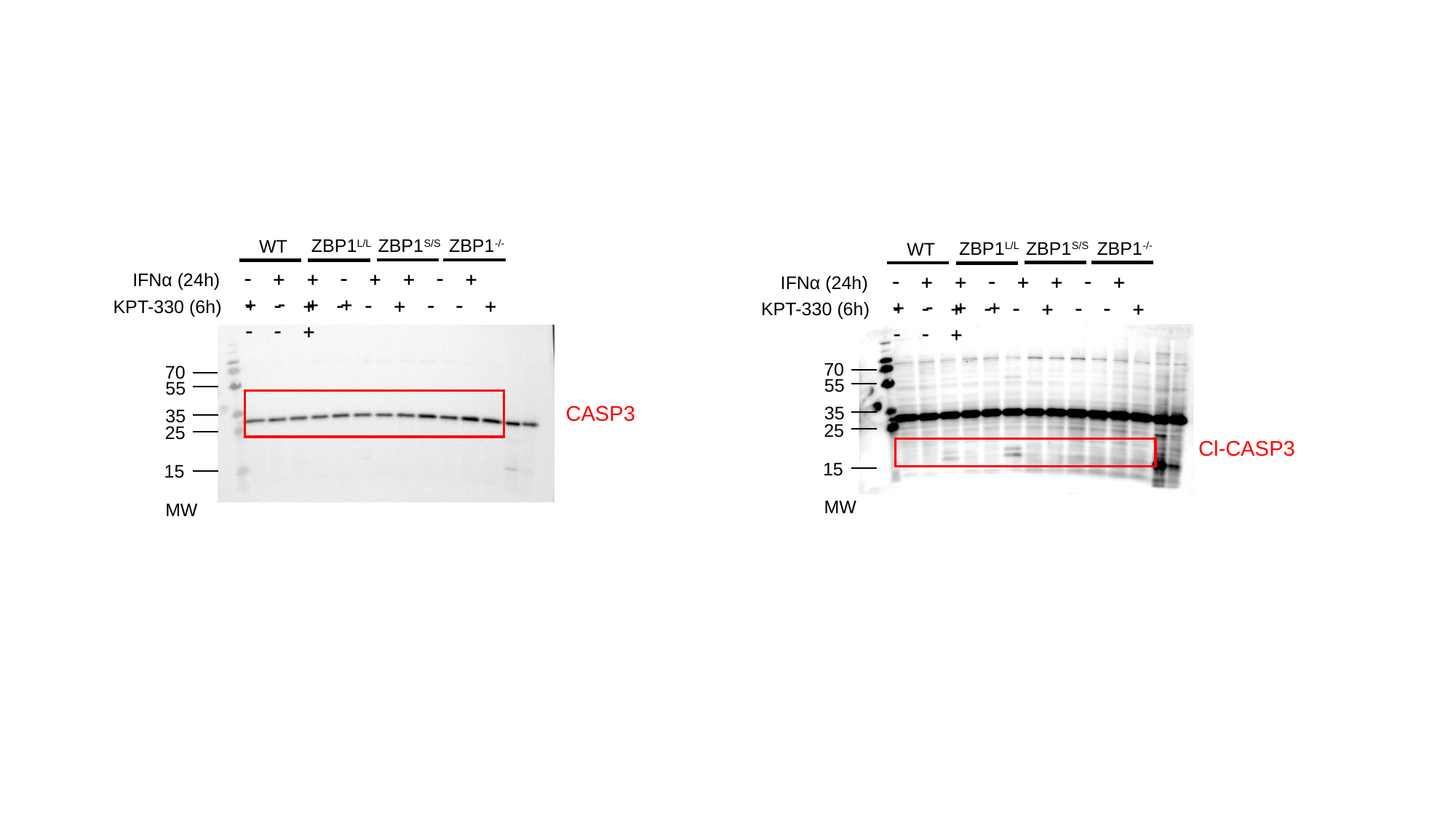

ZBP1-/-
ZBP1S/S
ZBP1L/L
WT
ZBP1-/-
ZBP1S/S
ZBP1L/L
WT
- + + - + + - + + - + +
- + + - + + - + + - + +
IFNα (24h)
IFNα (24h)
- - + - - + - - + - - +
KPT-330 (6h)
- - + - - + - - + - - +
KPT-330 (6h)
70
70
55
55
CASP3
35
35
25
25
Cl-CASP3
15
15
MW
MW

Supplement: Supplementary file 4 — Source data Fig. 2 [file 44318_2024_238_MOESM4_ESM.zip › Figure 2/2G/western CASP3 Cl-CASP3.pptx]

## Slide 1
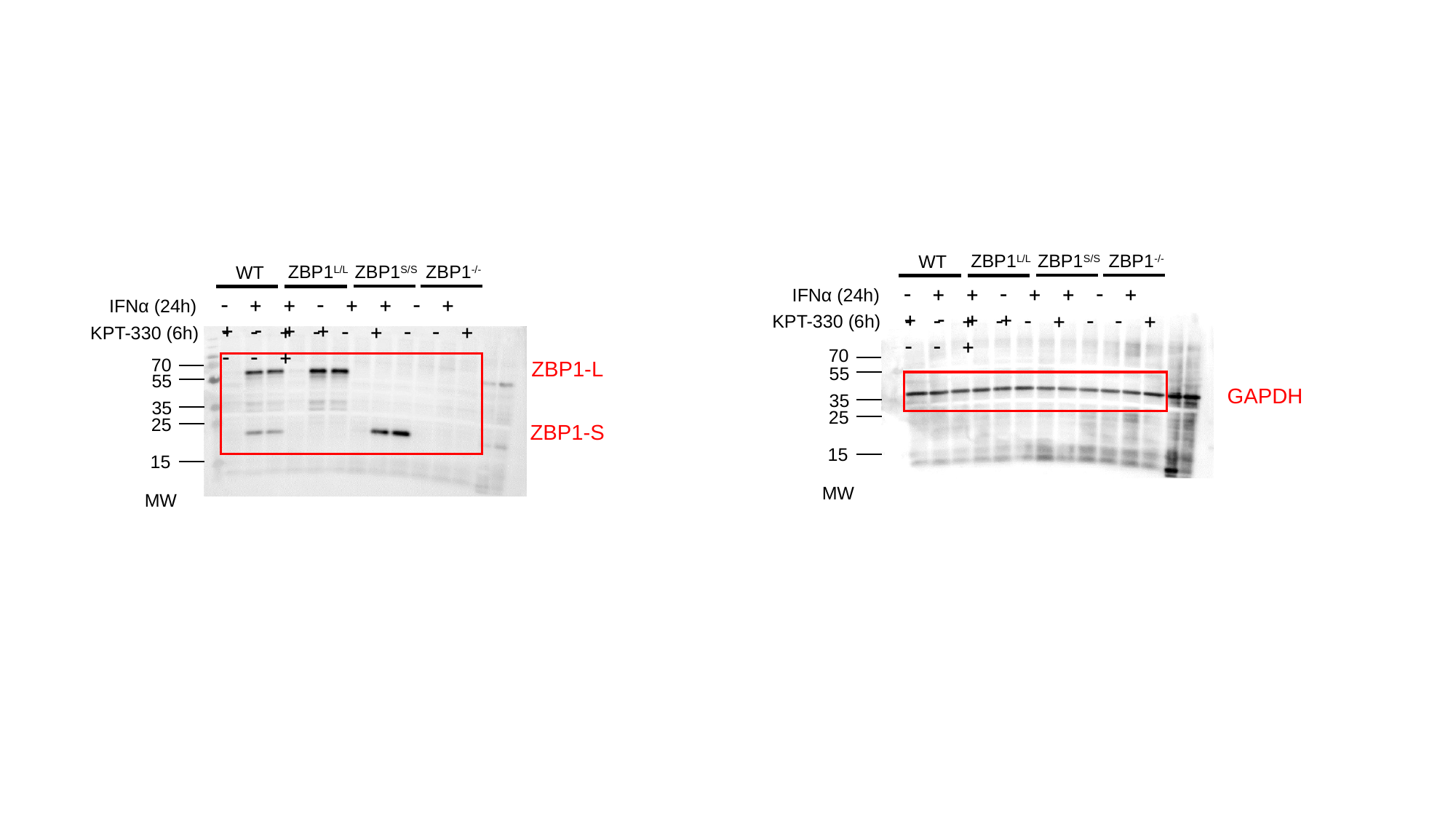

ZBP1-/-
ZBP1S/S
ZBP1L/L
WT
ZBP1-/-
ZBP1S/S
ZBP1L/L
WT
- + + - + + - + + - + +
IFNα (24h)
- + + - + + - + + - + +
IFNα (24h)
- - + - - + - - + - - +
KPT-330 (6h)
- - + - - + - - + - - +
KPT-330 (6h)
70
70
ZBP1-L
55
55
GAPDH
35
35
25
25
ZBP1-S
15
15
MW
MW

Supplement: Supplementary file 4 — Source data Fig. 2 [file 44318_2024_238_MOESM4_ESM.zip › Figure 2/2G/western ZBP1 GAPDH.pptx]

## Slide 1
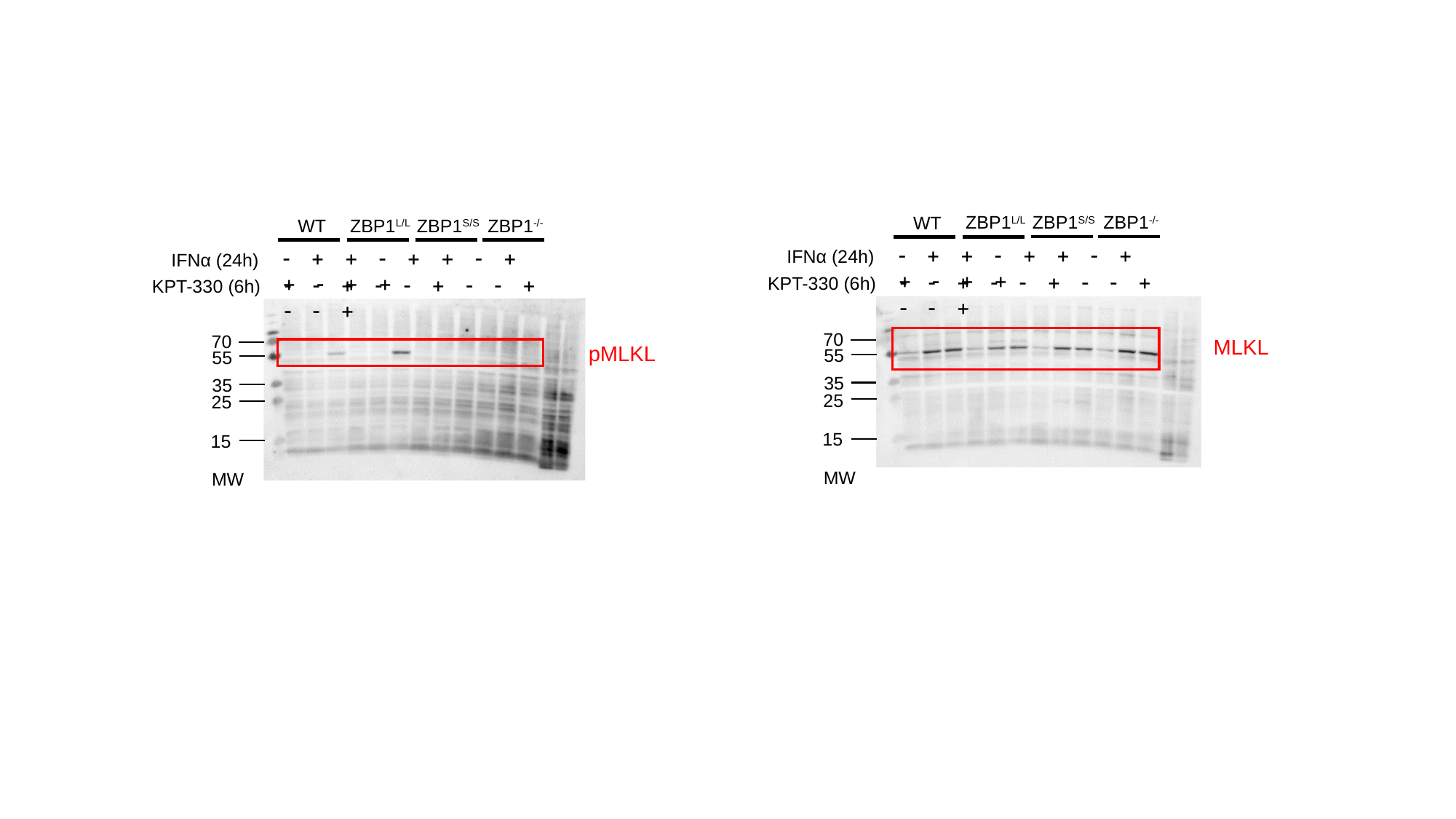

ZBP1-/-
ZBP1S/S
ZBP1L/L
WT
ZBP1-/-
ZBP1S/S
ZBP1L/L
WT
- + + - + + - + + - + +
IFNα (24h)
- + + - + + - + + - + +
IFNα (24h)
- - + - - + - - + - - +
KPT-330 (6h)
- - + - - + - - + - - +
KPT-330 (6h)
70
70
MLKL
pMLKL
55
55
35
35
25
25
15
15
MW
MW

Supplement: Supplementary file 4 — Source data Fig. 2 [file 44318_2024_238_MOESM4_ESM.zip › Figure 2/2G/western pMLKL MLKL.pptx]

## Slide 1
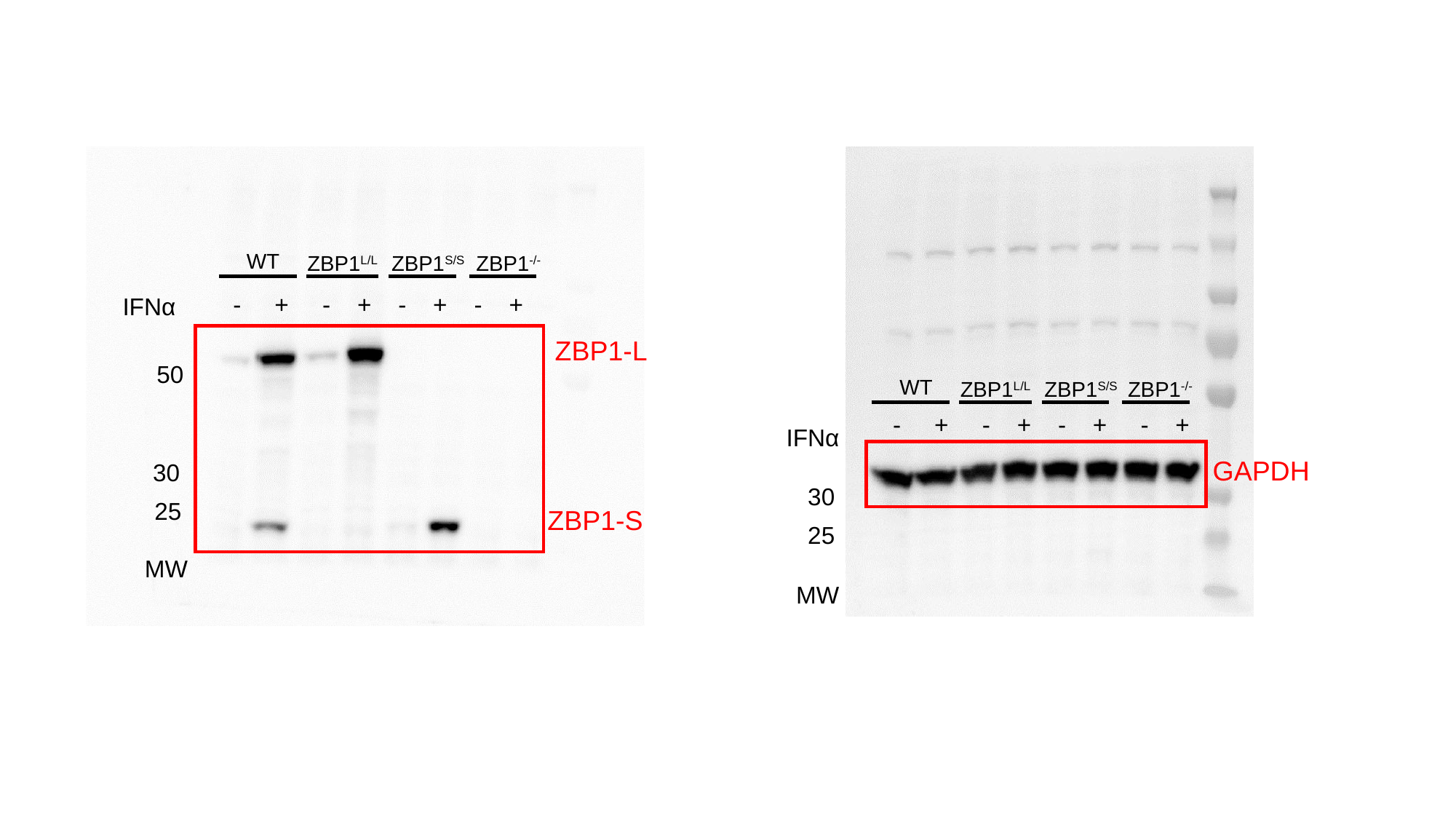

GAPDH
WT
ZBP1-/-
ZBP1L/L
ZBP1S/S
- + - + - + - +
IFNα
ZBP1-L
50
WT
ZBP1L/L
ZBP1S/S
ZBP1-/-
- + - + - + - +
IFNα
30
30
25
ZBP1-S
25
MW
MW

Supplement: Supplementary file 4 — Source data Fig. 2 [file 44318_2024_238_MOESM4_ESM.zip › Figure 2/2C/western ZBP1 GAPDH.pptx]

## Slide 1
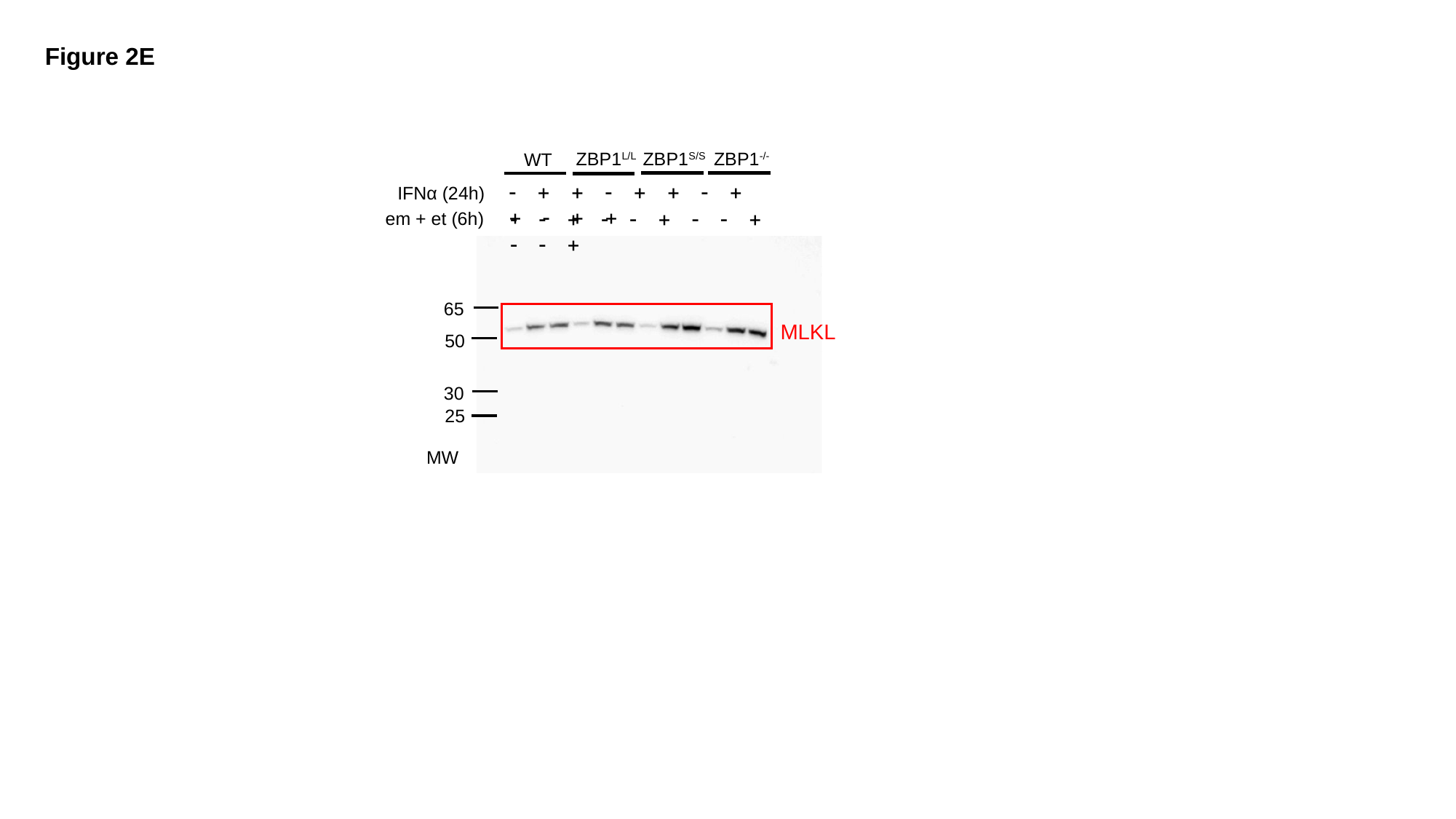

Figure 2E
ZBP1-/-
ZBP1S/S
ZBP1L/L
WT
- + + - + + - + + - + +
IFNα (24h)
- - + - - + - - + - - +
em + et (6h)
65
MLKL
50
30
25
MW

Supplement: Supplementary file 4 — Source data Fig. 2 [file 44318_2024_238_MOESM4_ESM.zip › Figure 2/2E/western MLKL.pptx]

## Slide 1
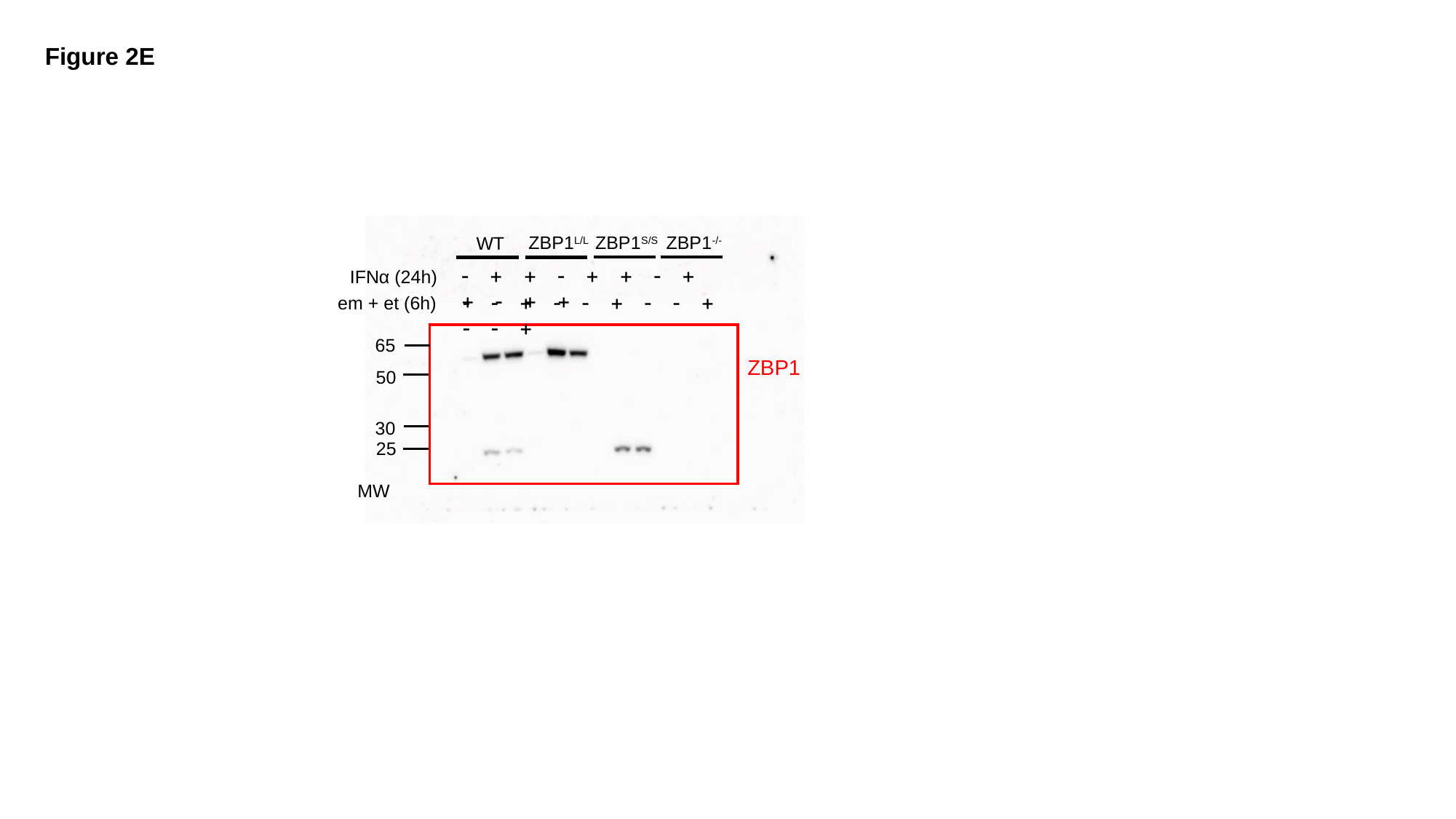

Figure 2E
ZBP1-/-
ZBP1S/S
ZBP1L/L
WT
- + + - + + - + + - + +
IFNα (24h)
- - + - - + - - + - - +
em + et (6h)
65
ZBP1
50
30
25
MW

Supplement: Supplementary file 4 — Source data Fig. 2 [file 44318_2024_238_MOESM4_ESM.zip › Figure 2/2E/western ZBP1.pptx]

## Slide 1
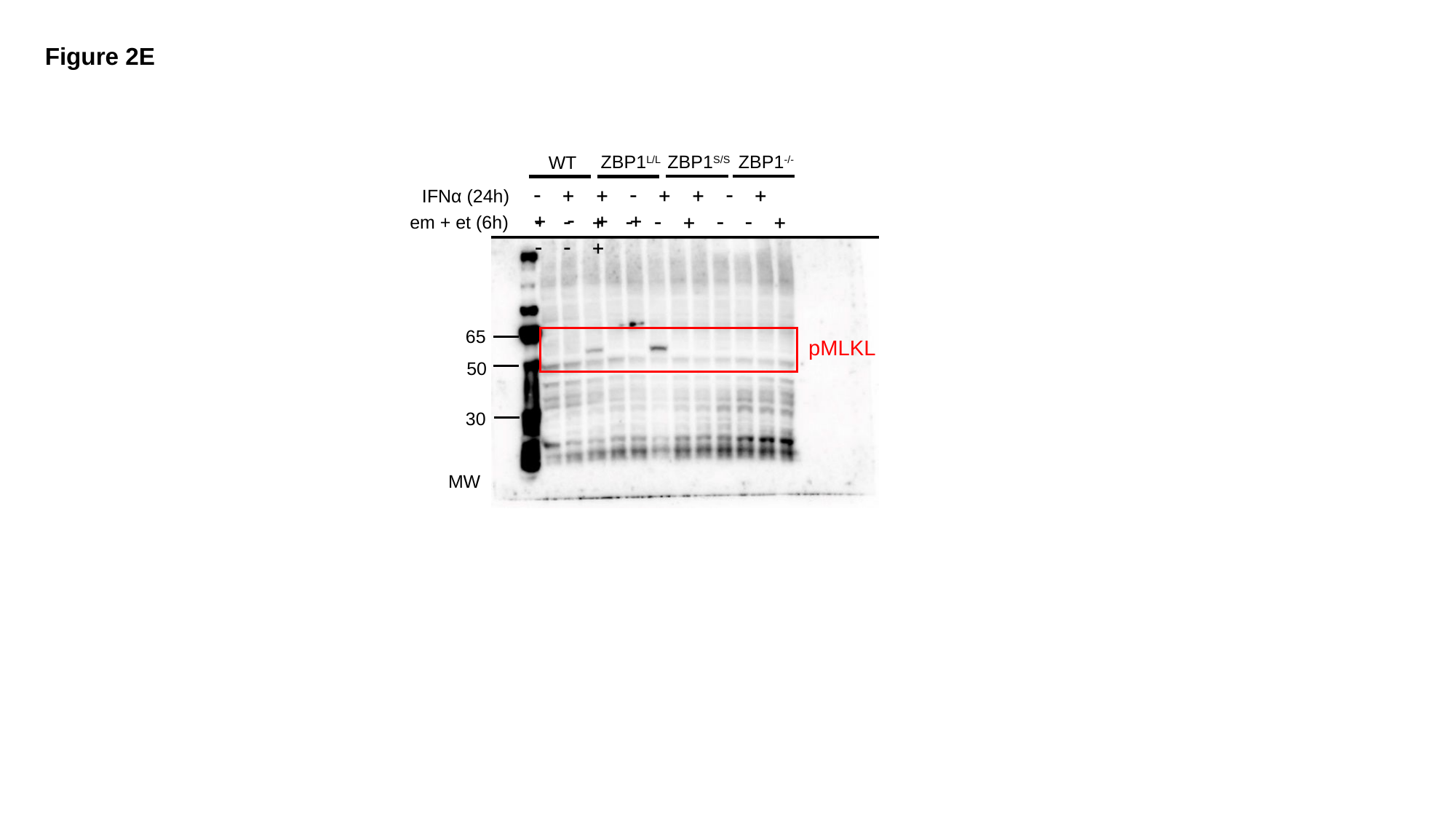

Figure 2E
ZBP1-/-
ZBP1S/S
ZBP1L/L
WT
- + + - + + - + + - + +
IFNα (24h)
- - + - - + - - + - - +
em + et (6h)
65
pMLKL
50
30
MW

Supplement: Supplementary file 4 — Source data Fig. 2 [file 44318_2024_238_MOESM4_ESM.zip › Figure 2/2E/western pMLKL.pptx]

## Slide 1
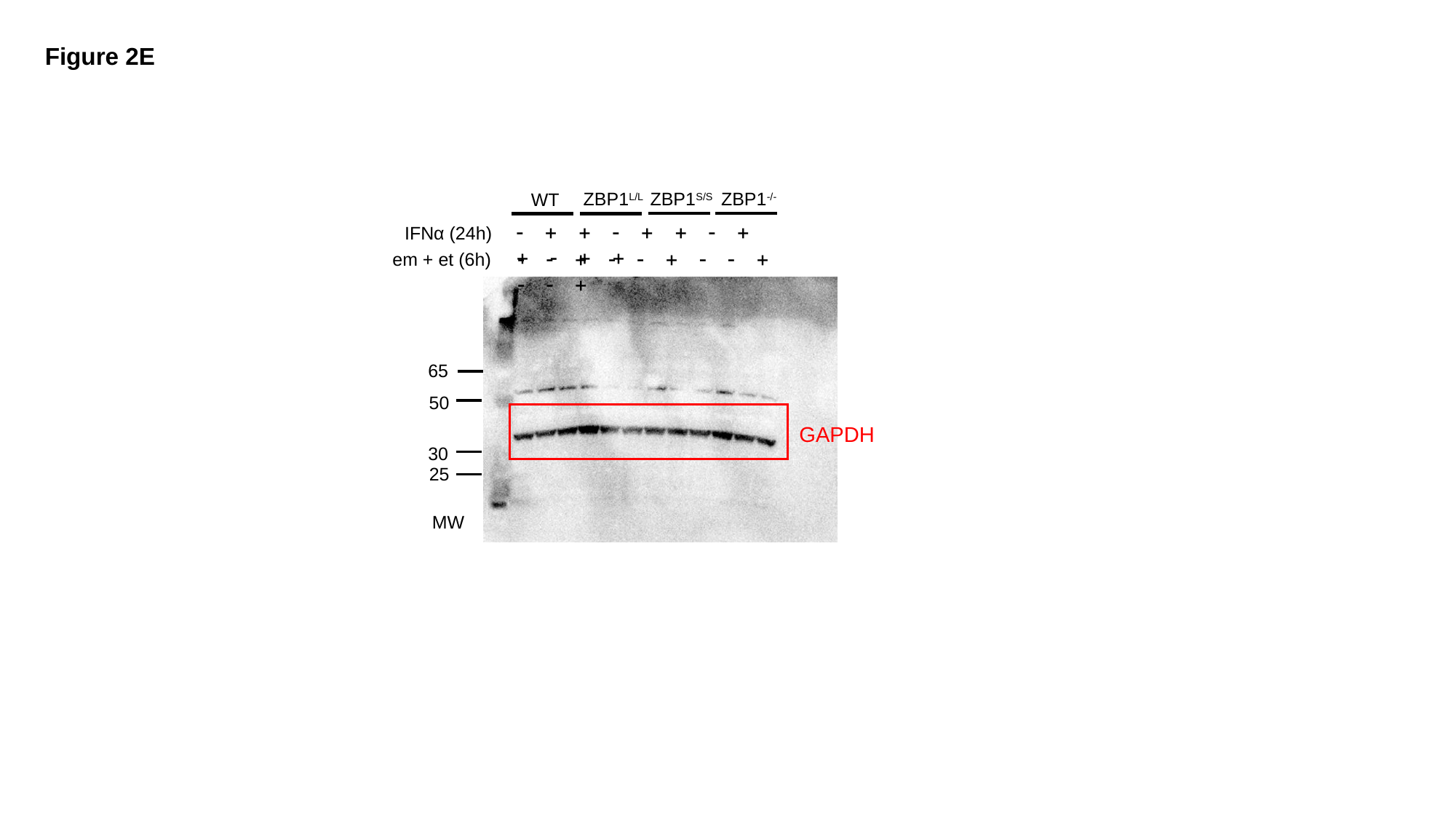

Figure 2E
ZBP1-/-
ZBP1S/S
ZBP1L/L
WT
- + + - + + - + + - + +
IFNα (24h)
- - + - - + - - + - - +
em + et (6h)
65
50
GAPDH
30
25
MW

Supplement: Supplementary file 4 — Source data Fig. 2 [file 44318_2024_238_MOESM4_ESM.zip › Figure 2/2E/western GAPDH.pptx]

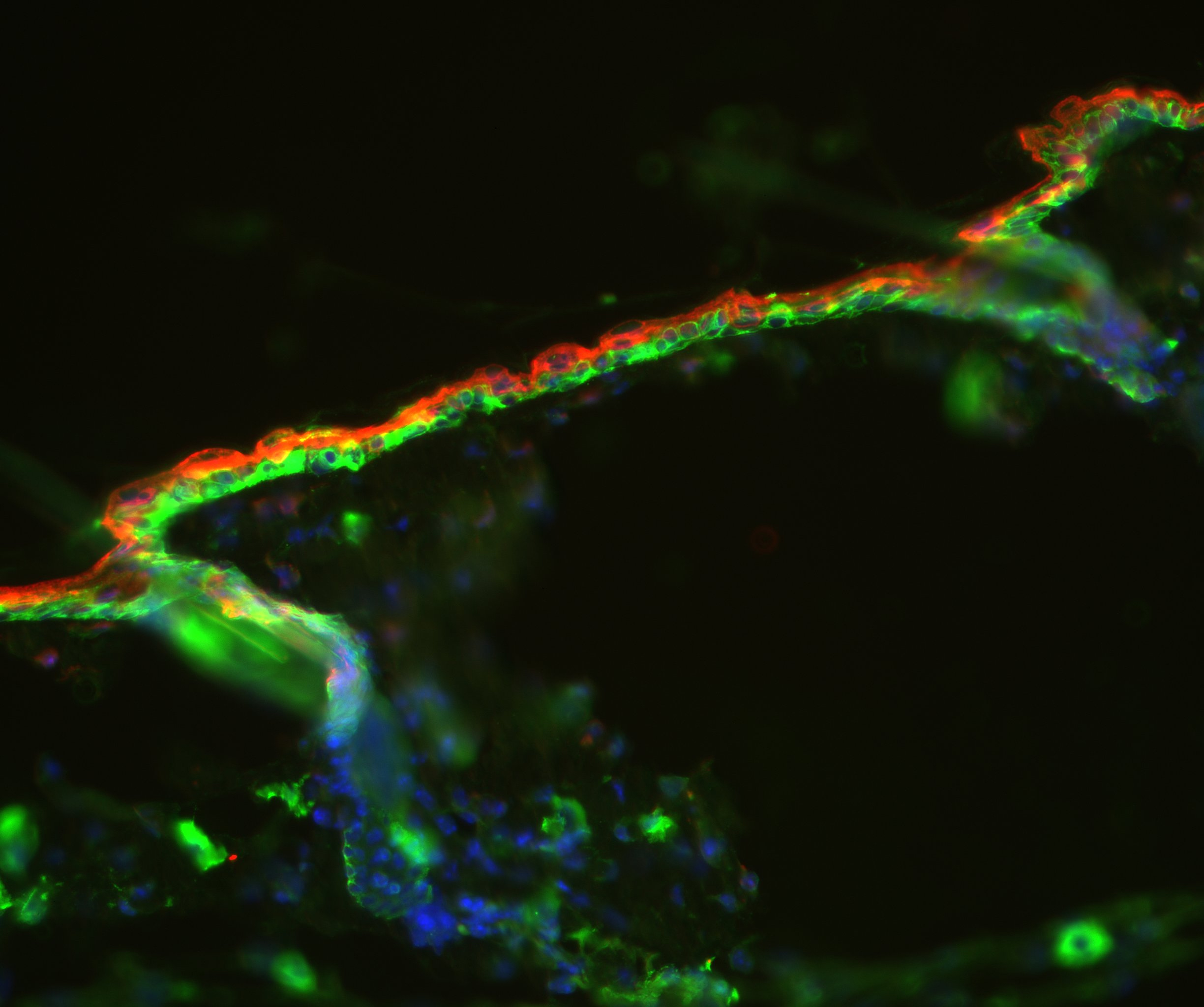

Supplement: Supplementary file 5 — Source data Fig. 3 [file 44318_2024_238_MOESM5_ESM.zip › Figure 3/3D/RIPK1 EKO; ZBP1 S_S K10_K14_Hoechst copy.jpg]

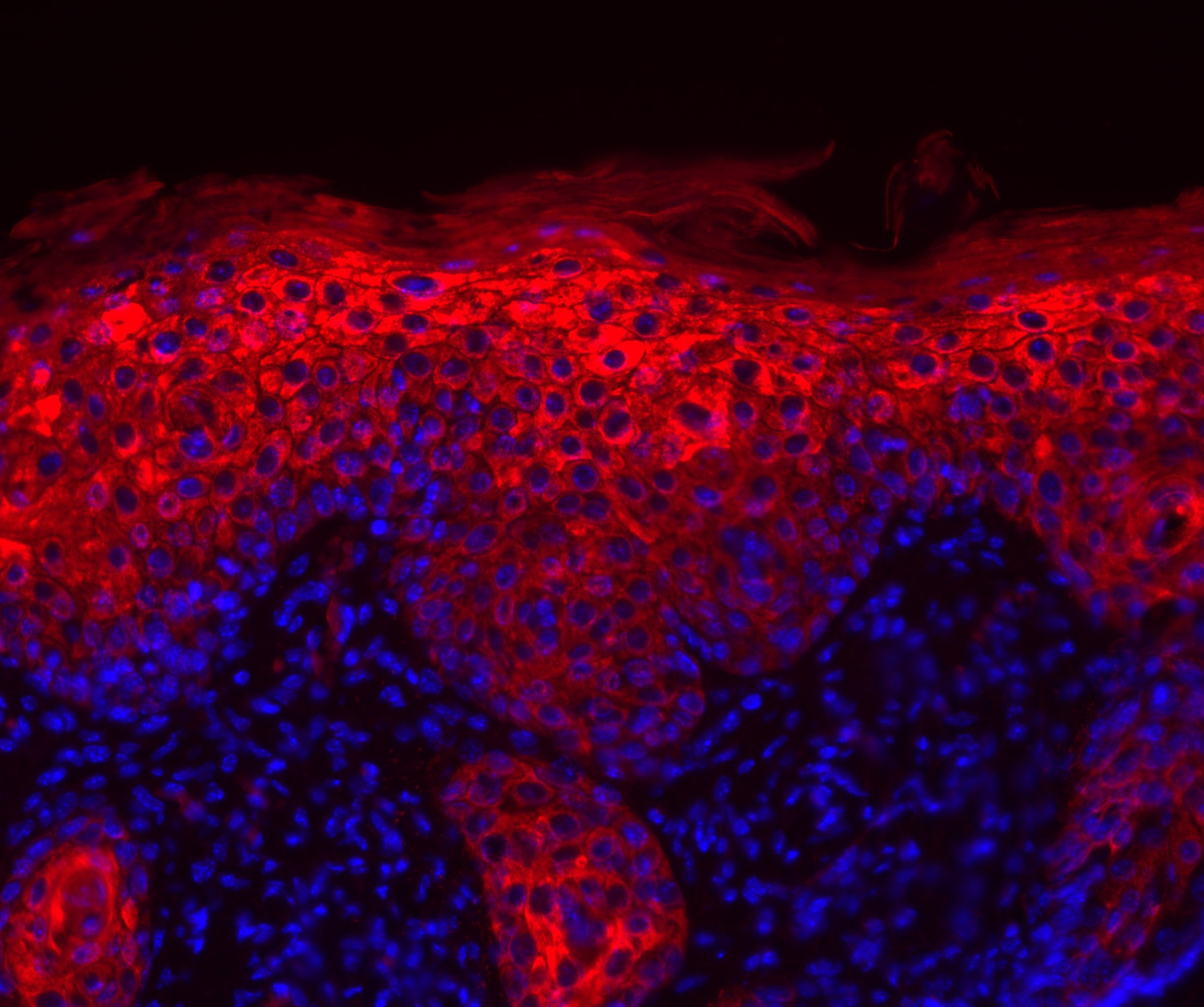

Supplement: Supplementary file 5 — Source data Fig. 3 [file 44318_2024_238_MOESM5_ESM.zip › Figure 3/3D/RIPK1 EKO; ZBP1 WT_WT K6_Hoechst copy.jpg]

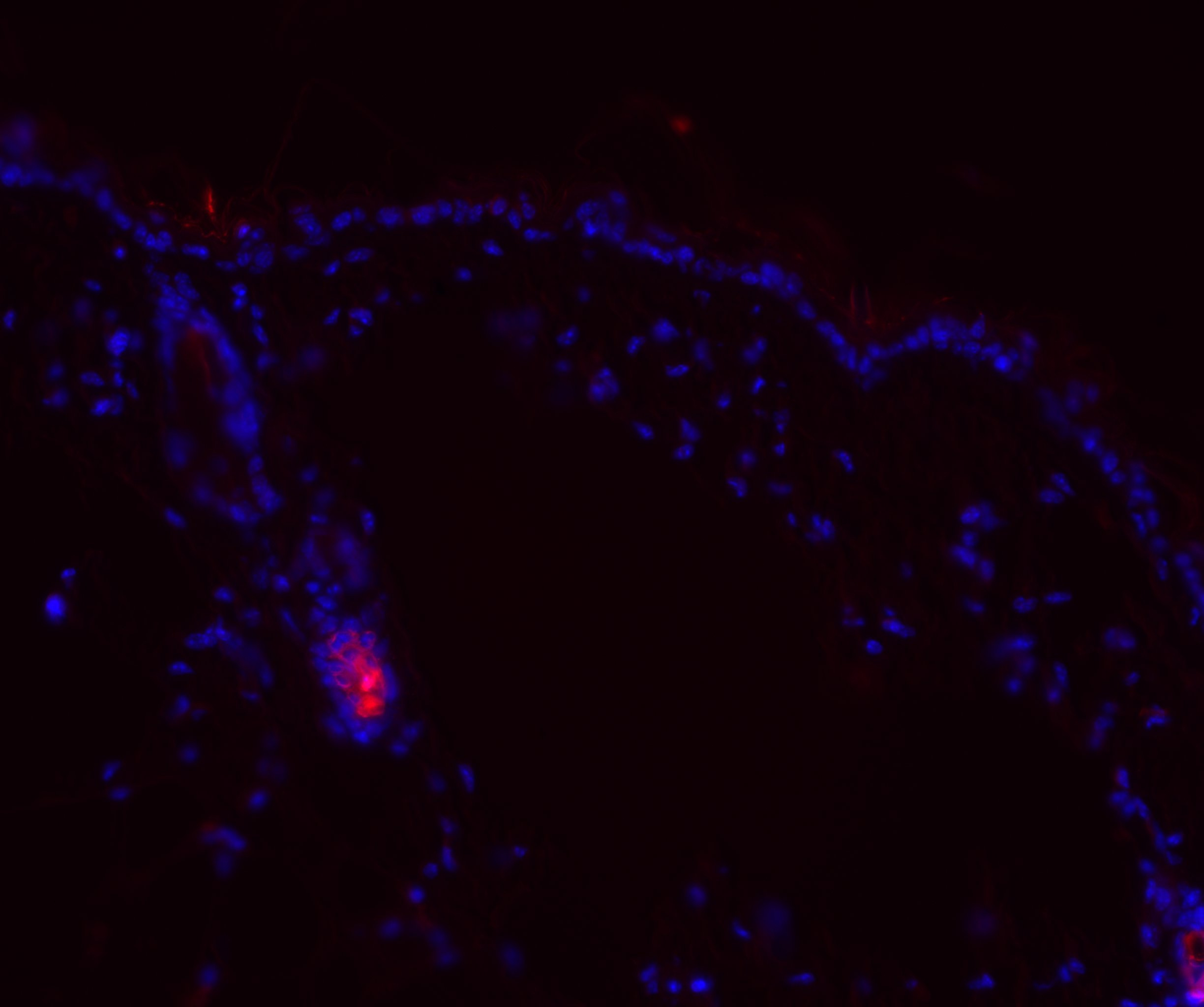

Supplement: Supplementary file 5 — Source data Fig. 3 [file 44318_2024_238_MOESM5_ESM.zip › Figure 3/3D/Ctrl K6_Hoechst copy.jpg]

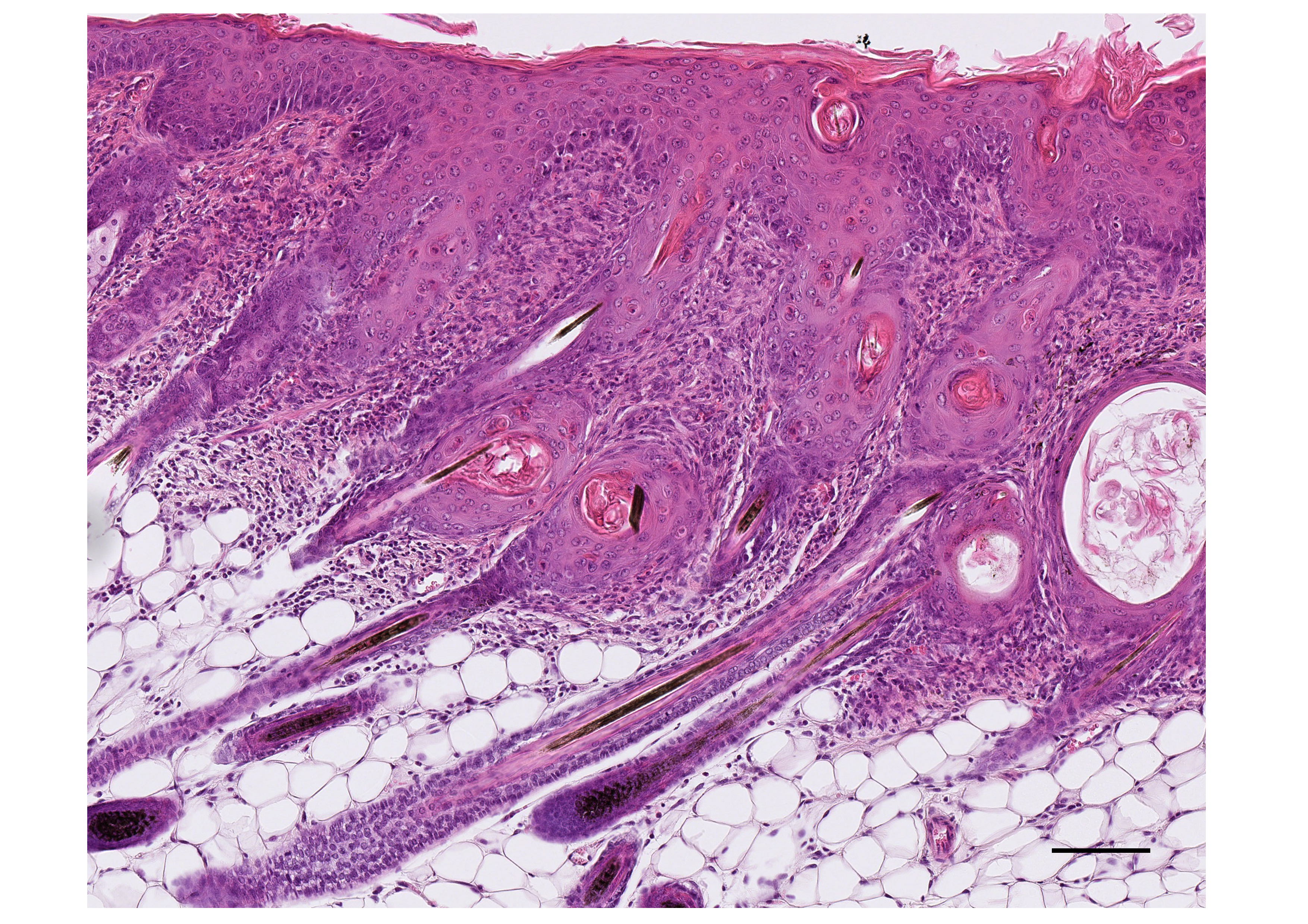

Supplement: Supplementary file 5 — Source data Fig. 3 [file 44318_2024_238_MOESM5_ESM.zip › Figure 3/3D/RIPK1 EKO; ZBP1 WT_WT HE copy.jpg]

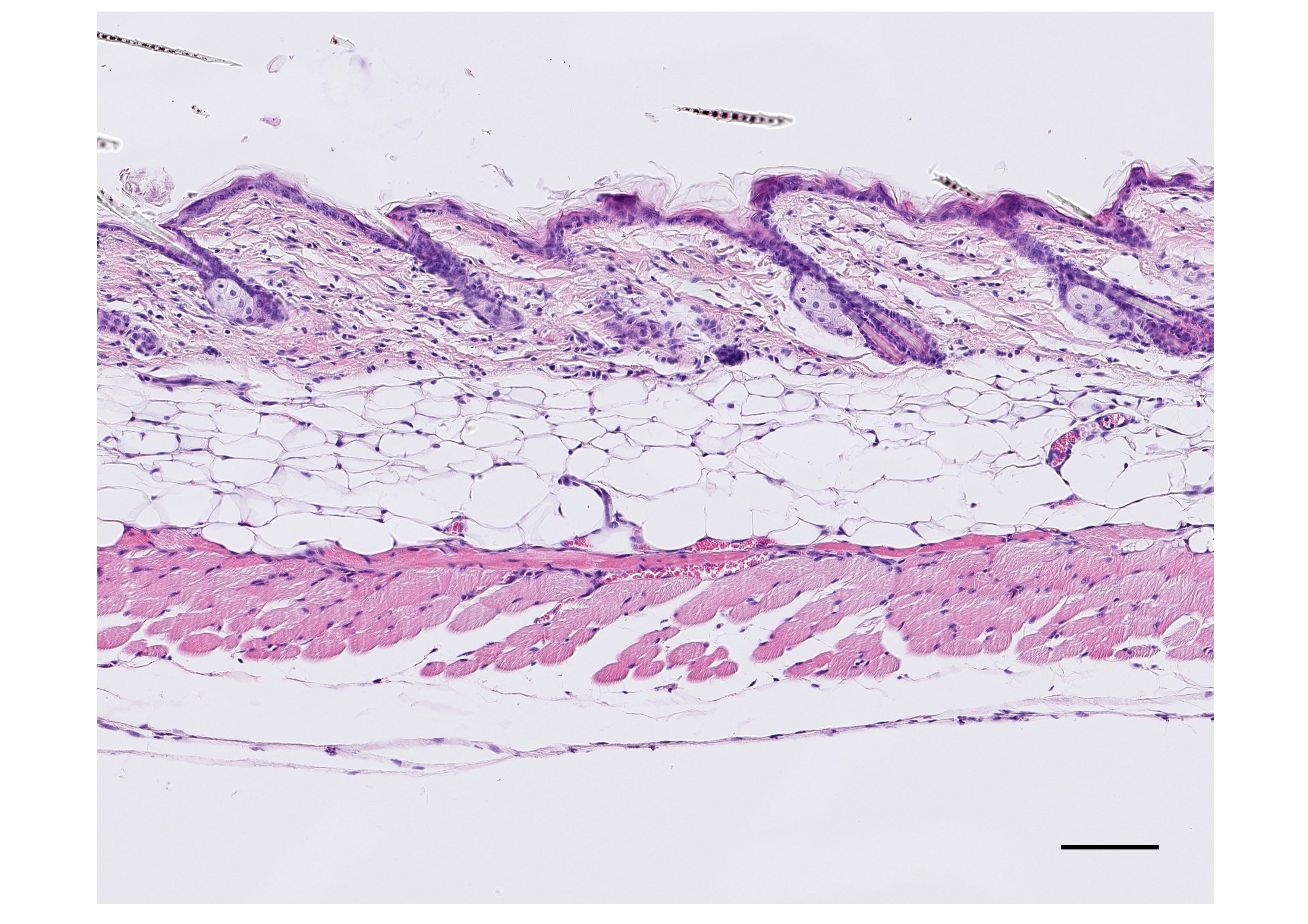

Supplement: Supplementary file 5 — Source data Fig. 3 [file 44318_2024_238_MOESM5_ESM.zip › Figure 3/3D/RIPK1 EKO; ZBP1 S_S HE copy.jpg]

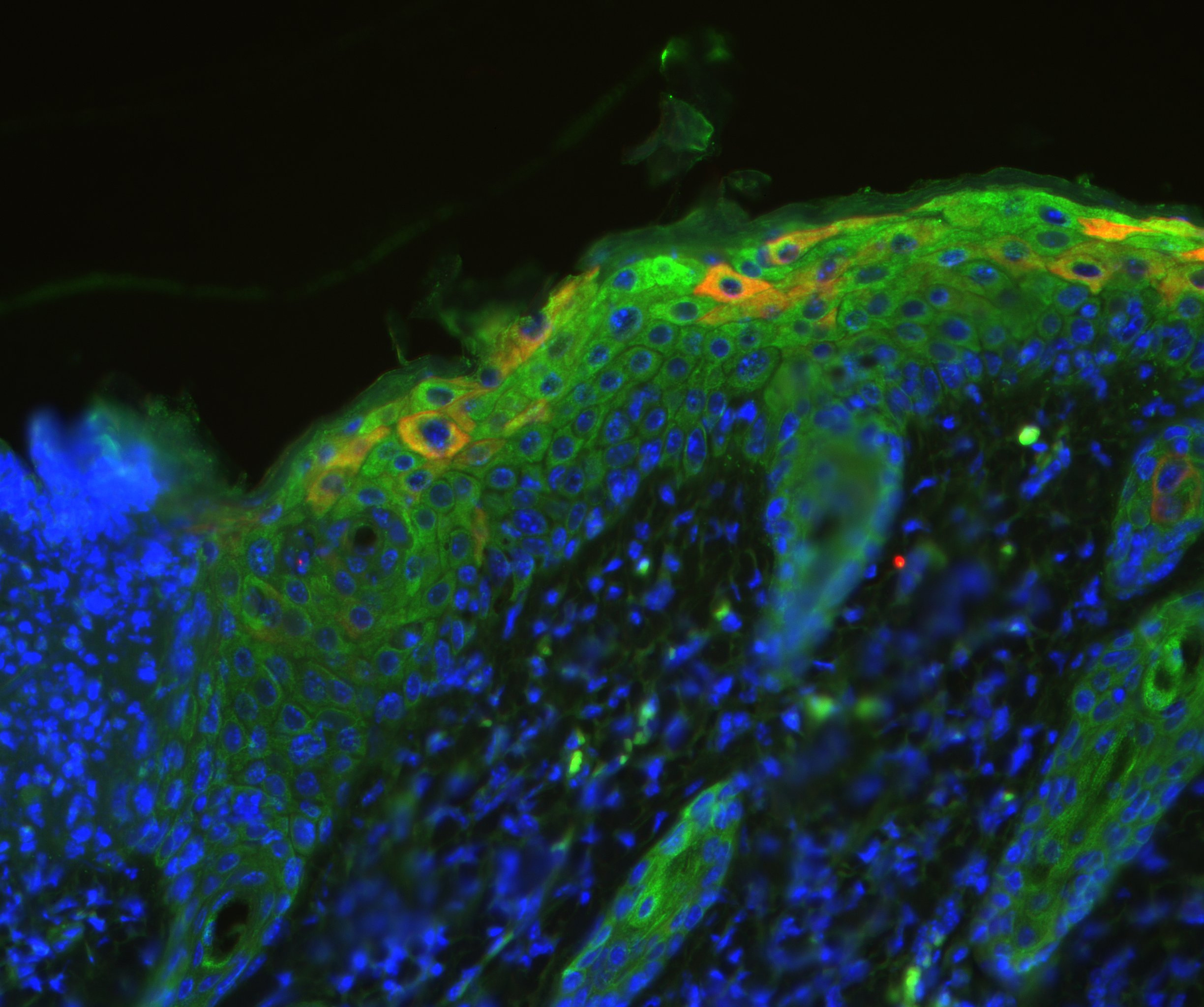

Supplement: Supplementary file 5 — Source data Fig. 3 [file 44318_2024_238_MOESM5_ESM.zip › Figure 3/3D/RIPK1 EKO; ZBP1 WT_WT K10_K14_Hoechst copy.jpg]

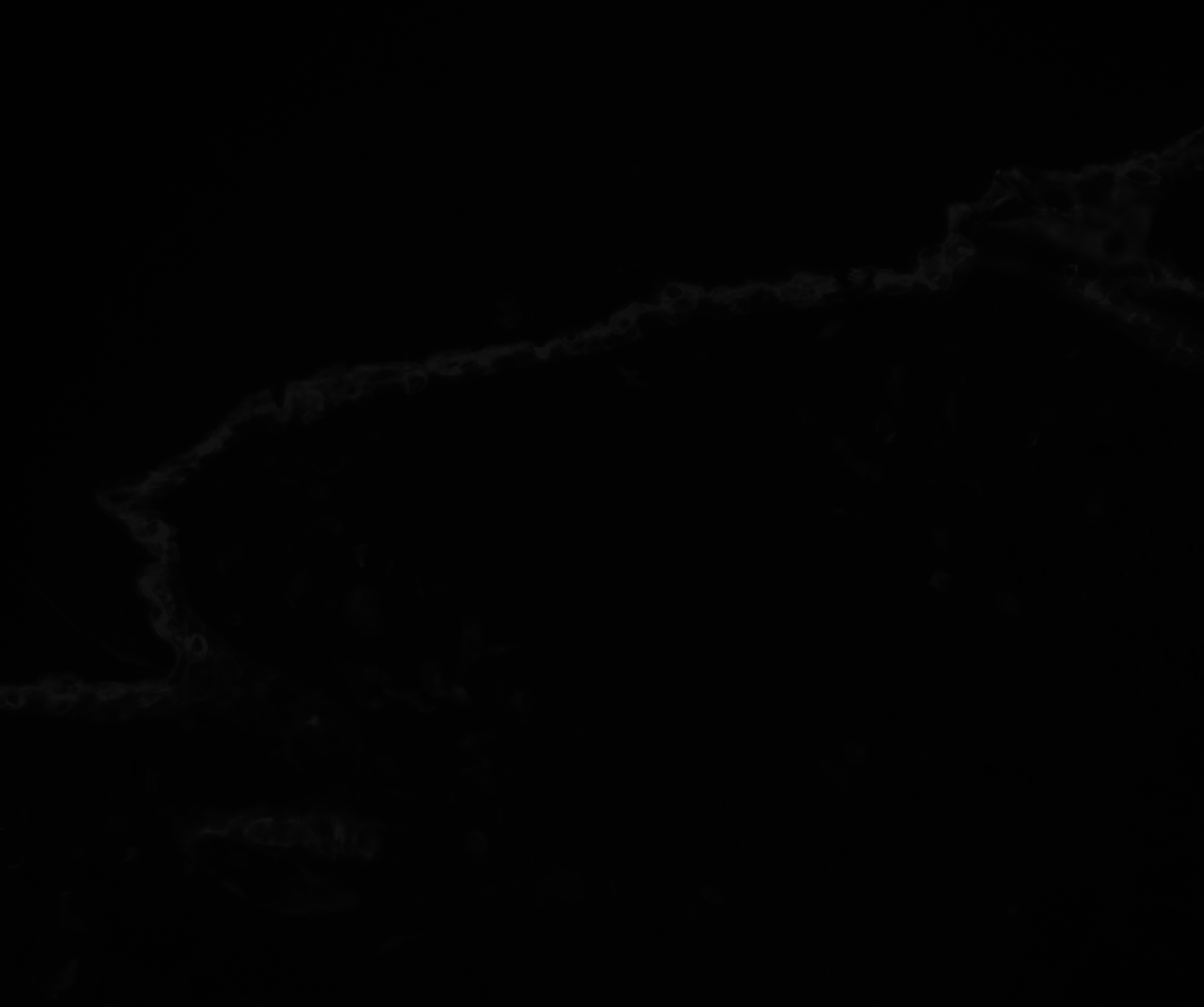

Supplement: Supplementary file 5 — Source data Fig. 3 [file 44318_2024_238_MOESM5_ESM.zip › Figure 3/3D/RIPK1 EKO; ZBP1 WT_S K10_K14_Hoechst copy.jpg]

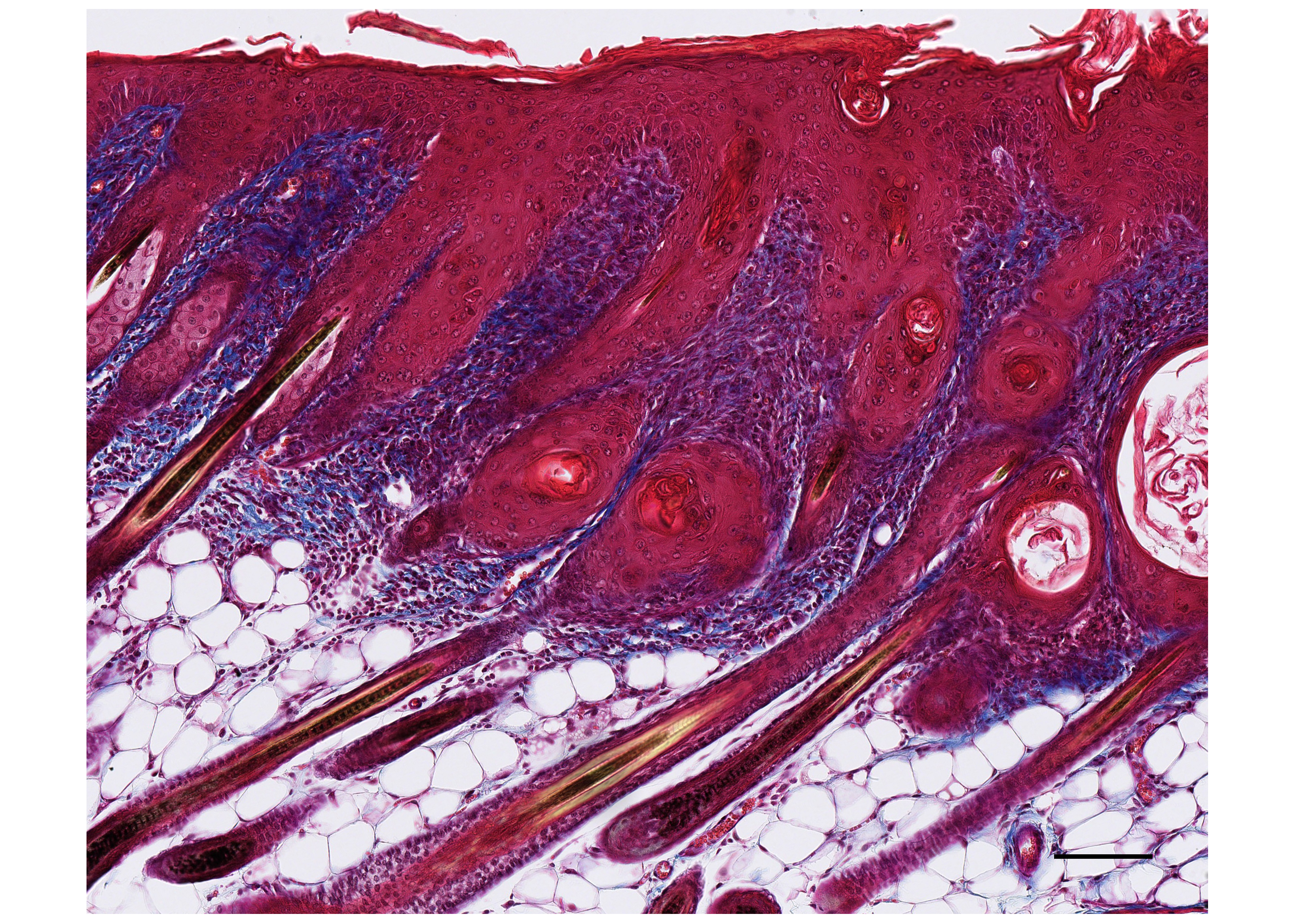

Supplement: Supplementary file 5 — Source data Fig. 3 [file 44318_2024_238_MOESM5_ESM.zip › Figure 3/3D/RIPK1 EKO; ZBP1 WT_WT Trichrome copy.jpg]

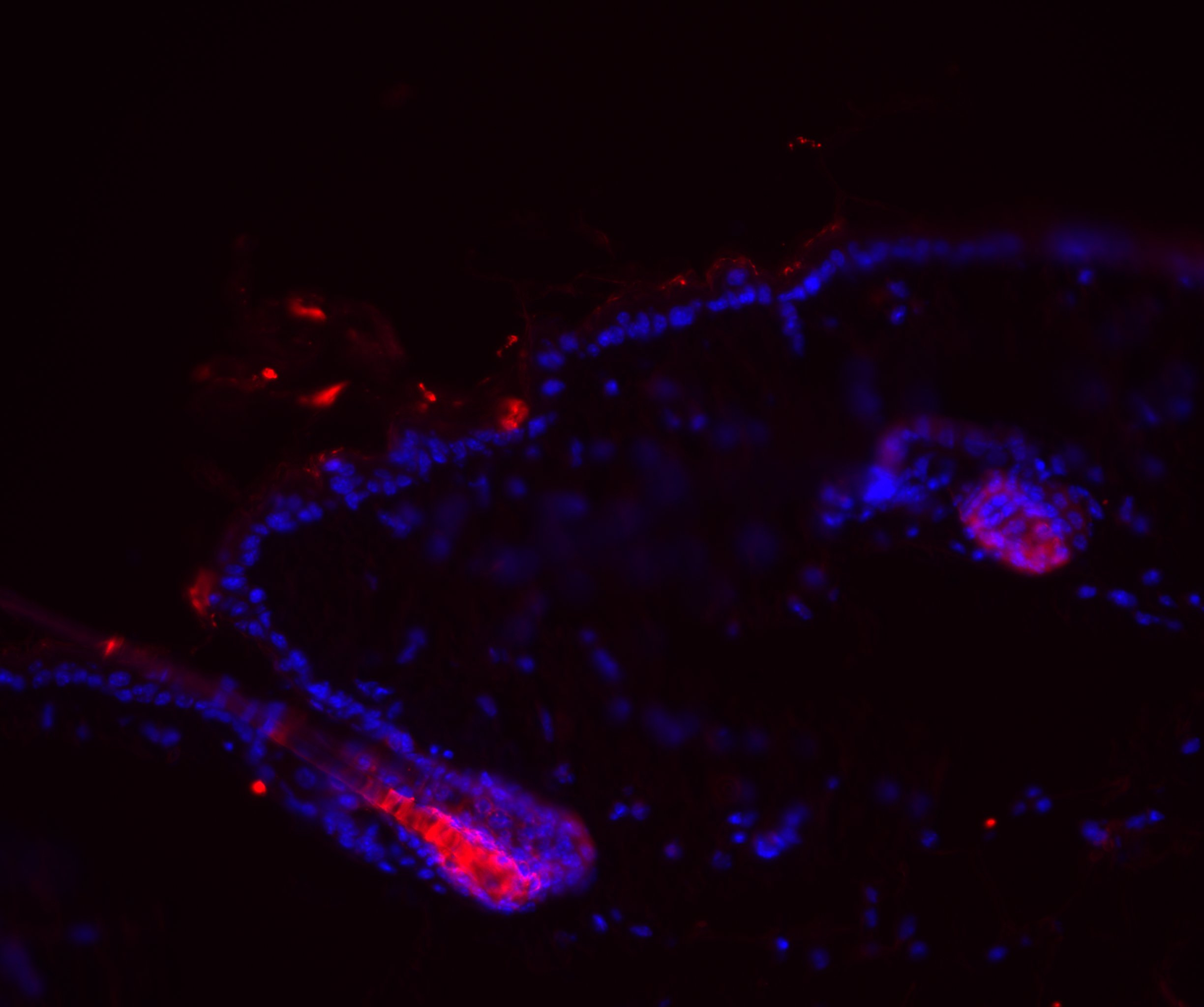

Supplement: Supplementary file 5 — Source data Fig. 3 [file 44318_2024_238_MOESM5_ESM.zip › Figure 3/3D/RIPK1 EKO; ZBP1 WT_S K6_Hoechst copy.jpg]

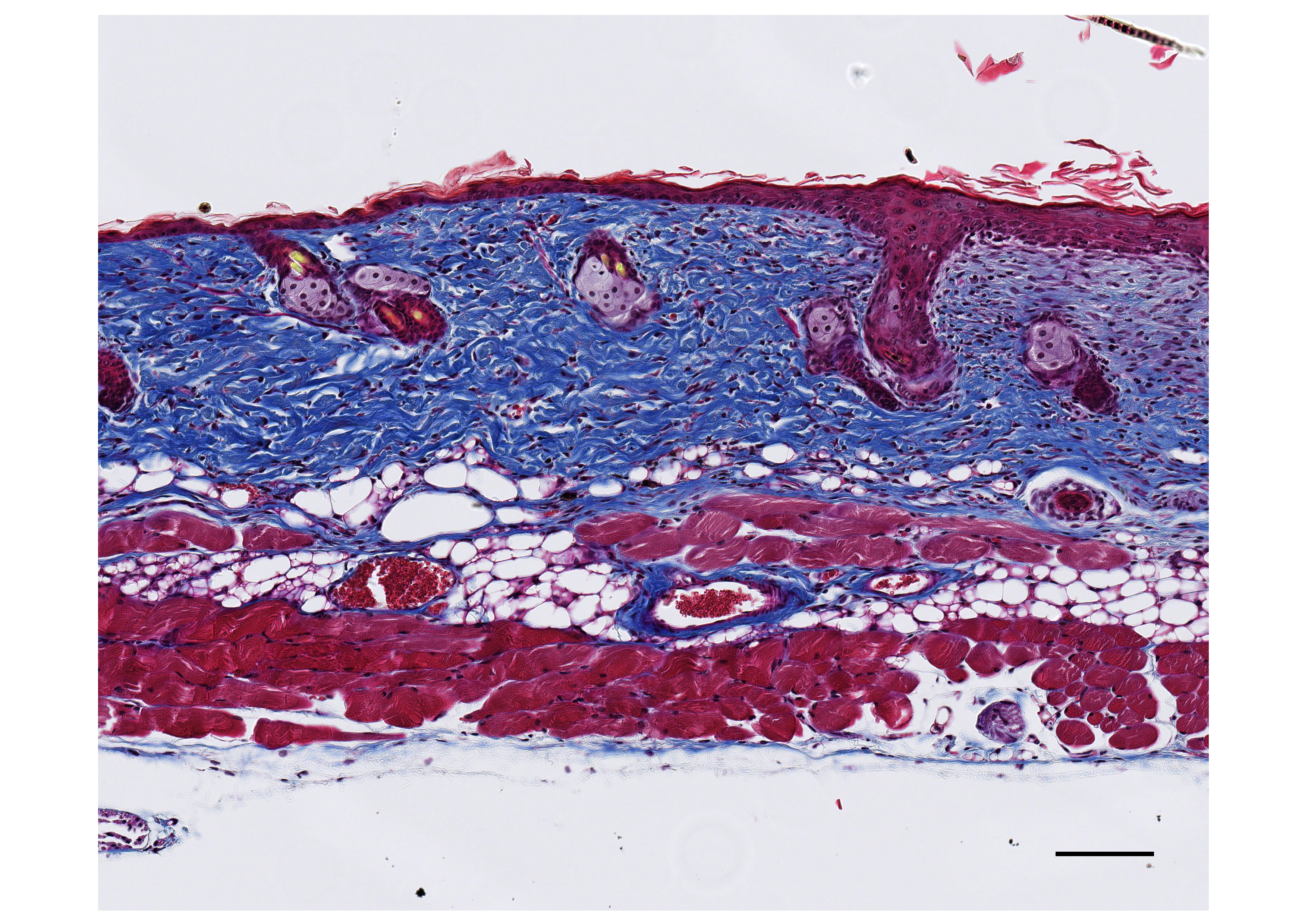

Supplement: Supplementary file 5 — Source data Fig. 3 [file 44318_2024_238_MOESM5_ESM.zip › Figure 3/3D/RIPK1 EKO; ZBP1 WT_S Trichrome copy.jpg]

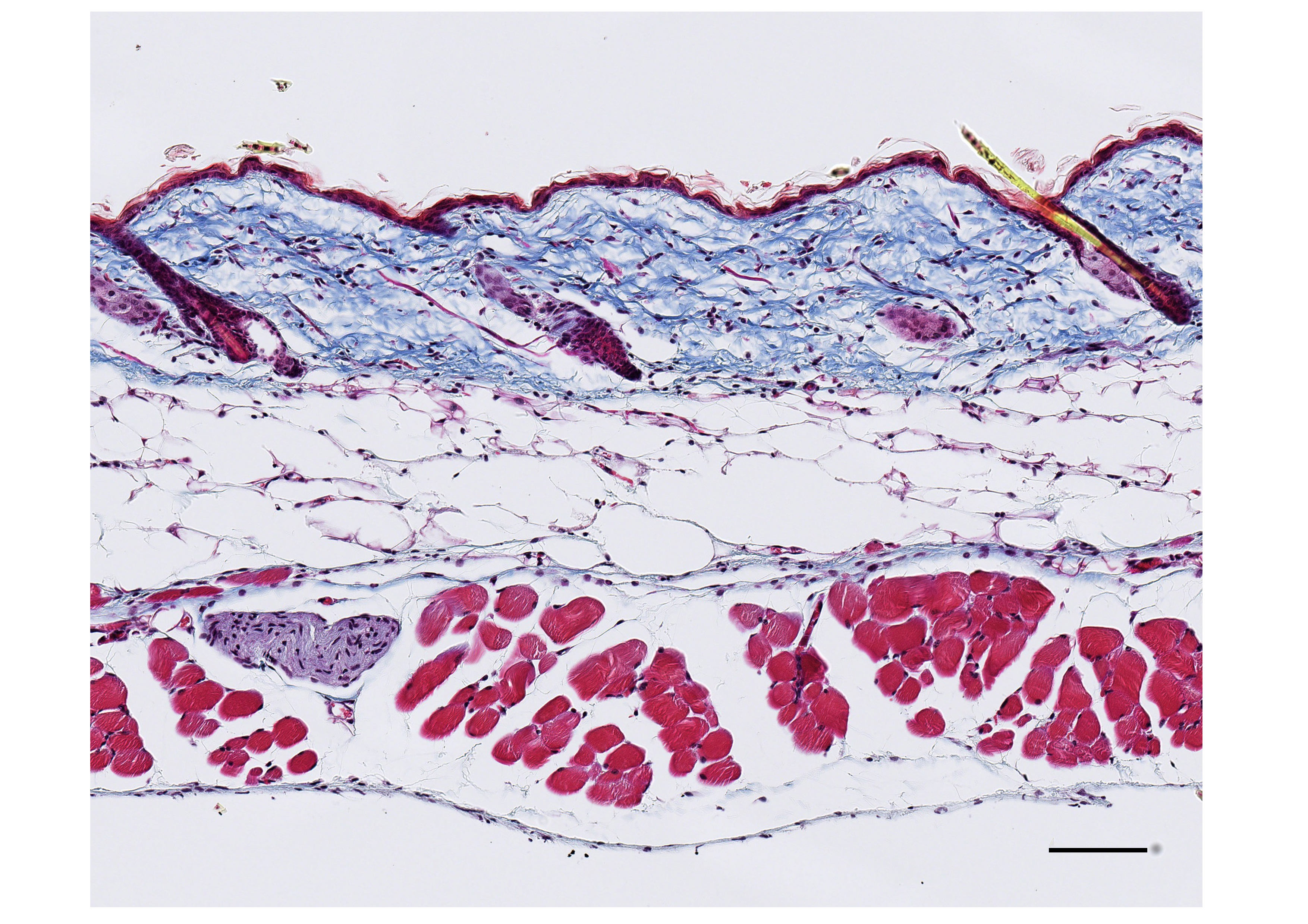

Supplement: Supplementary file 5 — Source data Fig. 3 [file 44318_2024_238_MOESM5_ESM.zip › Figure 3/3D/RIPK1 EKO; ZBP1 S_S Trichrome copy.jpg]

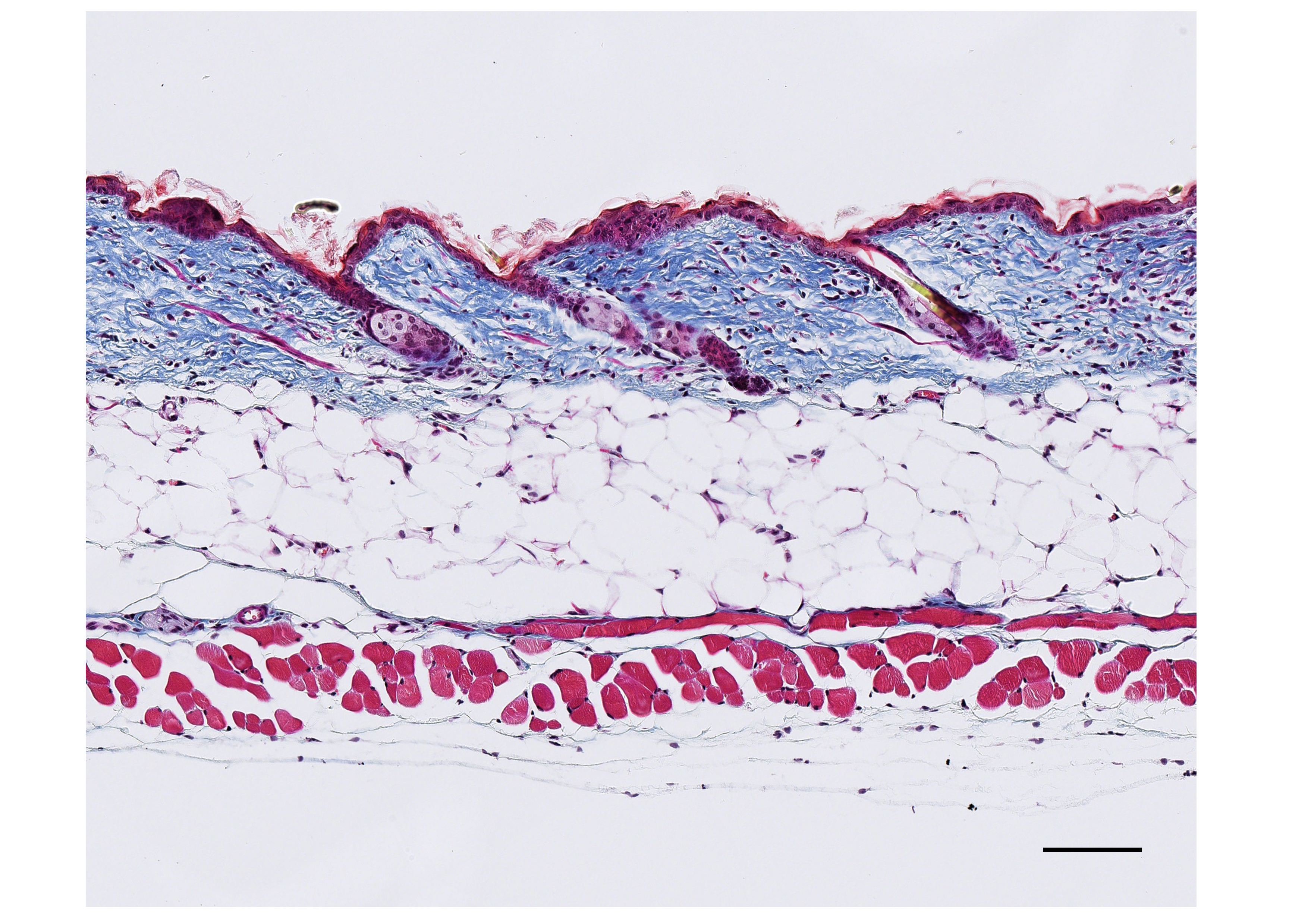

Supplement: Supplementary file 5 — Source data Fig. 3 [file 44318_2024_238_MOESM5_ESM.zip › Figure 3/3D/Control Trichrome copy.jpg]

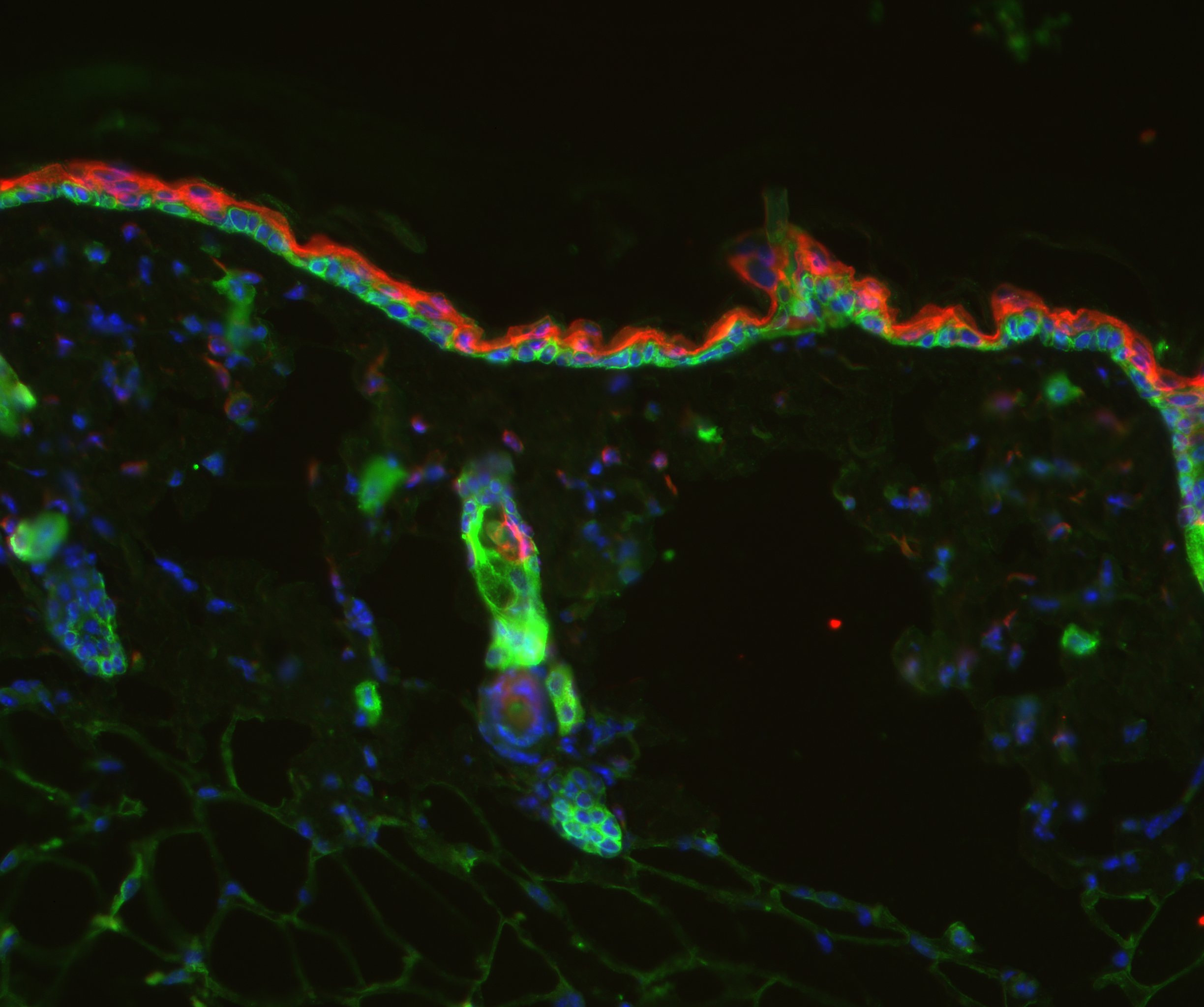

Supplement: Supplementary file 5 — Source data Fig. 3 [file 44318_2024_238_MOESM5_ESM.zip › Figure 3/3D/Ctrl K10_K14_Hoechst copy.jpg]

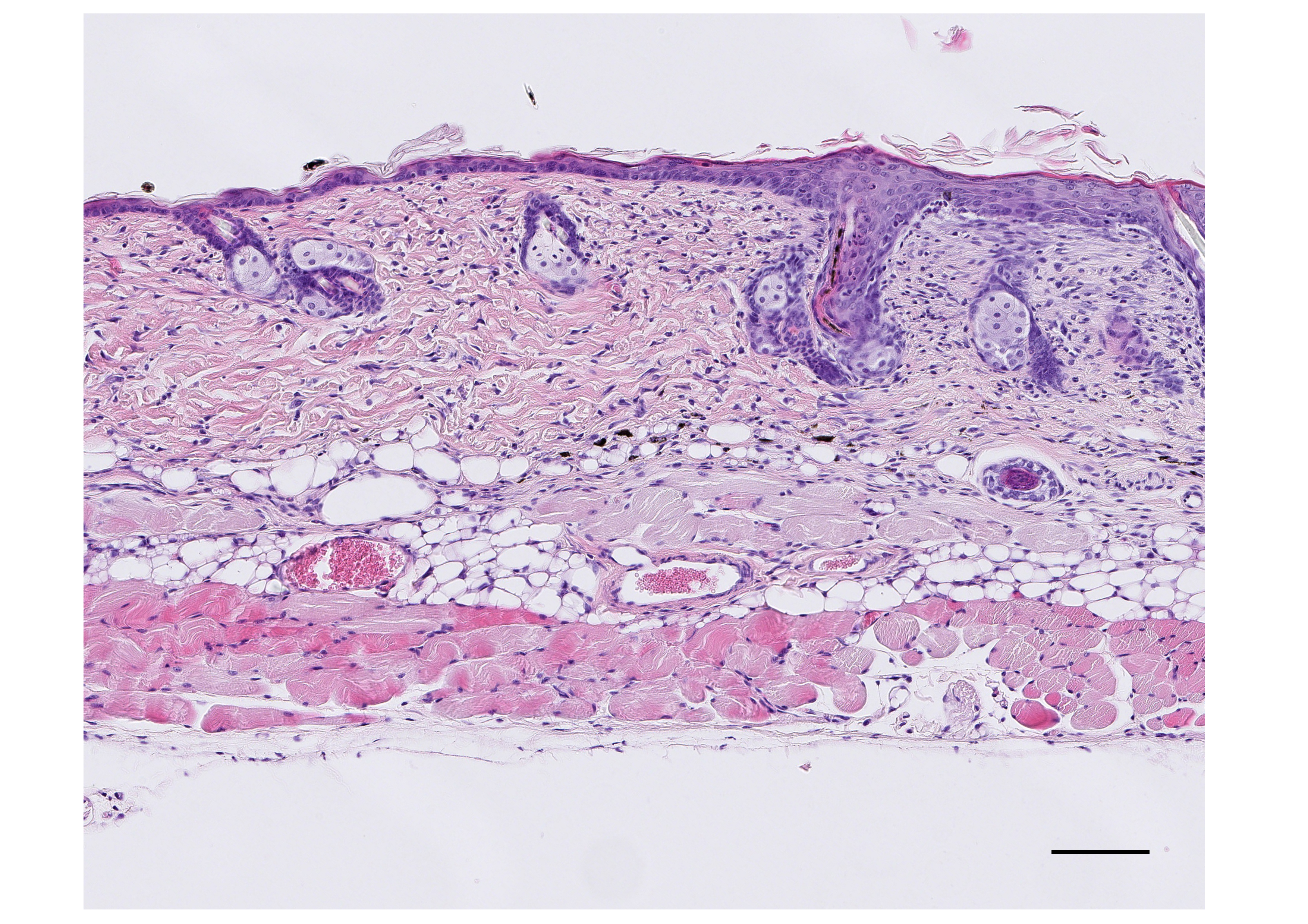

Supplement: Supplementary file 5 — Source data Fig. 3 [file 44318_2024_238_MOESM5_ESM.zip › Figure 3/3D/RIPK1 EKO; ZBP1 WT_S HE copy.jpg]

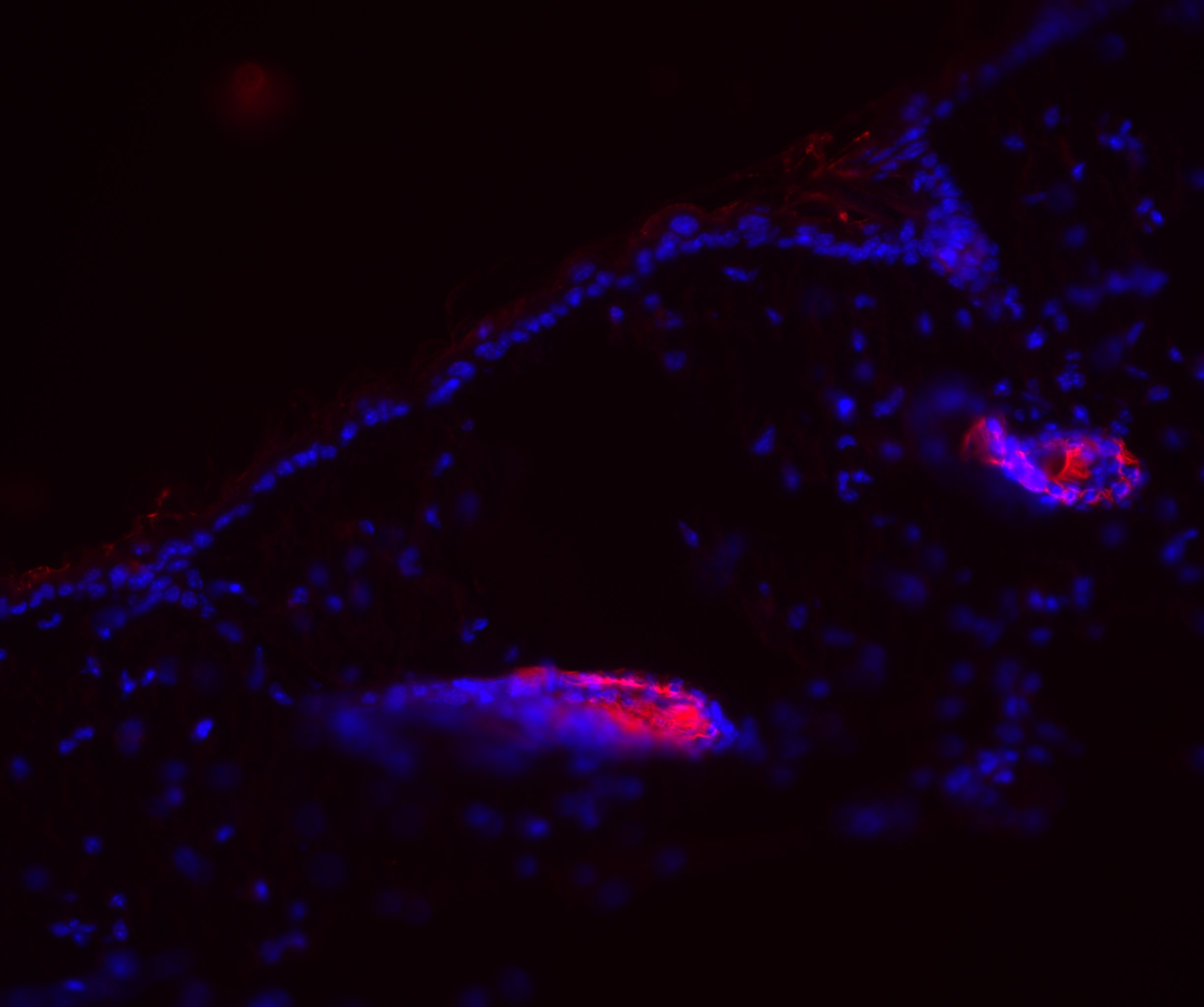

Supplement: Supplementary file 5 — Source data Fig. 3 [file 44318_2024_238_MOESM5_ESM.zip › Figure 3/3D/RIPK1 EKO; ZBP1 S_S K6_Hoechst copy.jpg]

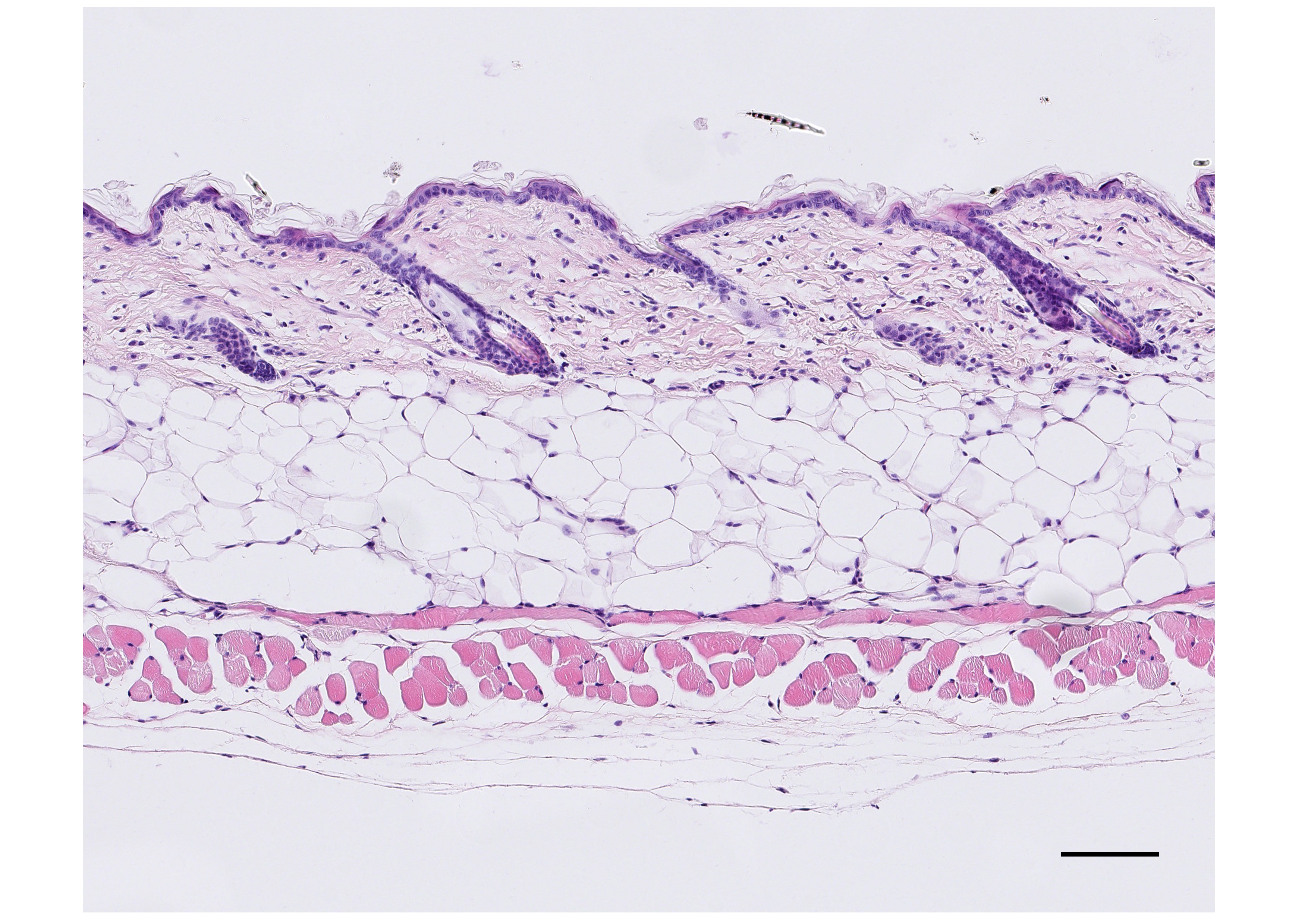

Supplement: Supplementary file 5 — Source data Fig. 3 [file 44318_2024_238_MOESM5_ESM.zip › Figure 3/3D/Control HE copy.jpg]

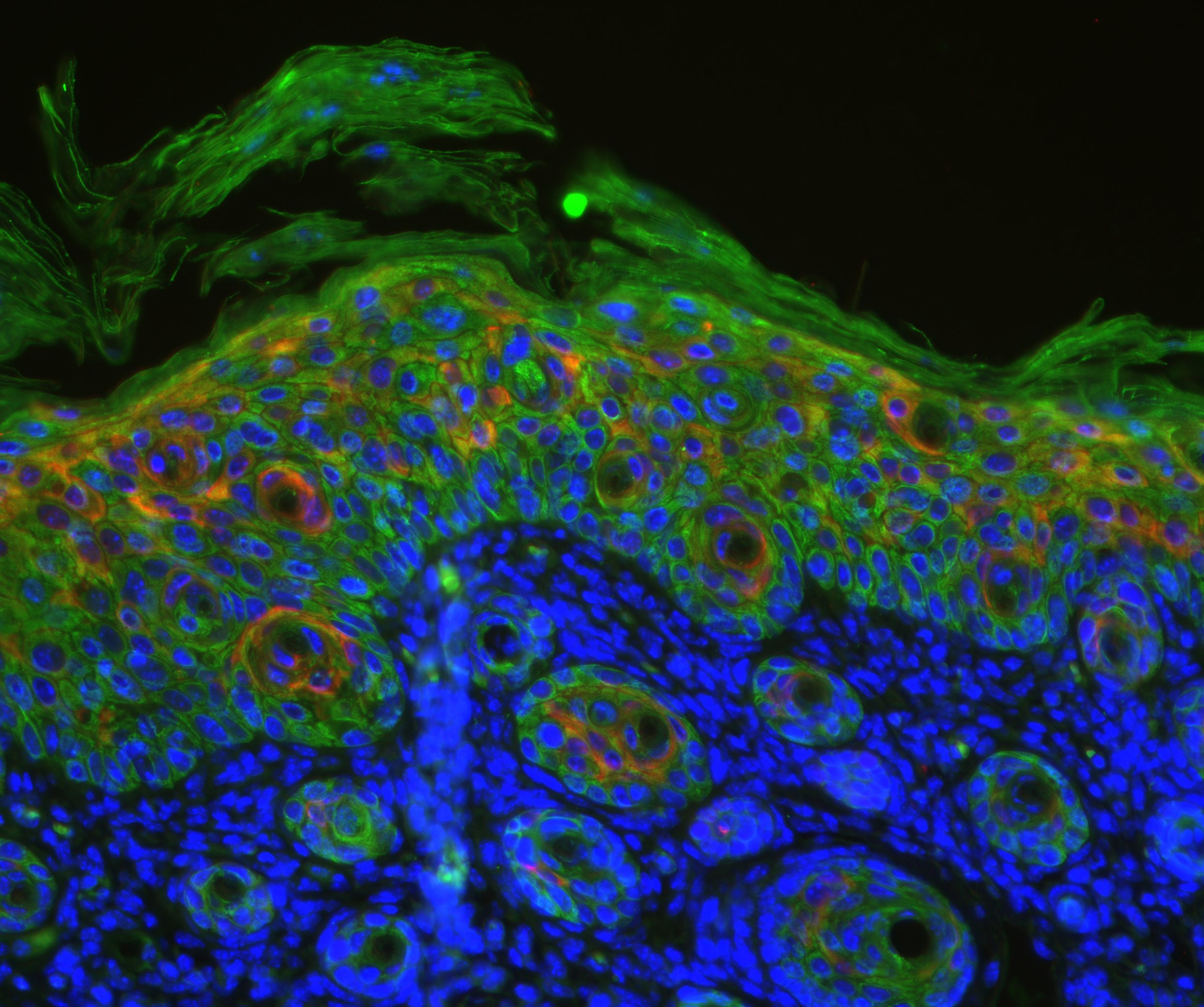

Supplement: Supplementary file 6 — Source data Fig. 4 [file 44318_2024_238_MOESM6_ESM.zip › Figure 4/4D/RIPK1 EKO; ZBP1 L_L K10K14_Hoechst copy.jpg]

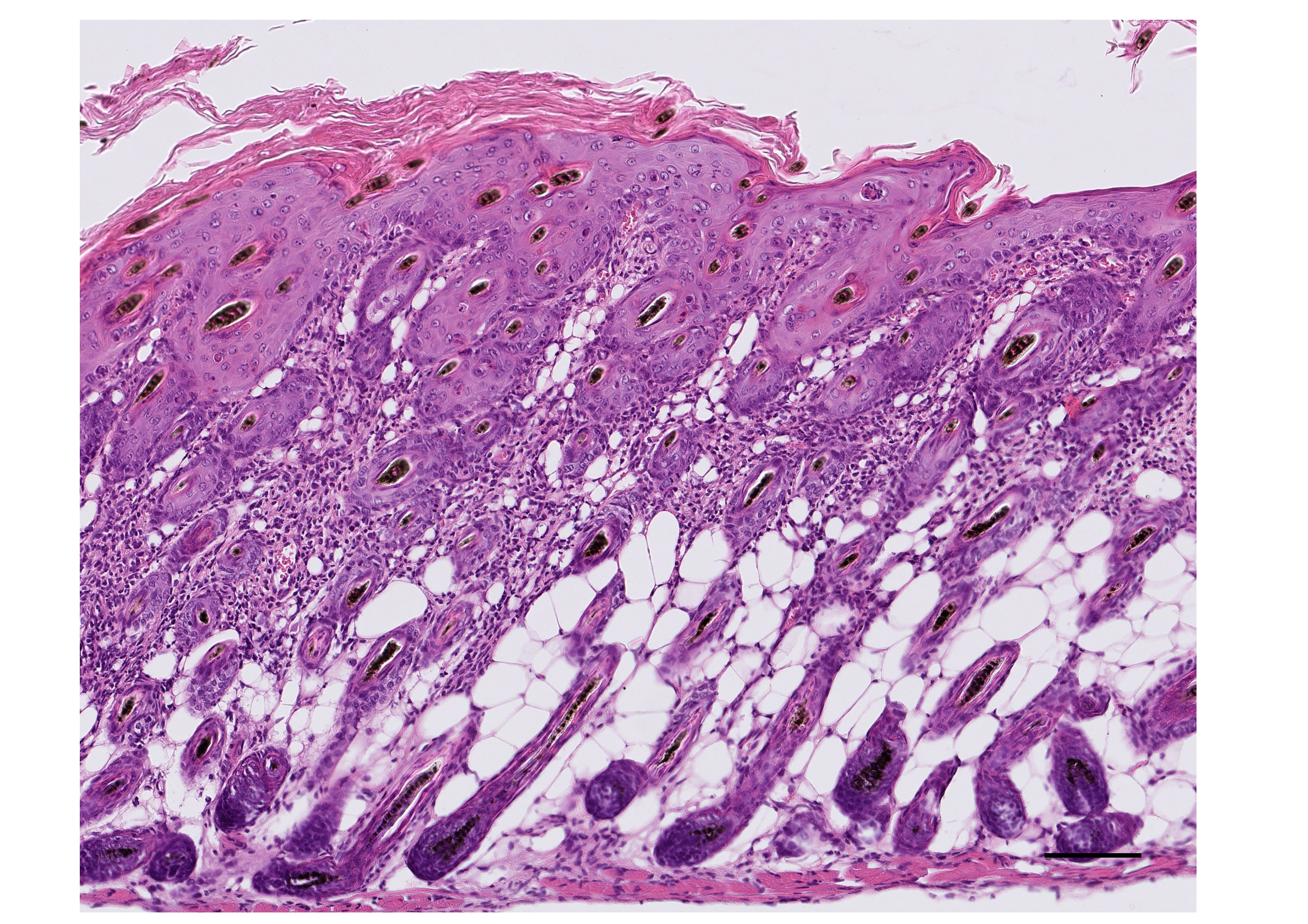

Supplement: Supplementary file 6 — Source data Fig. 4 [file 44318_2024_238_MOESM6_ESM.zip › Figure 4/4D/RIPK1 EKO; ZBP1 L_L HE copy.jpg]

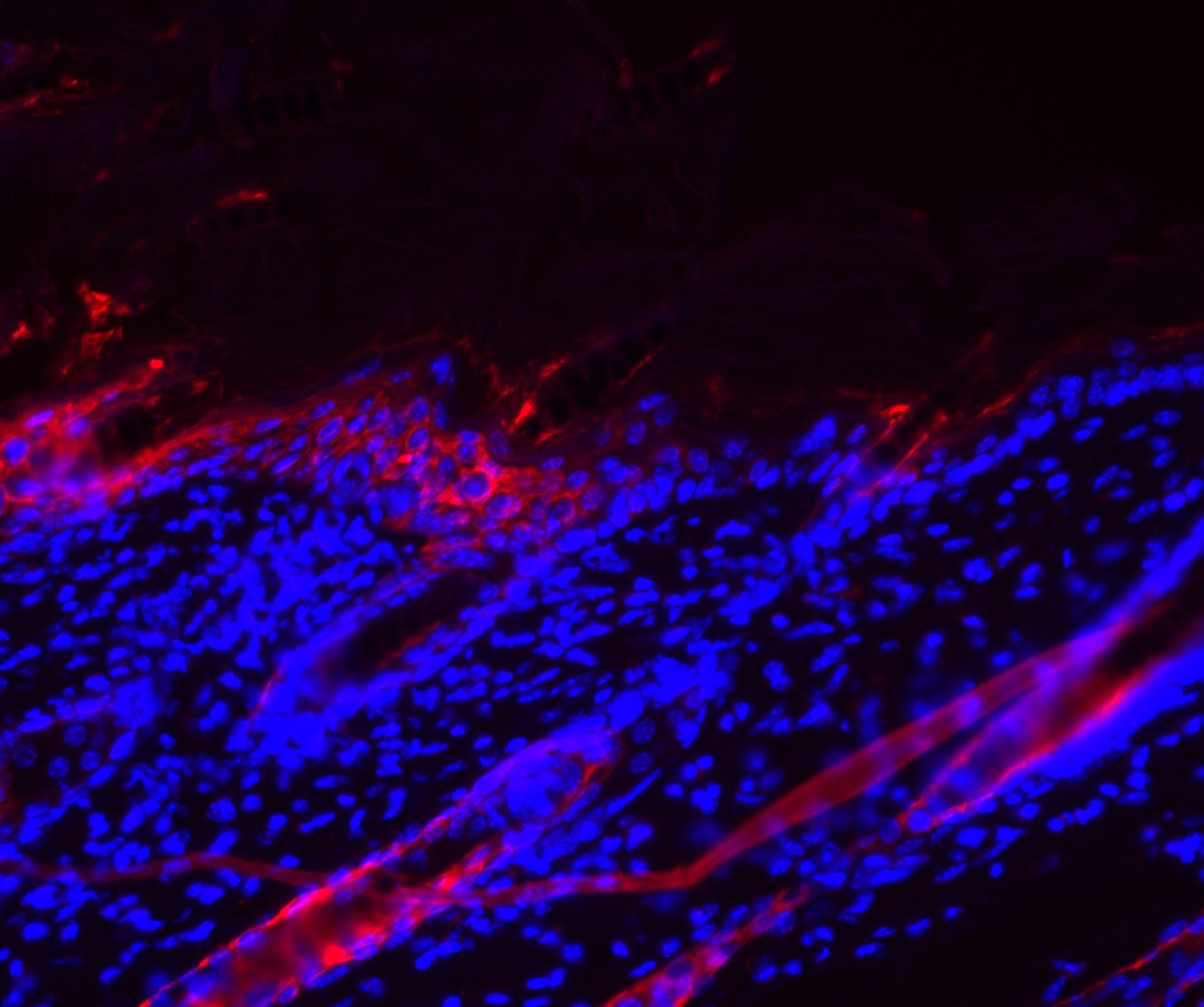

Supplement: Supplementary file 6 — Source data Fig. 4 [file 44318_2024_238_MOESM6_ESM.zip › Figure 4/4D/RIPK1 EKO; ZBP1 WT_WT K6_Hoechst copy.jpg]

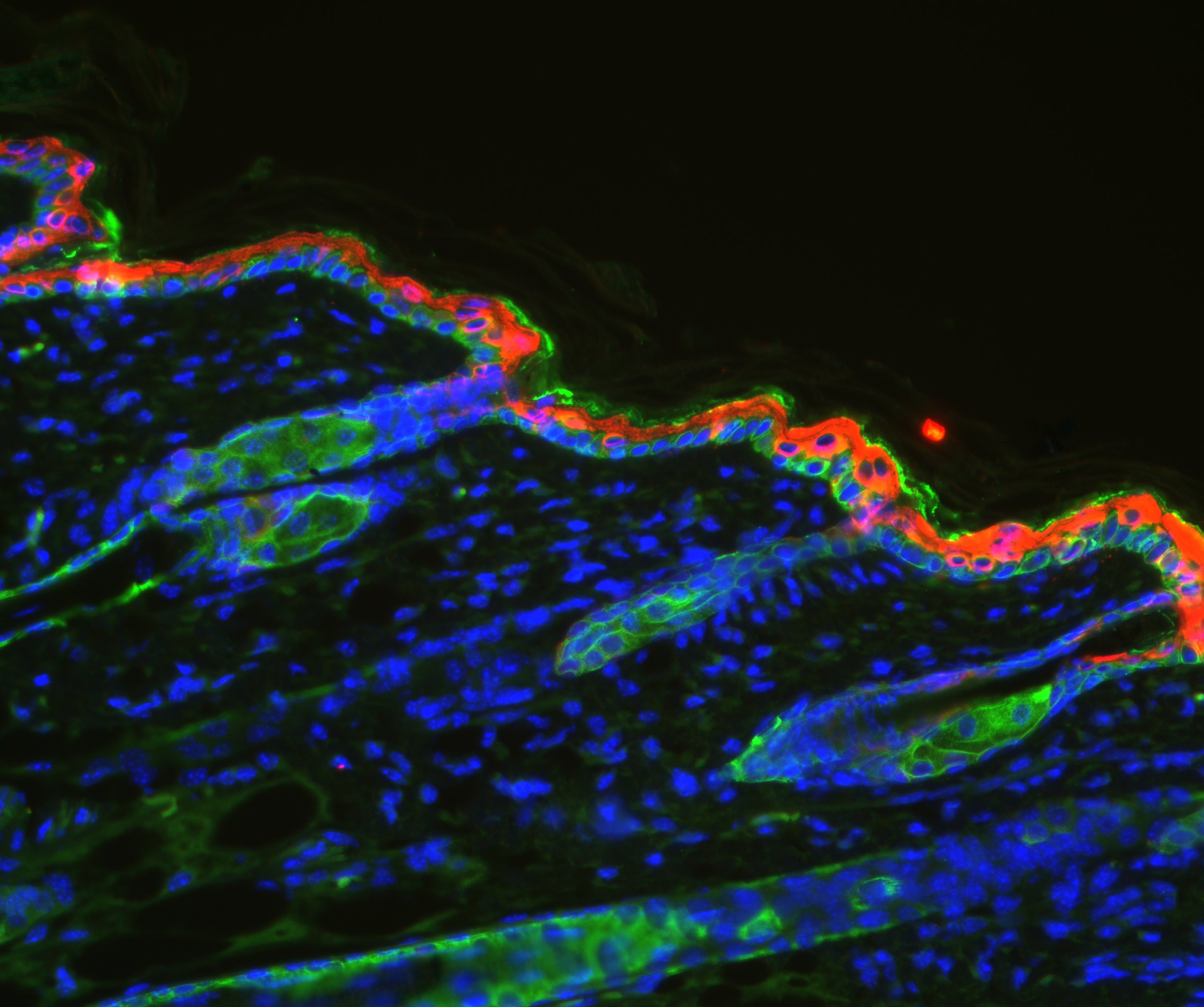

Supplement: Supplementary file 6 — Source data Fig. 4 [file 44318_2024_238_MOESM6_ESM.zip › Figure 4/4D/Control K10_K14_Hoechst copy.jpg]

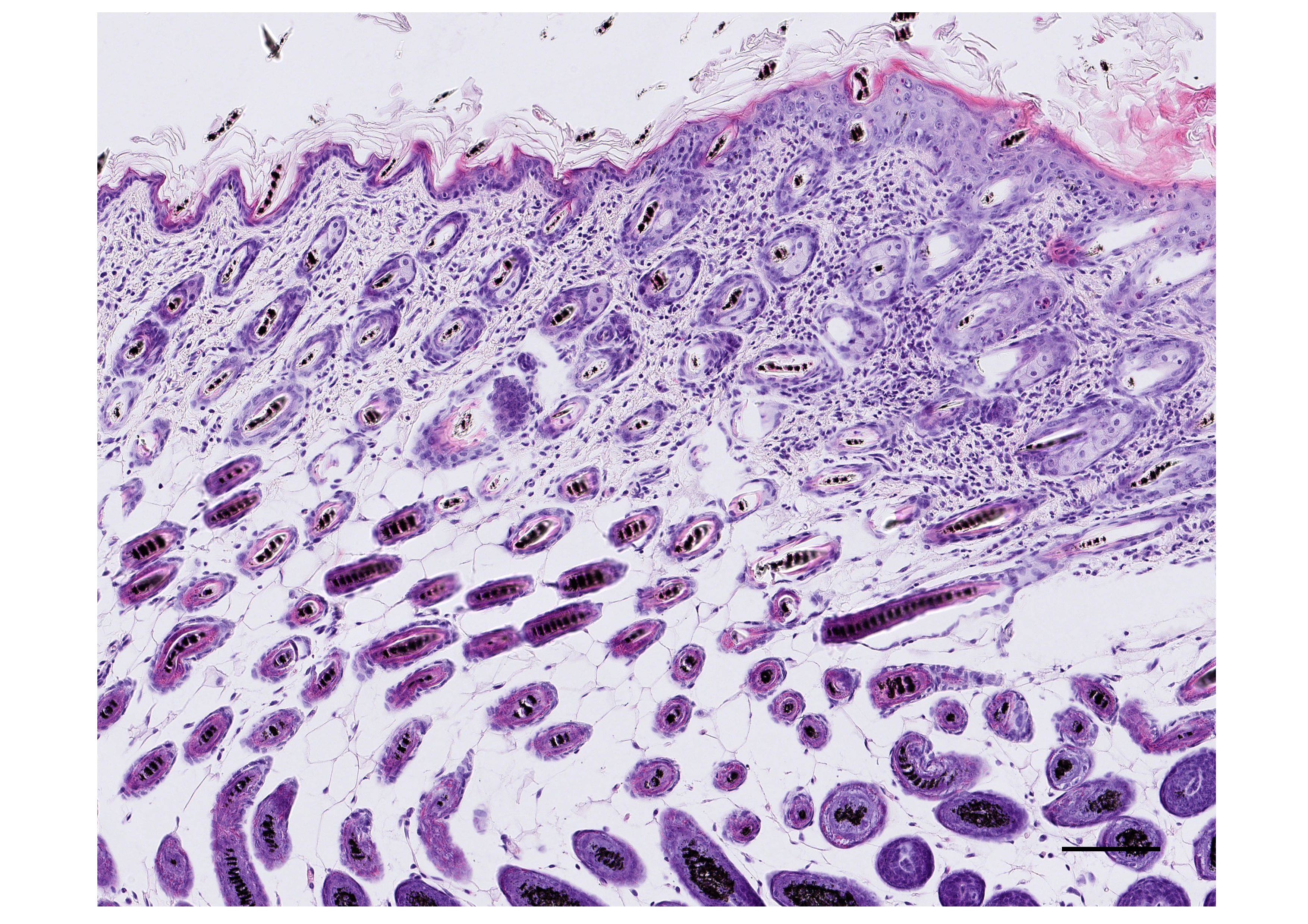

Supplement: Supplementary file 6 — Source data Fig. 4 [file 44318_2024_238_MOESM6_ESM.zip › Figure 4/4D/RIPK1 EKO; ZBP1 WT_WT HE copy.jpg]

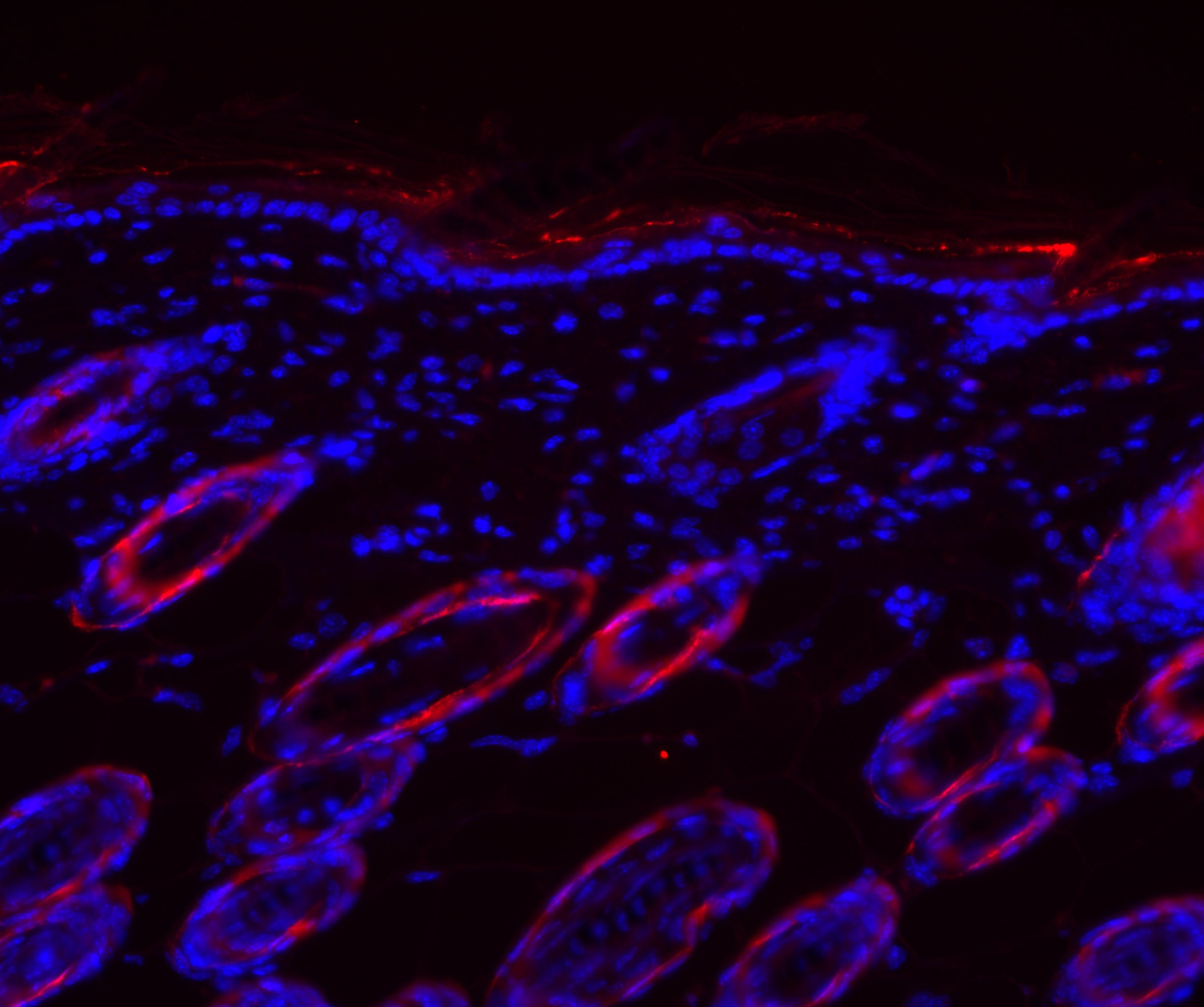

Supplement: Supplementary file 6 — Source data Fig. 4 [file 44318_2024_238_MOESM6_ESM.zip › Figure 4/4D/Control K6_Hoechst copy.jpg]

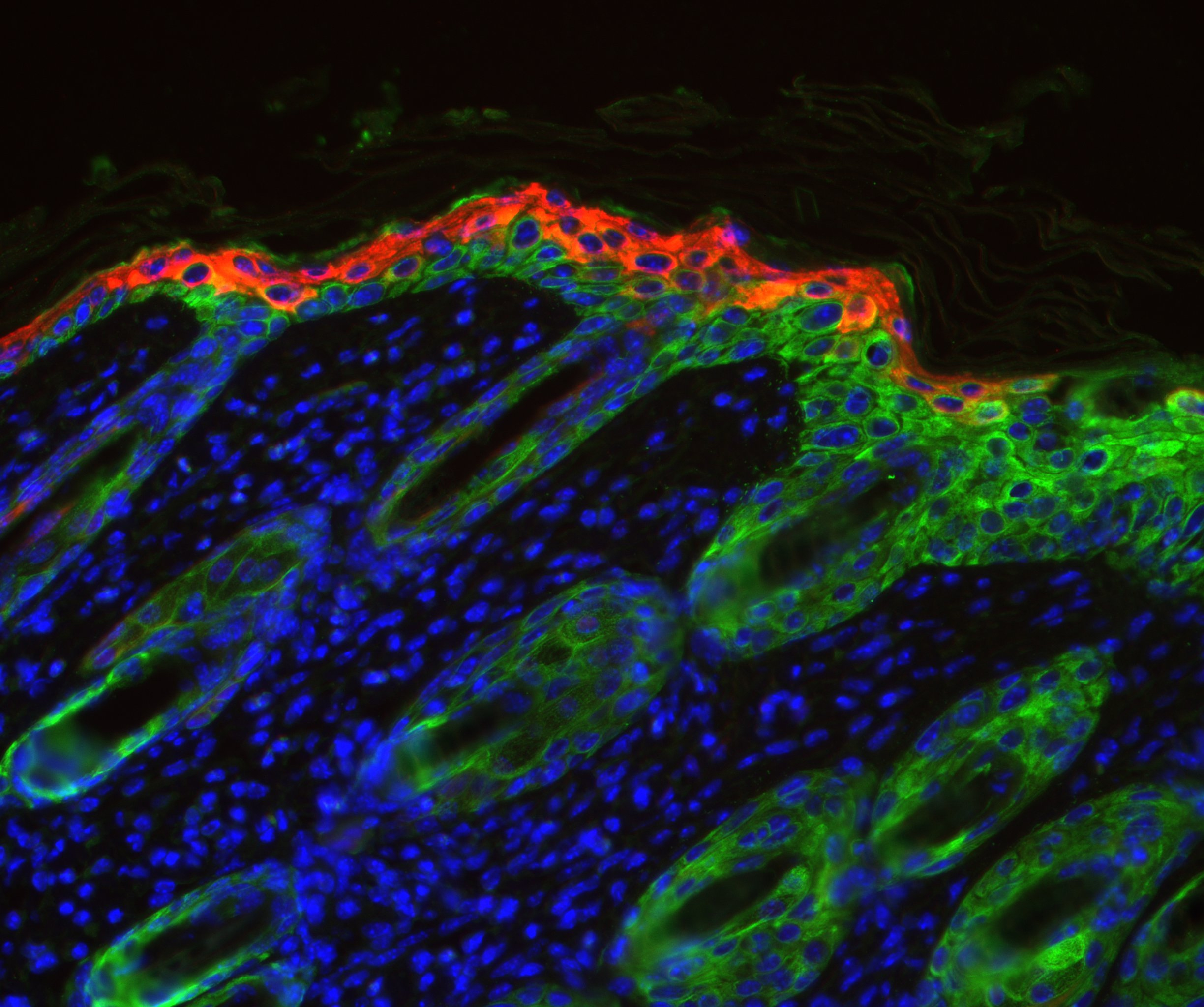

Supplement: Supplementary file 6 — Source data Fig. 4 [file 44318_2024_238_MOESM6_ESM.zip › Figure 4/4D/RIPK1 EKO; ZBP1 WT_WT K10_K14_Hoechst copy.jpg]

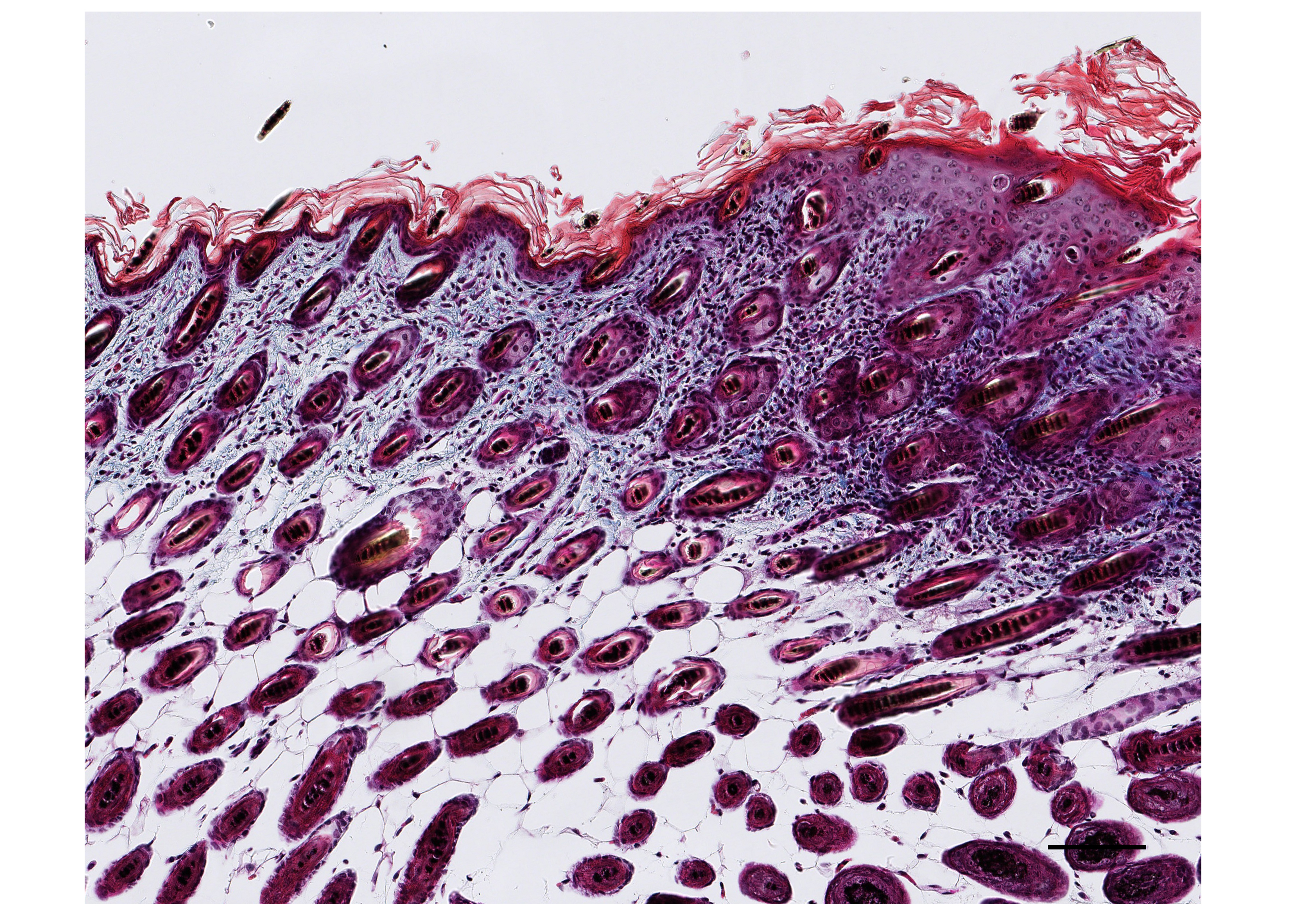

Supplement: Supplementary file 6 — Source data Fig. 4 [file 44318_2024_238_MOESM6_ESM.zip › Figure 4/4D/RIPK1 EKO; ZBP1 WT_WT Trichrome copy.jpg]

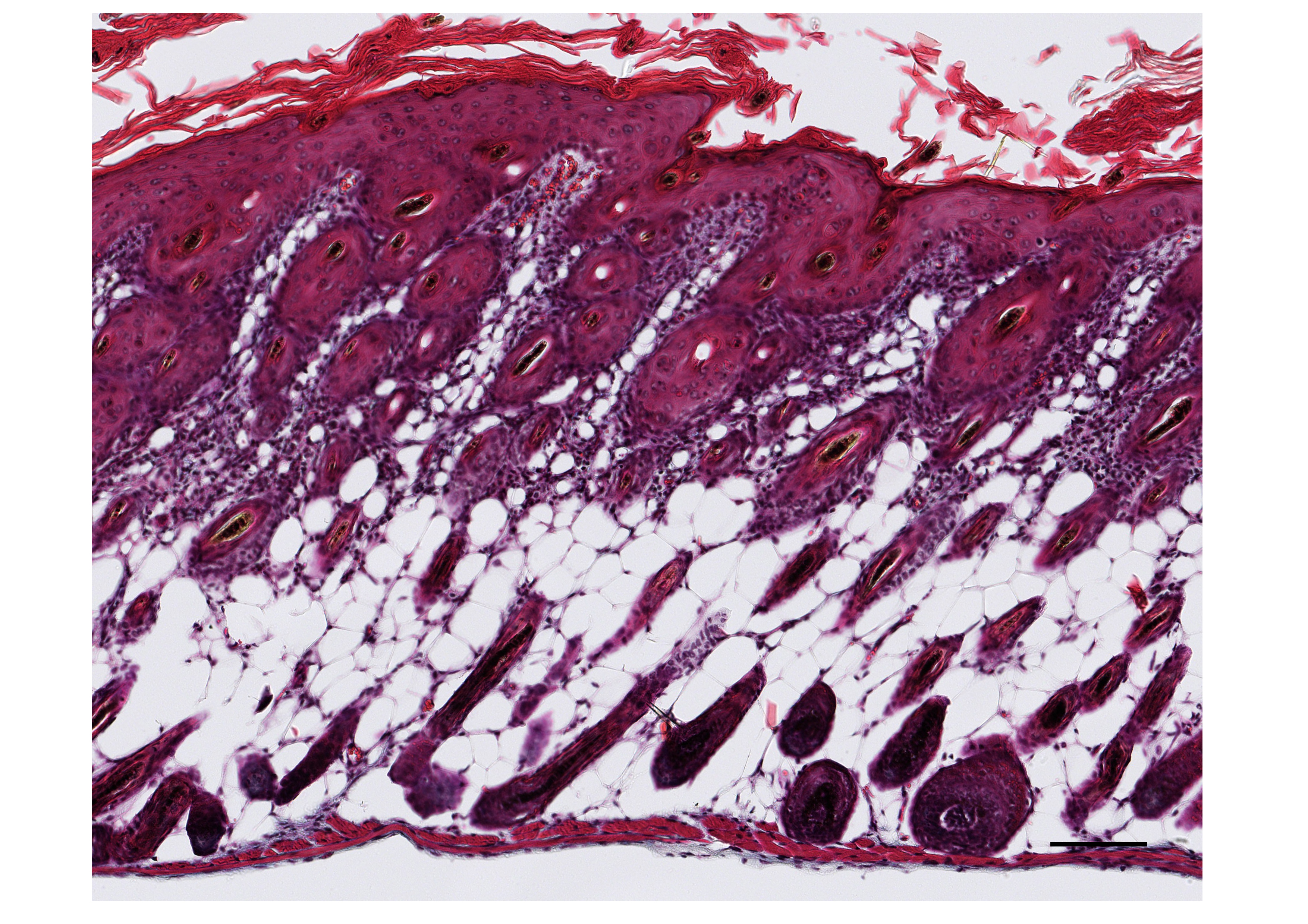

Supplement: Supplementary file 6 — Source data Fig. 4 [file 44318_2024_238_MOESM6_ESM.zip › Figure 4/4D/RIPK1 EKO; ZBP1 L_L Trichrome copy.jpg]

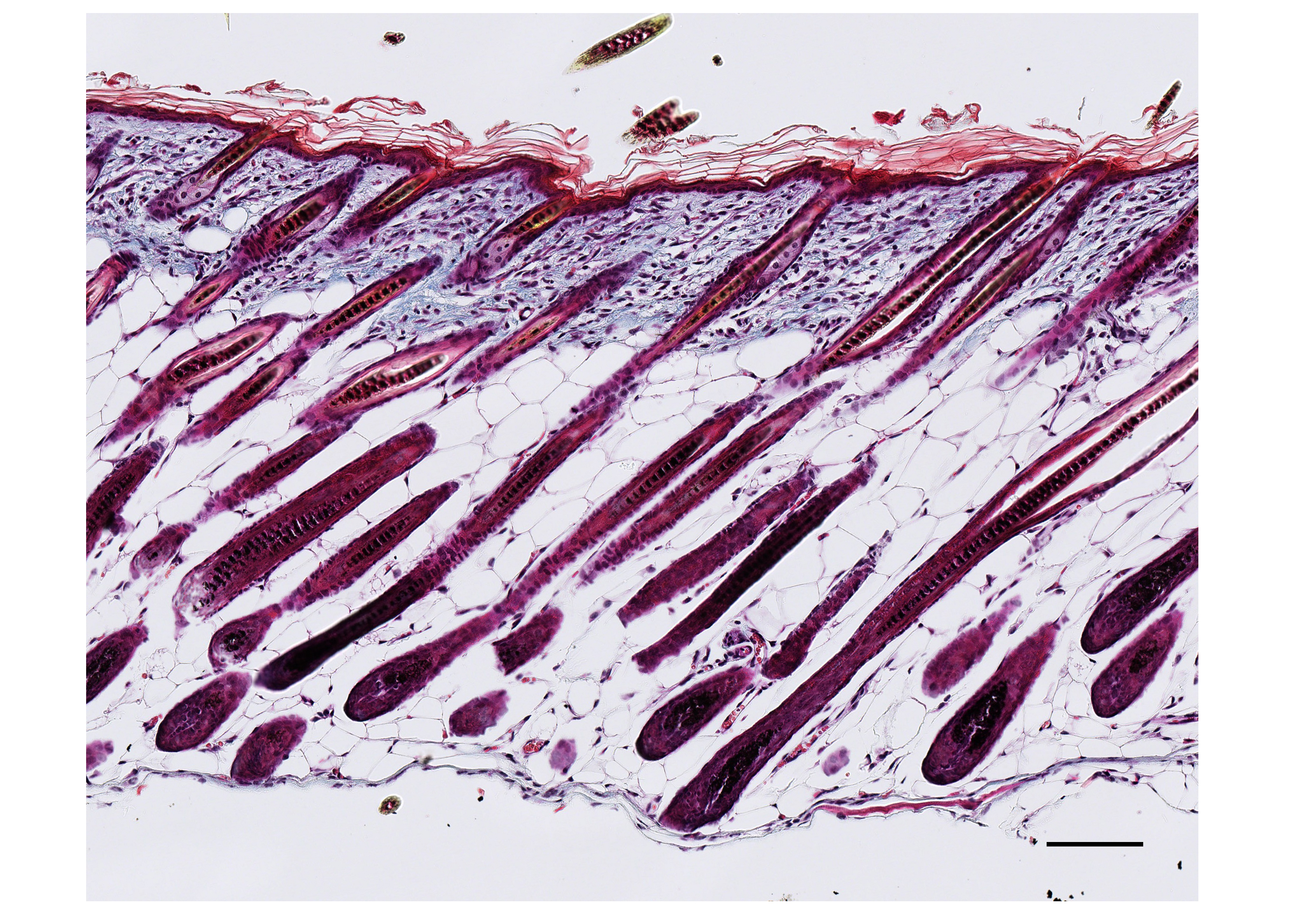

Supplement: Supplementary file 6 — Source data Fig. 4 [file 44318_2024_238_MOESM6_ESM.zip › Figure 4/4D/Control Trichrome copy.jpg]

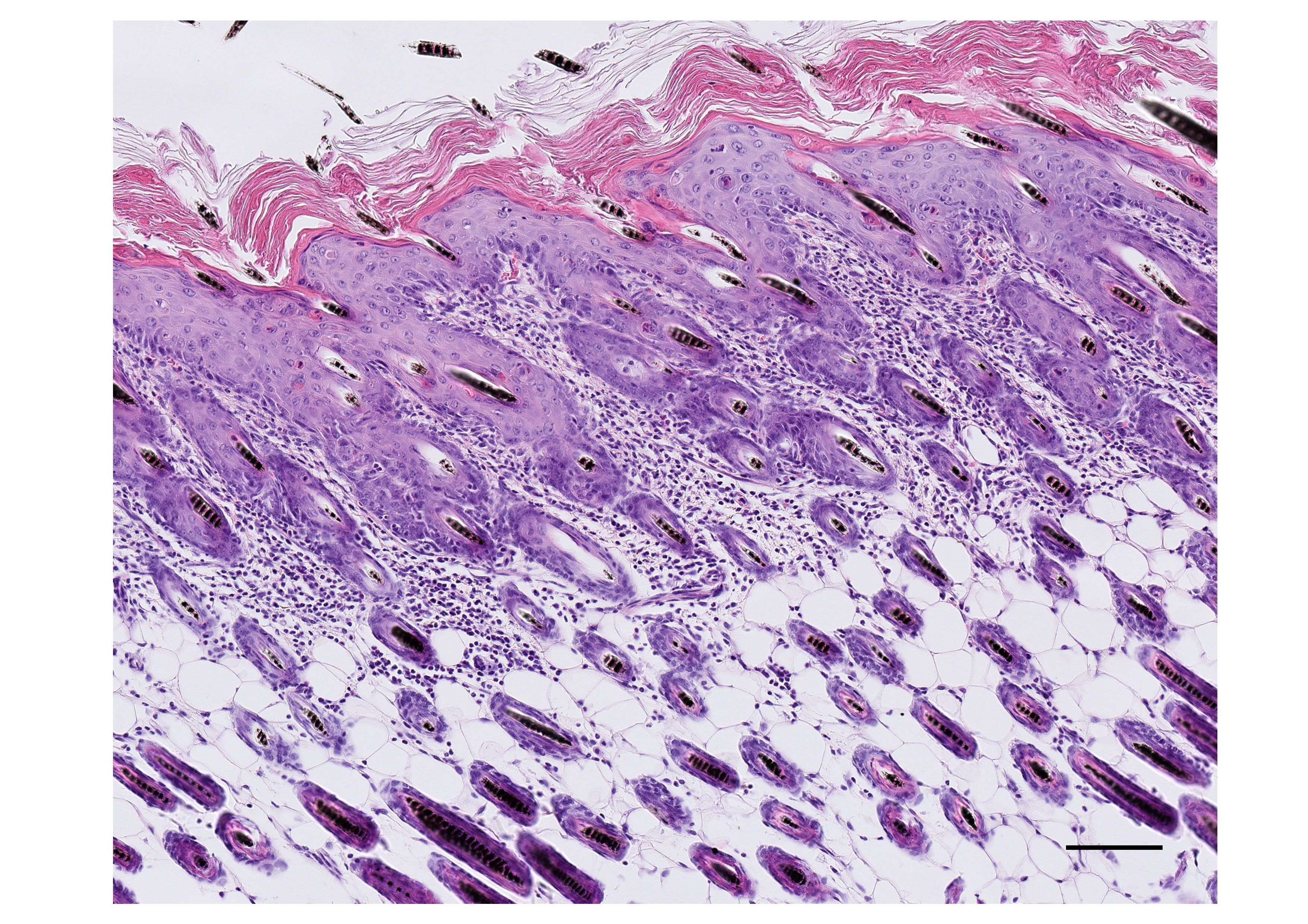

Supplement: Supplementary file 6 — Source data Fig. 4 [file 44318_2024_238_MOESM6_ESM.zip › Figure 4/4D/RIPK1 EKO; ZBP1 WT_L HE copy.jpg]

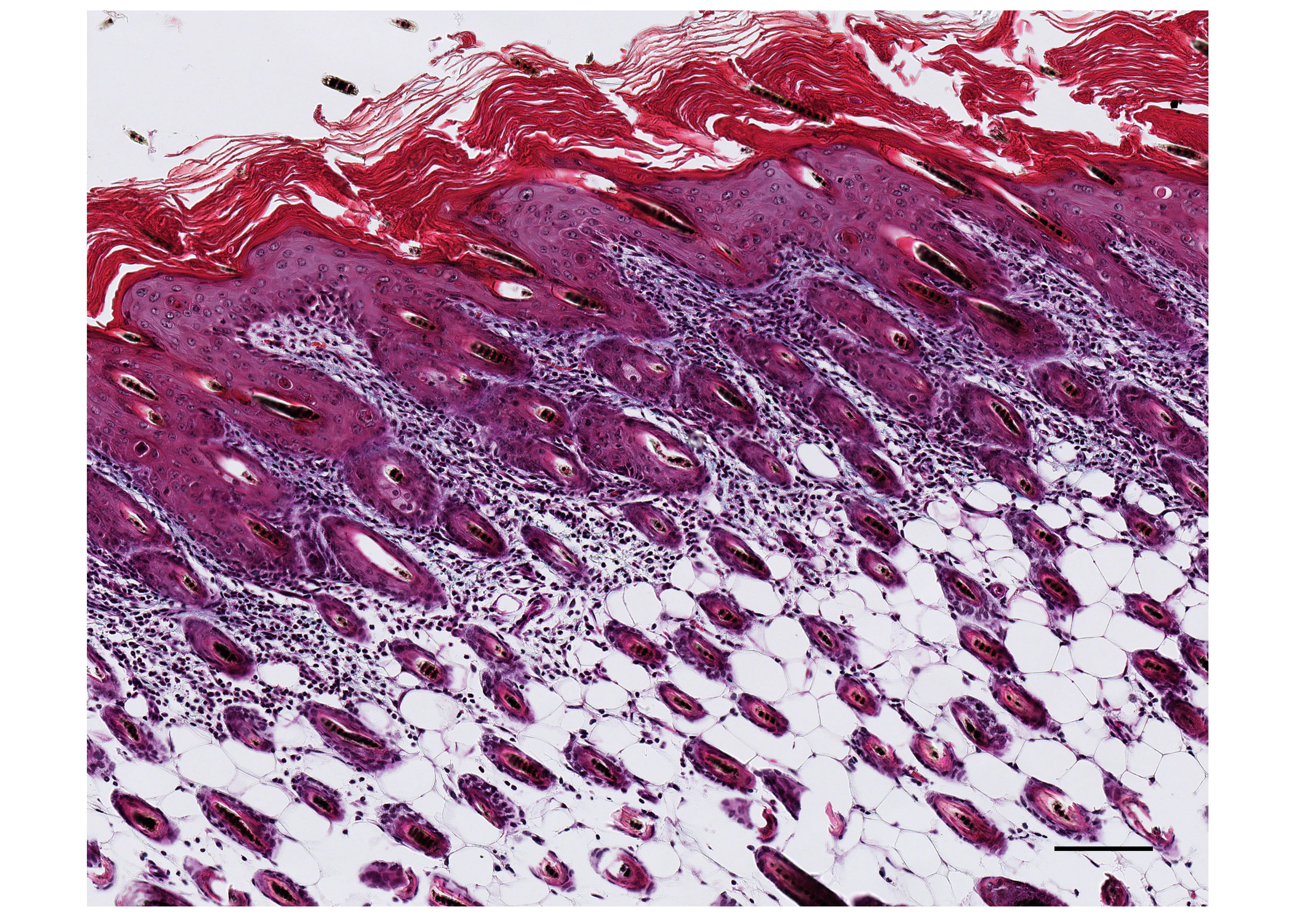

Supplement: Supplementary file 6 — Source data Fig. 4 [file 44318_2024_238_MOESM6_ESM.zip › Figure 4/4D/RIPK1 EKO; ZBP1 WT_L Trichrome copy.jpg]

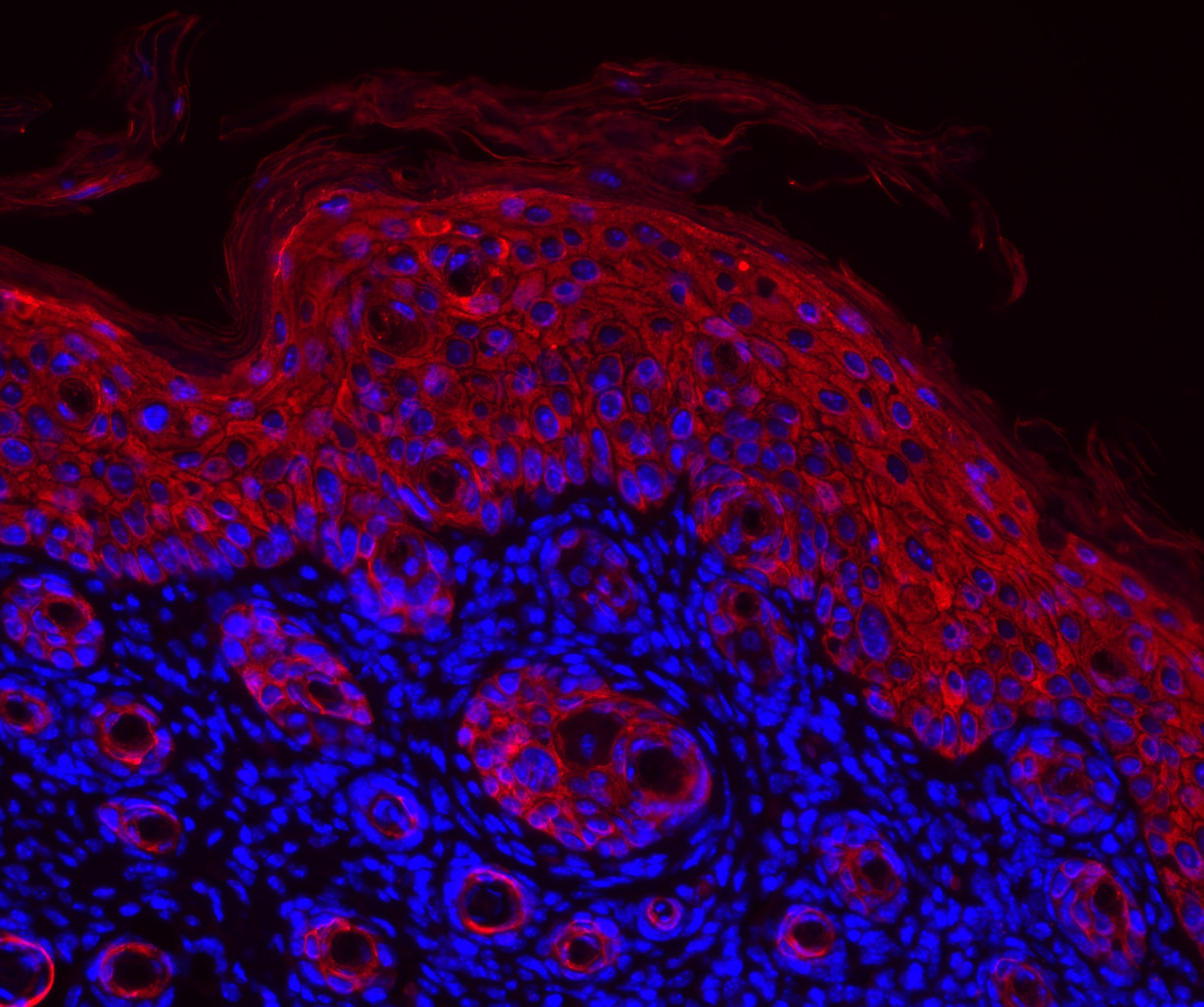

Supplement: Supplementary file 6 — Source data Fig. 4 [file 44318_2024_238_MOESM6_ESM.zip › Figure 4/4D/RIPK1 EKO; ZBP1 L_L K6_Hoechst copy.jpg]

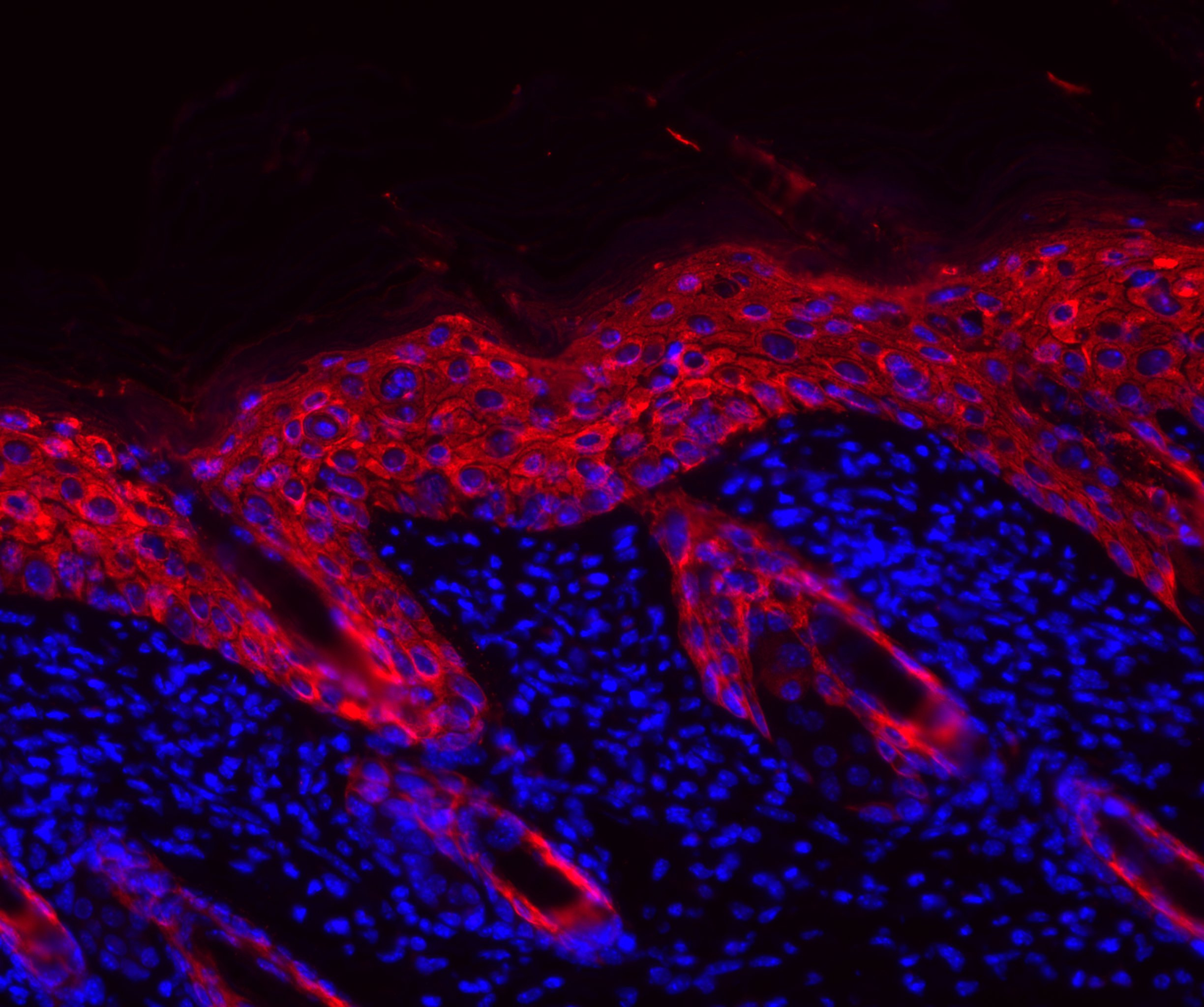

Supplement: Supplementary file 6 — Source data Fig. 4 [file 44318_2024_238_MOESM6_ESM.zip › Figure 4/4D/RIPK1 EKO; ZBP1 WT_L K6_Hoechst copy.jpg]

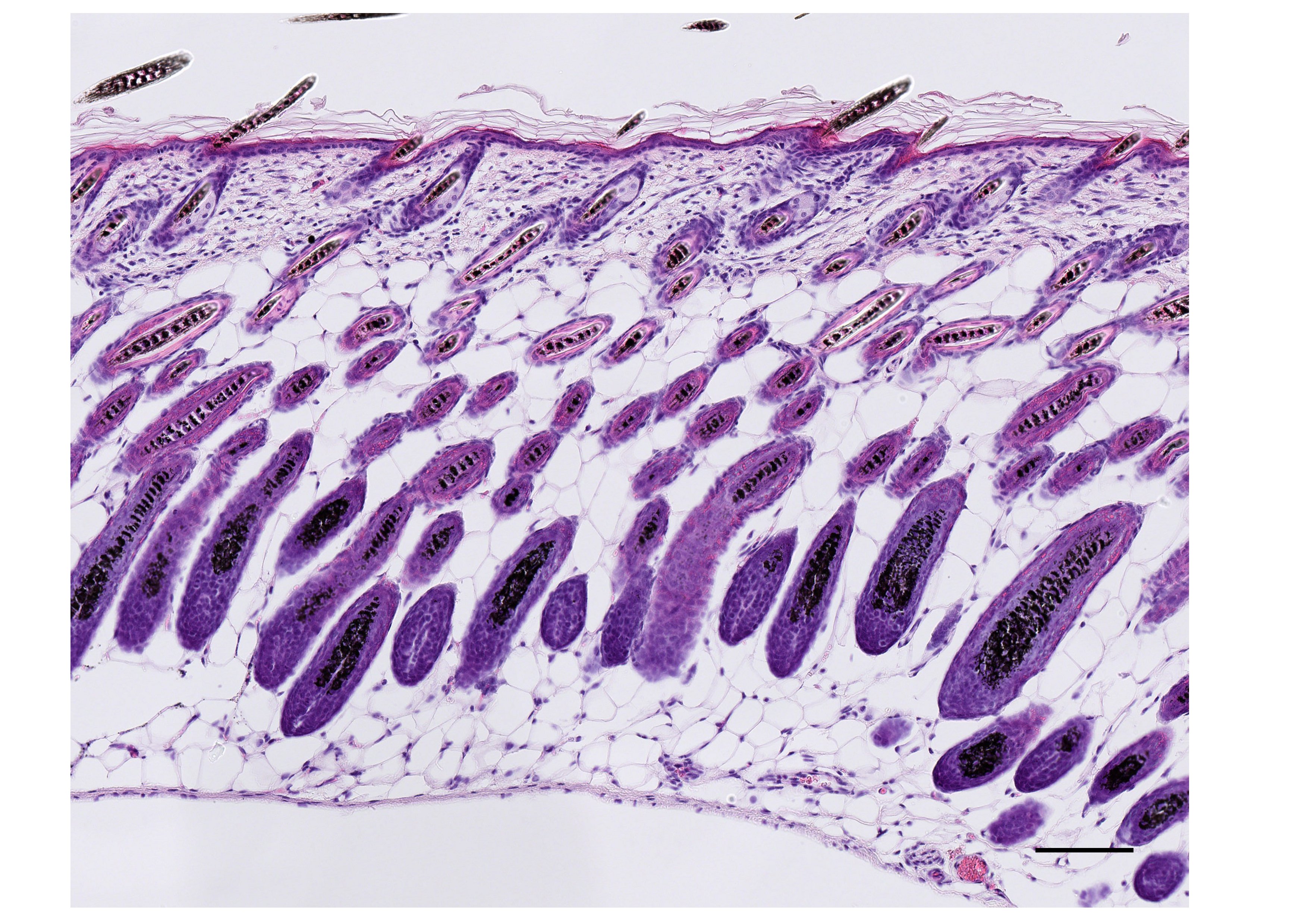

Supplement: Supplementary file 6 — Source data Fig. 4 [file 44318_2024_238_MOESM6_ESM.zip › Figure 4/4D/Control HE copy.jpg]

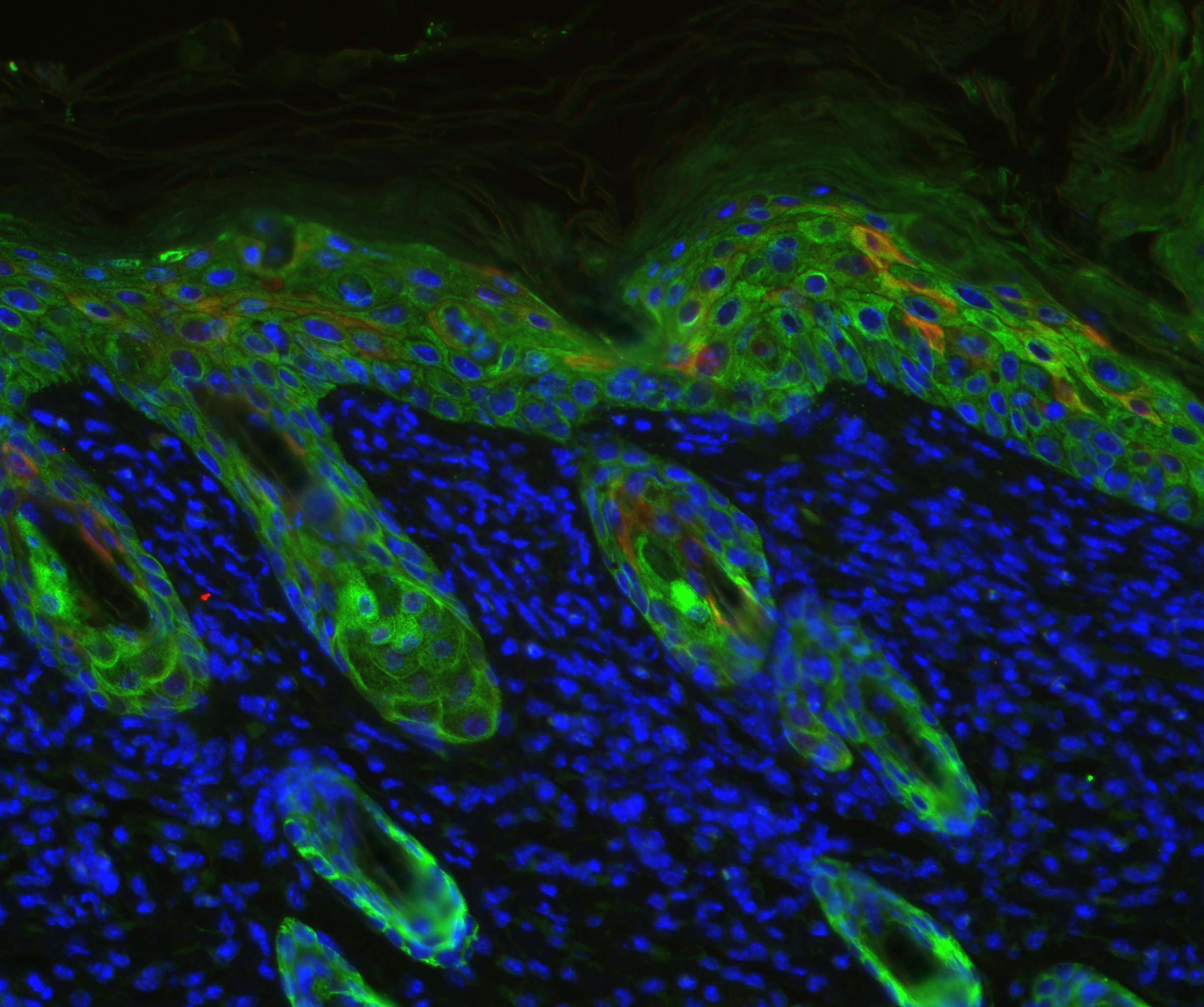

Supplement: Supplementary file 6 — Source data Fig. 4 [file 44318_2024_238_MOESM6_ESM.zip › Figure 4/4D/RIPK1 EKO; ZBP1 WT_L K10_K14_Hoechst copy.jpg]

## Slide 1
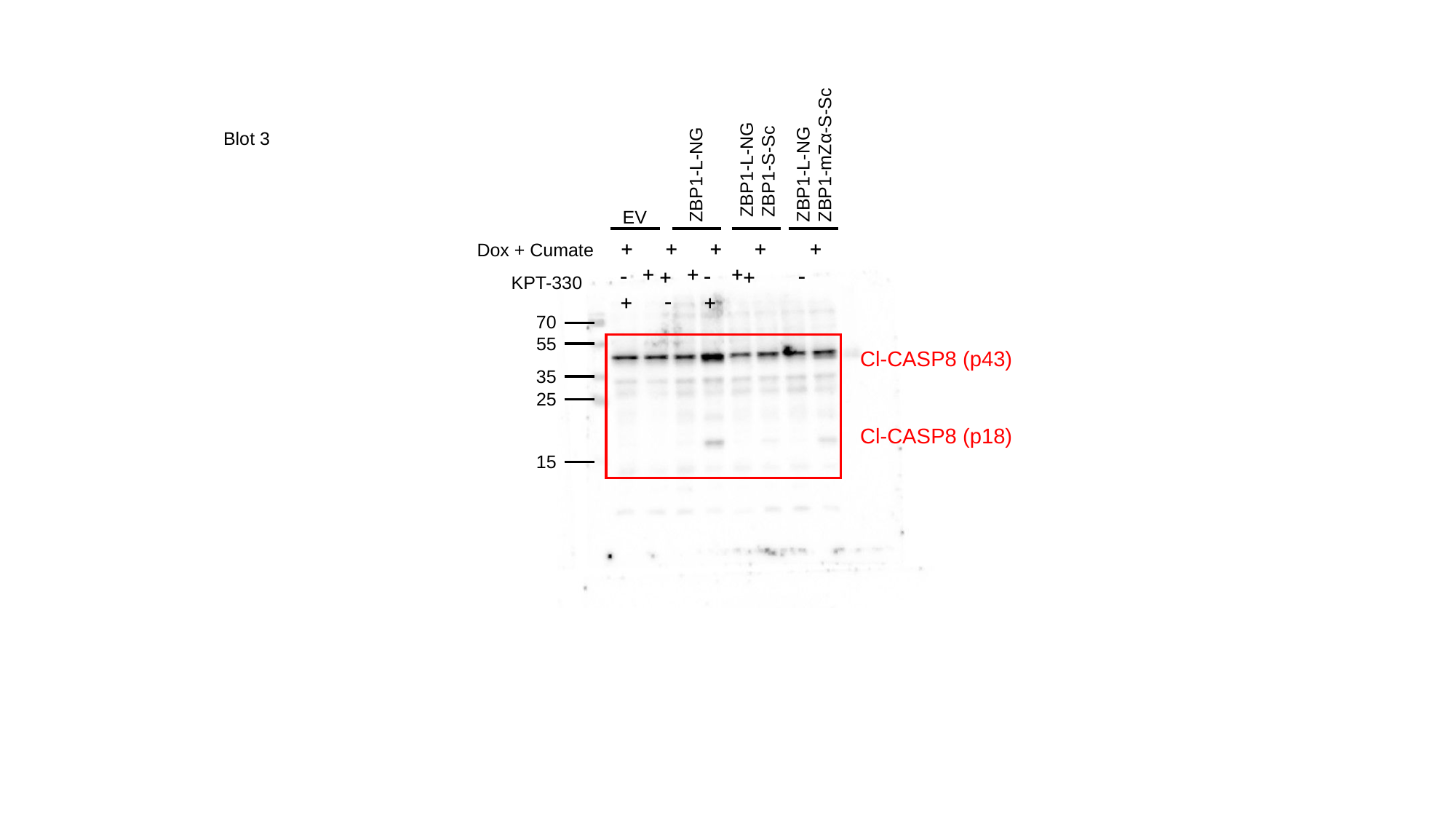

Blot 3
ZBP1-L-NG
ZBP1-mZα-S-Sc
ZBP1-L-NG
ZBP1-S-Sc
ZBP1-L-NG
EV
+ + + + + + + +
Dox + Cumate
- + - + - + - +
KPT-330
70
55
Cl-CASP8 (p43)
35
25
Cl-CASP8 (p18)
15

Supplement: Supplementary file 7 — Source data Fig. 5 [file 44318_2024_238_MOESM7_ESM.zip › Figure 5/5F/western Cl-CASP8.pptx]

## Slide 1
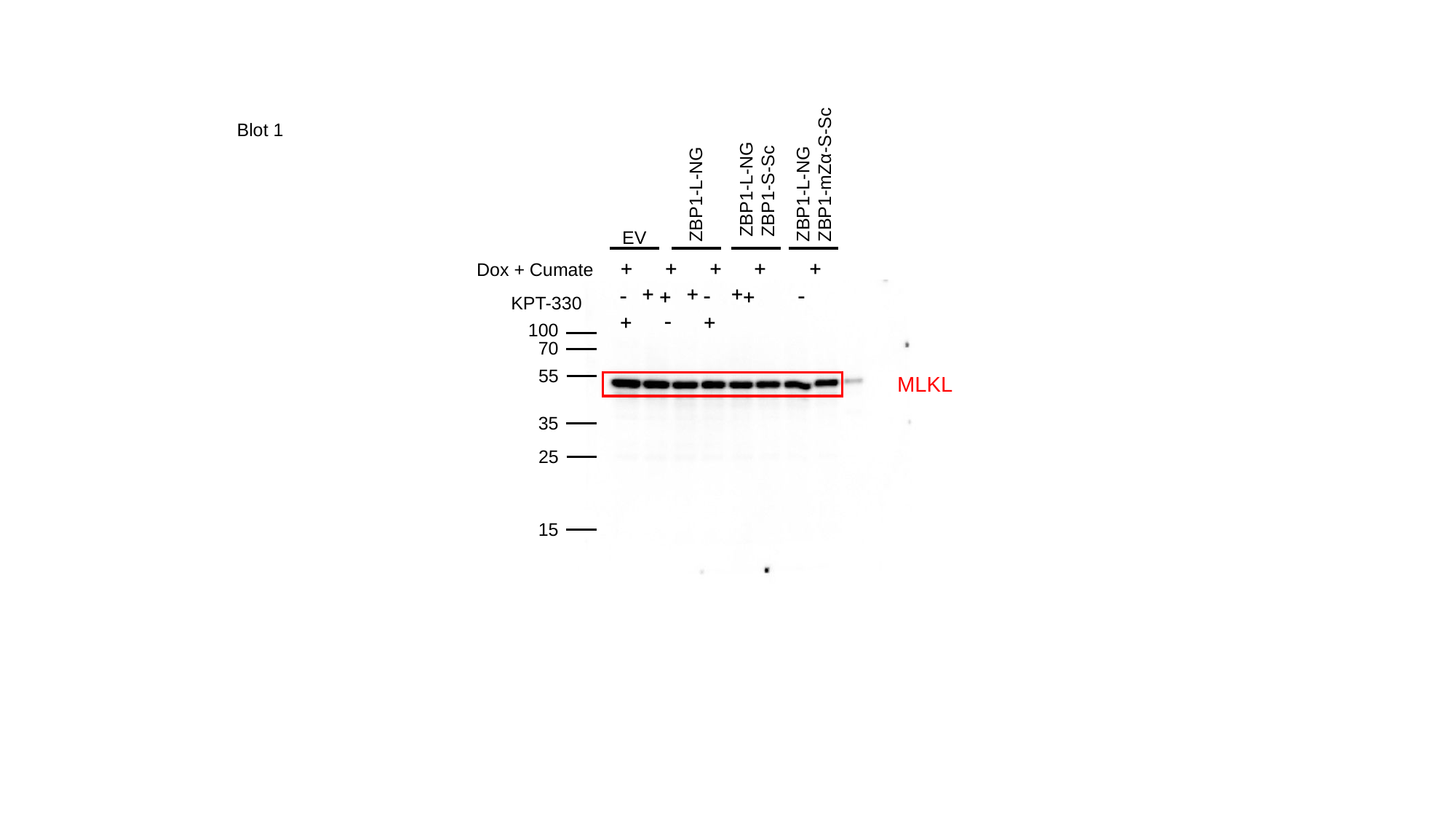

Blot 1
ZBP1-L-NG
ZBP1-mZα-S-Sc
ZBP1-L-NG
ZBP1-S-Sc
ZBP1-L-NG
EV
+ + + + + + + +
Dox + Cumate
- + - + - + - +
KPT-330
100
70
55
MLKL
35
25
15

Supplement: Supplementary file 7 — Source data Fig. 5 [file 44318_2024_238_MOESM7_ESM.zip › Figure 5/5F/western MLKL.pptx]

## Slide 1
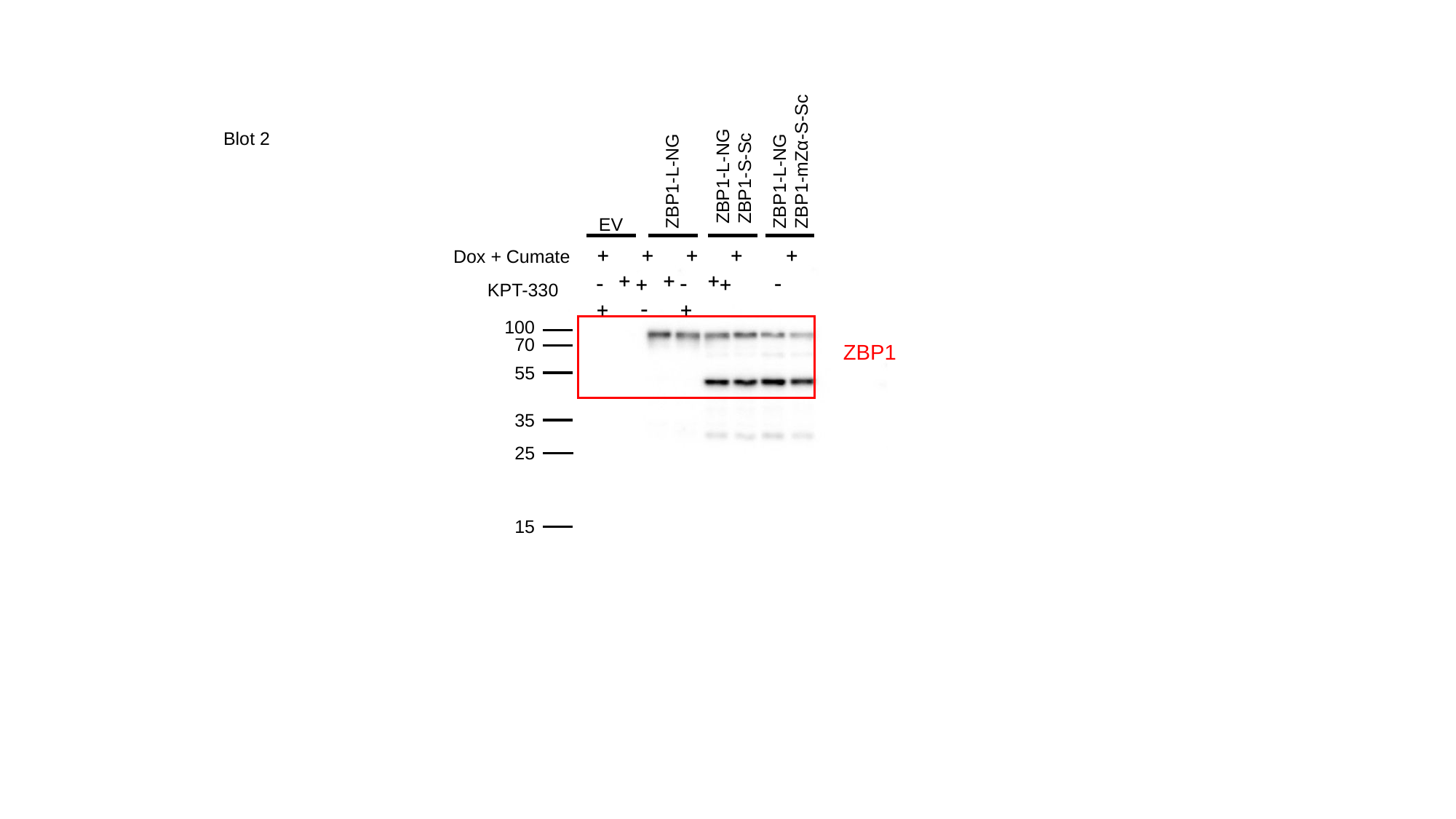

Blot 2
ZBP1-L-NG
ZBP1-mZα-S-Sc
ZBP1-L-NG
ZBP1-S-Sc
ZBP1-L-NG
EV
+ + + + + + + +
Dox + Cumate
- + - + - + - +
KPT-330
100
70
ZBP1
55
35
25
15

Supplement: Supplementary file 7 — Source data Fig. 5 [file 44318_2024_238_MOESM7_ESM.zip › Figure 5/5F/western ZBP1.pptx]

## Slide 1
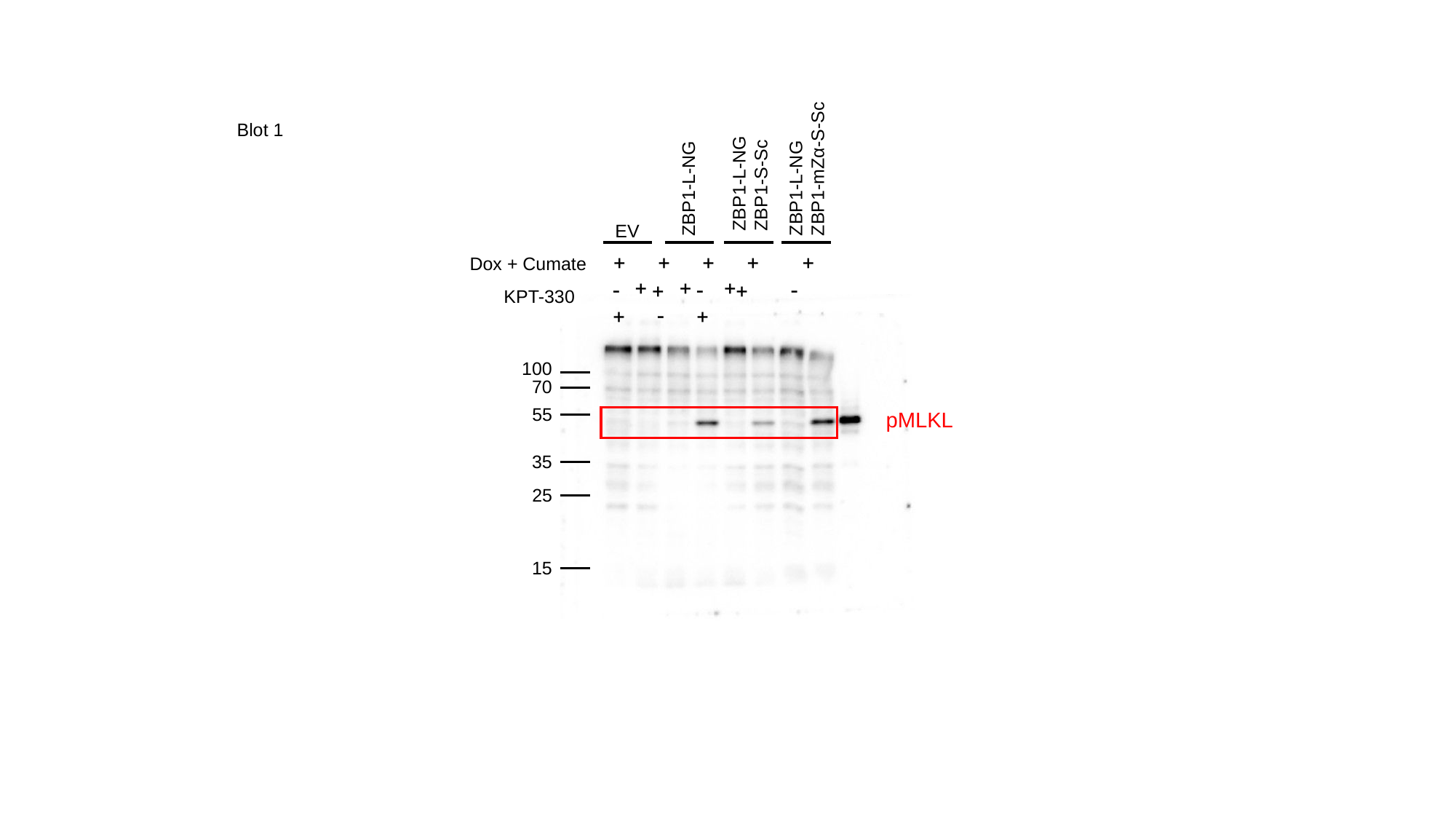

Blot 1
ZBP1-L-NG
ZBP1-mZα-S-Sc
ZBP1-L-NG
ZBP1-S-Sc
ZBP1-L-NG
EV
+ + + + + + + +
Dox + Cumate
- + - + - + - +
KPT-330
100
70
55
pMLKL
35
25
15

Supplement: Supplementary file 7 — Source data Fig. 5 [file 44318_2024_238_MOESM7_ESM.zip › Figure 5/5F/western pMLKL.pptx]

## Slide 1
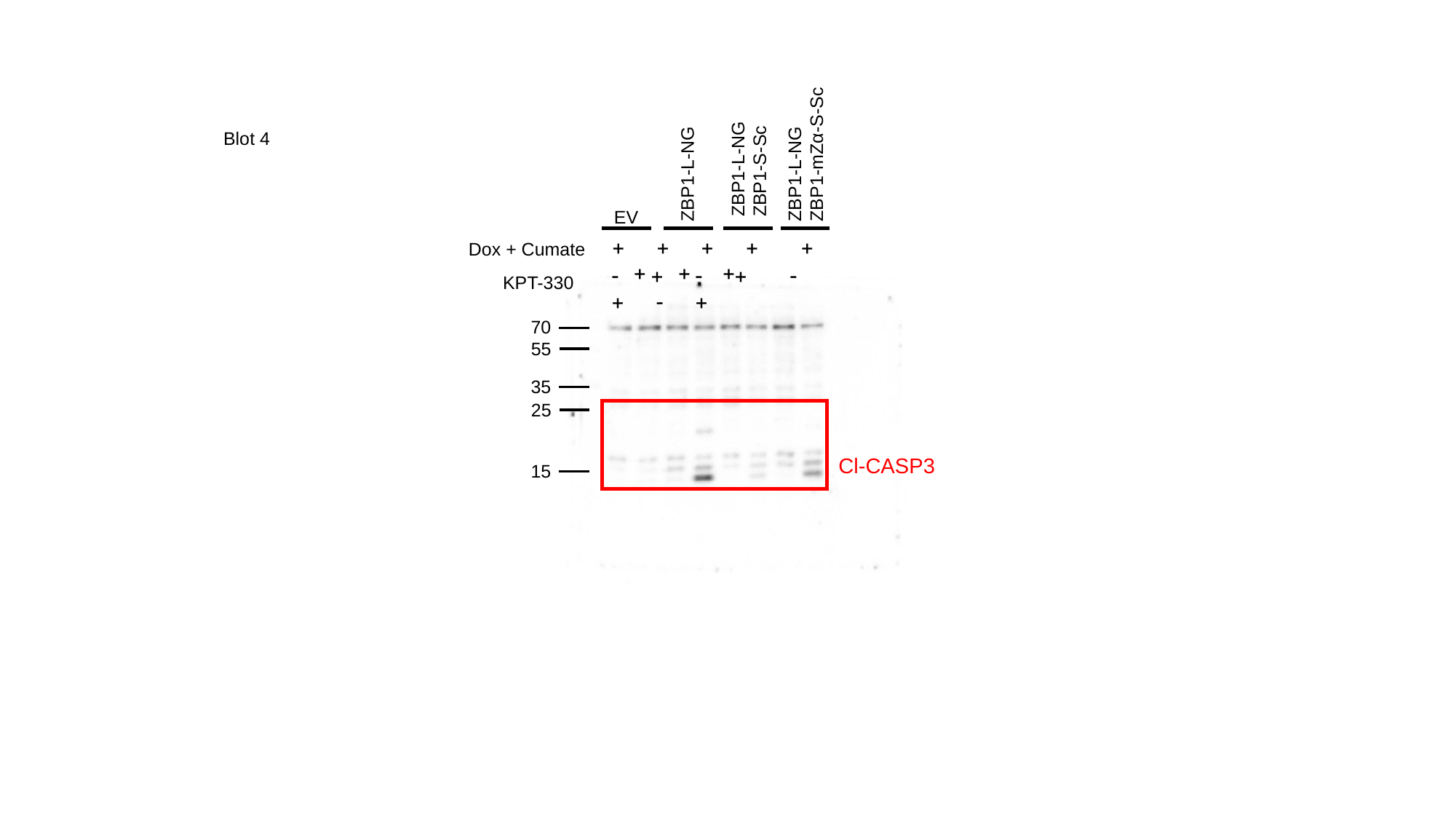

Blot 4
ZBP1-L-NG
ZBP1-mZα-S-Sc
ZBP1-L-NG
ZBP1-S-Sc
ZBP1-L-NG
EV
+ + + + + + + +
Dox + Cumate
- + - + - + - +
KPT-330
70
55
35
25
Cl-CASP3
15

Supplement: Supplementary file 7 — Source data Fig. 5 [file 44318_2024_238_MOESM7_ESM.zip › Figure 5/5F/western Cl-CASP3.pptx]

## Slide 1
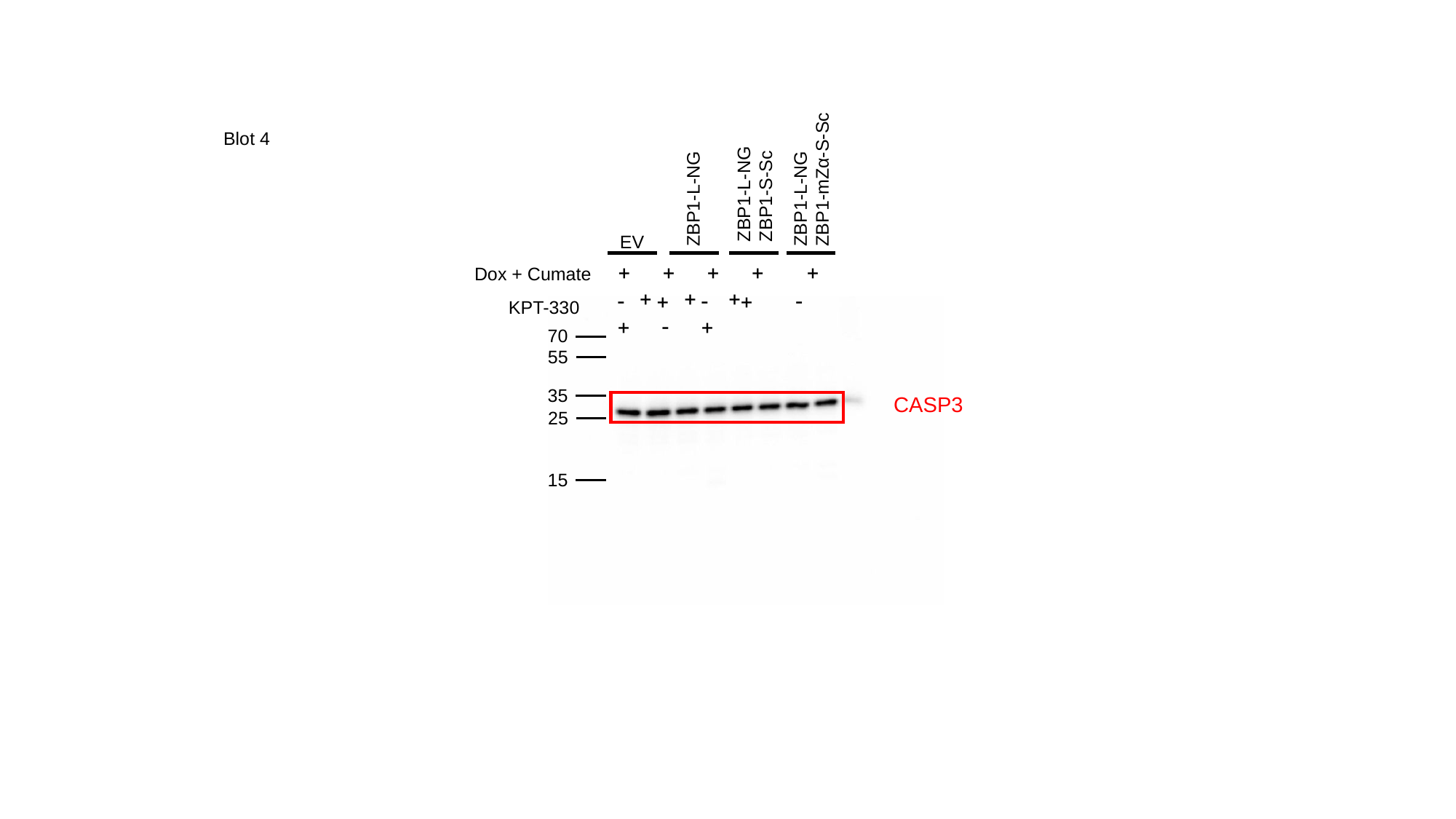

Blot 4
ZBP1-L-NG
ZBP1-mZα-S-Sc
ZBP1-L-NG
ZBP1-S-Sc
ZBP1-L-NG
EV
+ + + + + + + +
Dox + Cumate
- + - + - + - +
KPT-330
70
55
35
CASP3
25
15

Supplement: Supplementary file 7 — Source data Fig. 5 [file 44318_2024_238_MOESM7_ESM.zip › Figure 5/5F/western CASP3.pptx]

## Slide 1
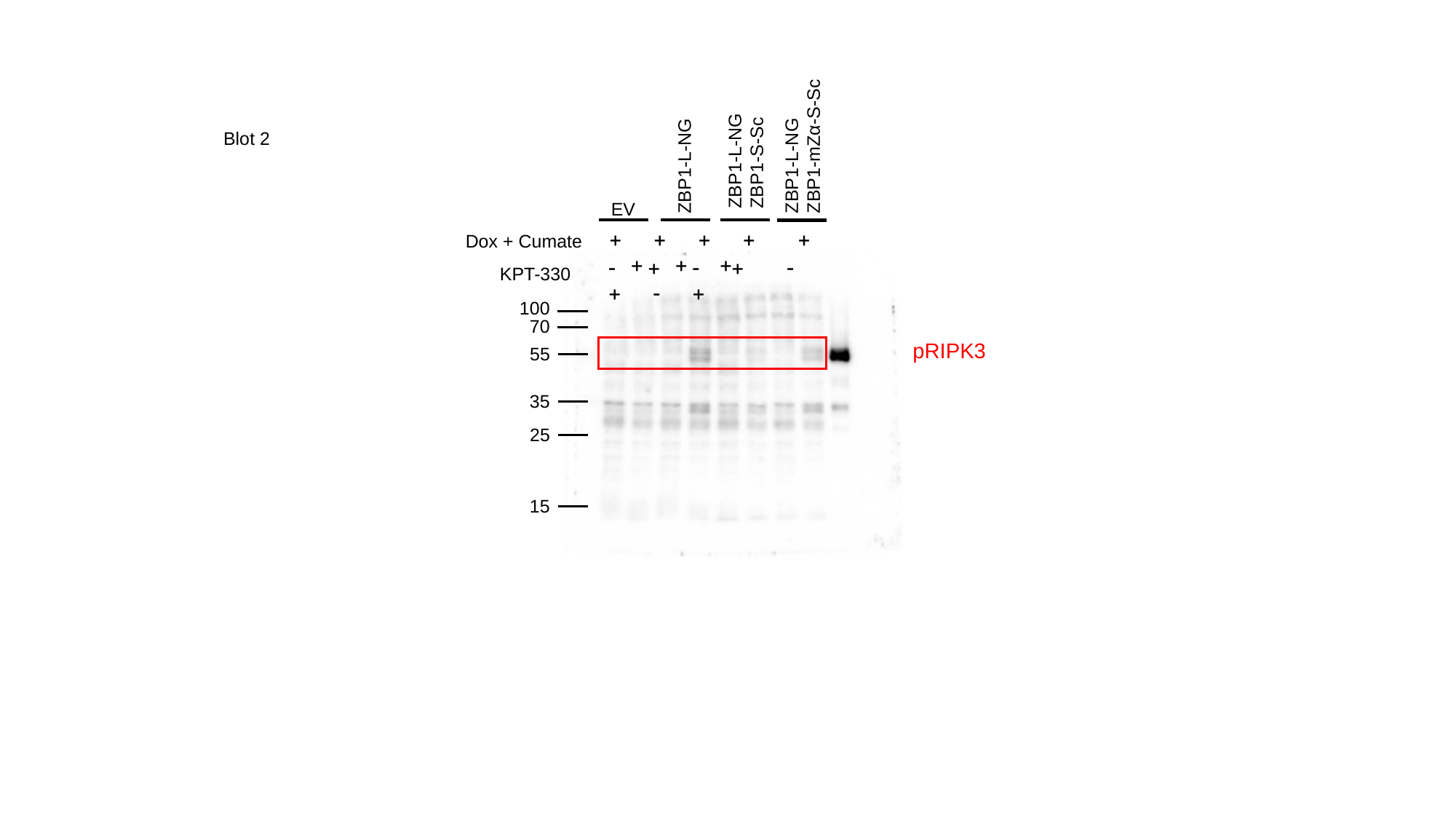

ZBP1-L-NG
ZBP1-mZα-S-Sc
Blot 2
ZBP1-L-NG
ZBP1-S-Sc
ZBP1-L-NG
EV
+ + + + + + + +
Dox + Cumate
- + - + - + - +
KPT-330
100
70
pRIPK3
55
35
25
15

Supplement: Supplementary file 7 — Source data Fig. 5 [file 44318_2024_238_MOESM7_ESM.zip › Figure 5/5F/western pRIPK3.pptx]

## Slide 1
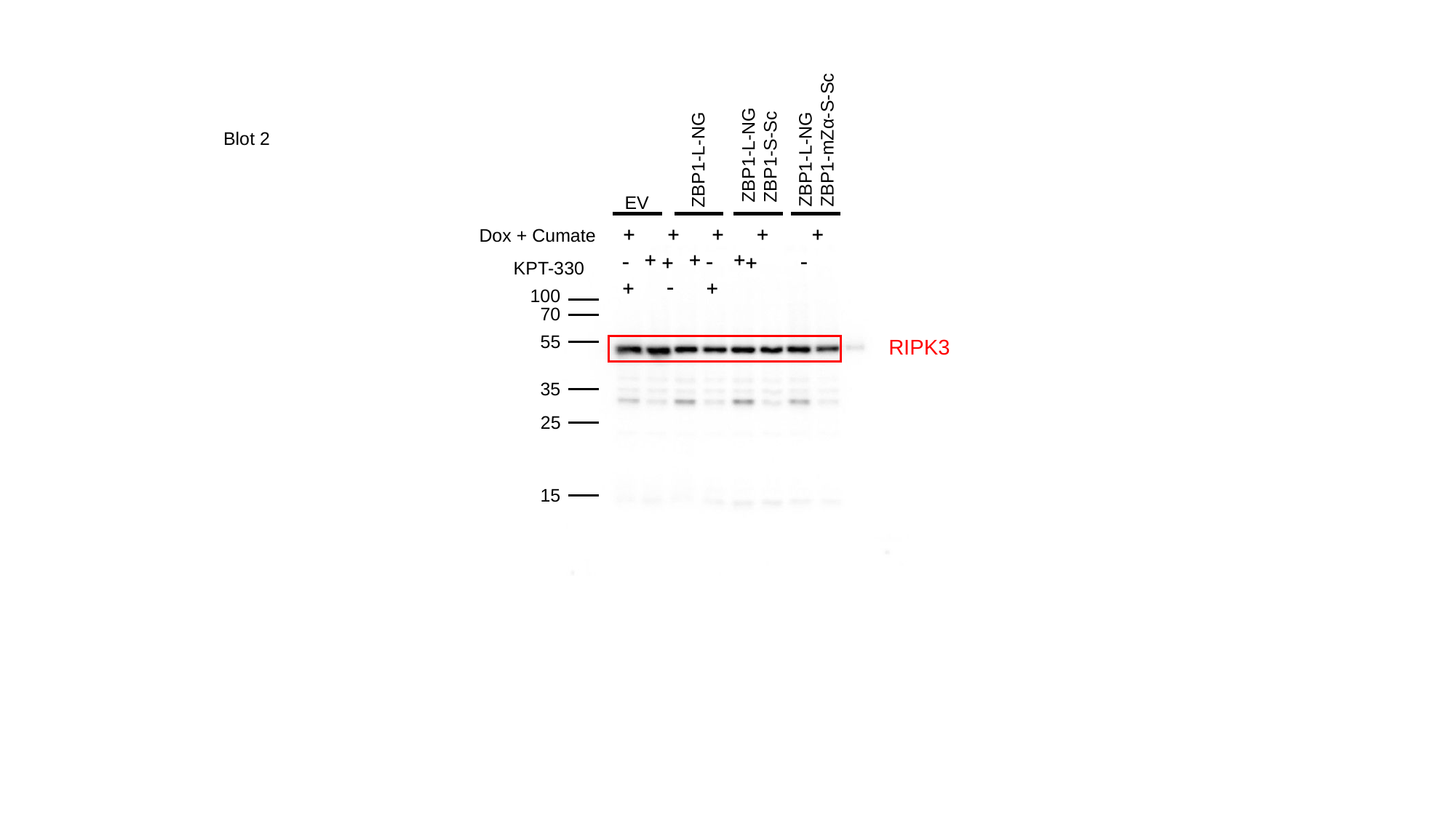

ZBP1-L-NG
ZBP1-mZα-S-Sc
Blot 2
ZBP1-L-NG
ZBP1-S-Sc
ZBP1-L-NG
EV
+ + + + + + + +
Dox + Cumate
- + - + - + - +
KPT-330
100
70
55
RIPK3
35
25
15

Supplement: Supplementary file 7 — Source data Fig. 5 [file 44318_2024_238_MOESM7_ESM.zip › Figure 5/5F/western RIPK3.pptx]

## Slide 1
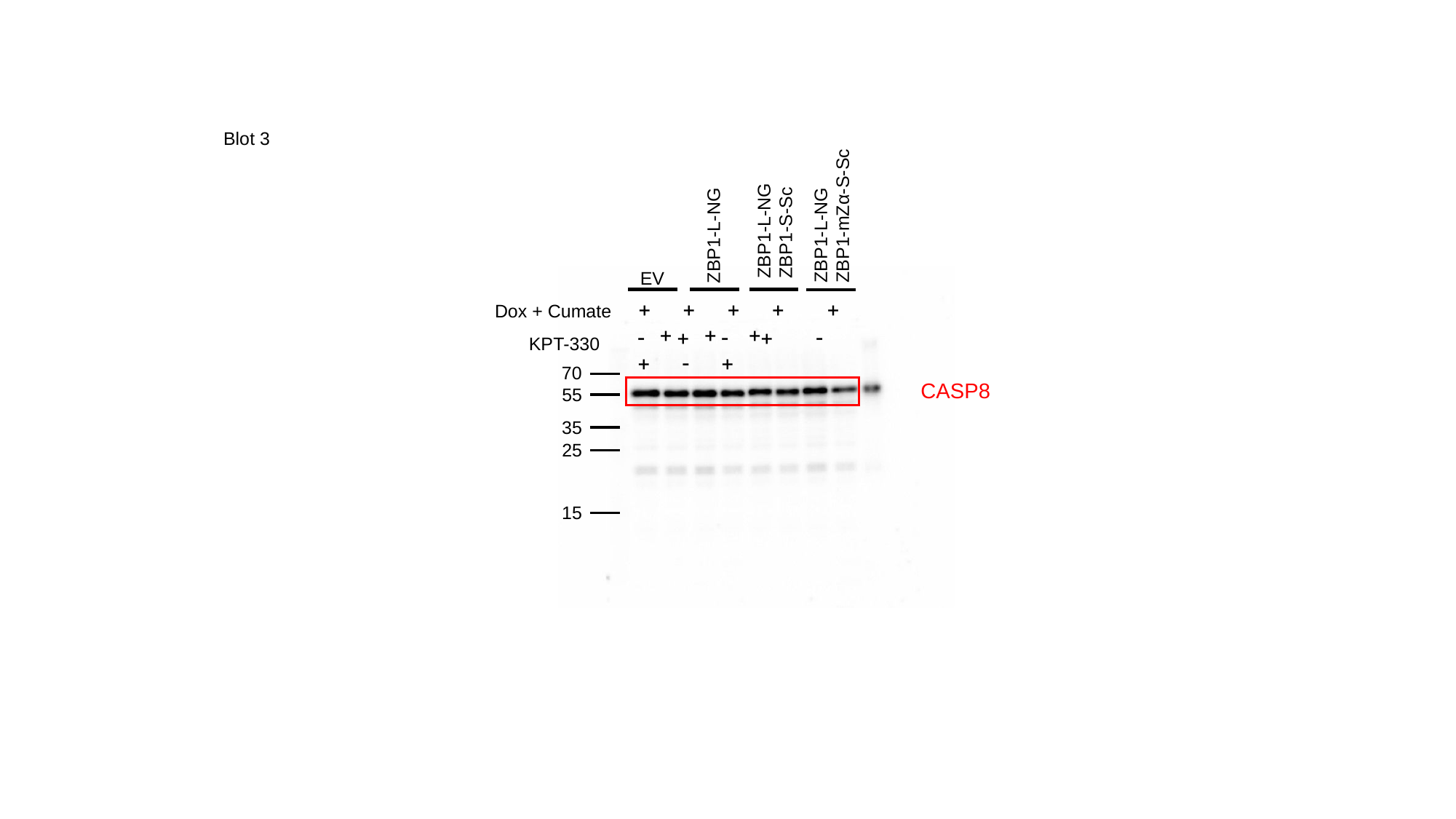

Blot 3
ZBP1-L-NG
ZBP1-mZα-S-Sc
ZBP1-L-NG
ZBP1-S-Sc
ZBP1-L-NG
EV
+ + + + + + + +
Dox + Cumate
- + - + - + - +
KPT-330
70
CASP8
55
35
25
15

Supplement: Supplementary file 7 — Source data Fig. 5 [file 44318_2024_238_MOESM7_ESM.zip › Figure 5/5F/western CASP8.pptx]

## Slide 1
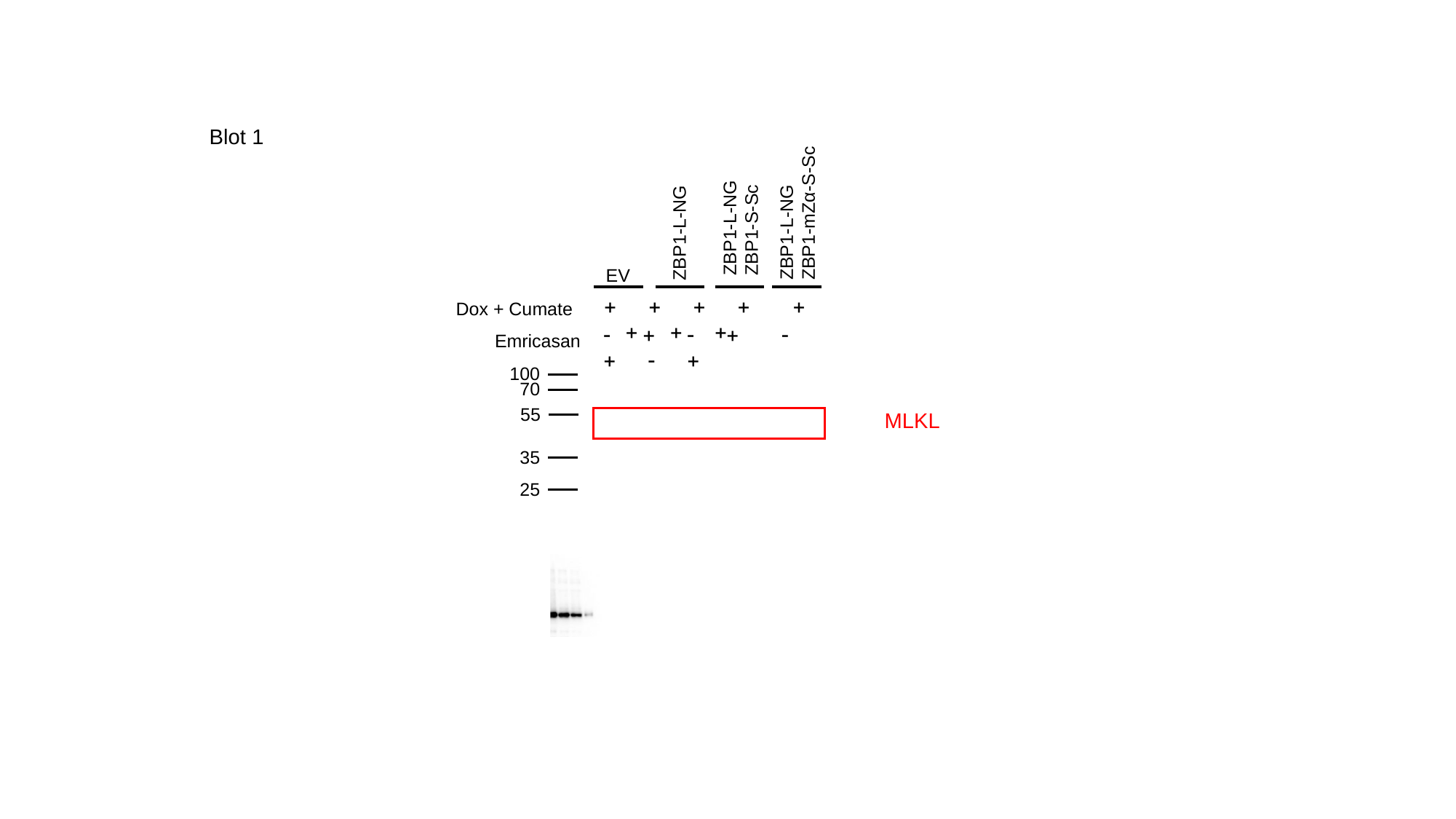

Blot 1
ZBP1-L-NG
ZBP1-mZα-S-Sc
ZBP1-L-NG
ZBP1-S-Sc
ZBP1-L-NG
EV
+ + + + + + + +
Dox + Cumate
- + - + - + - +
Emricasan
100
70
55
MLKL
35
25

Supplement: Supplementary file 7 — Source data Fig. 5 [file 44318_2024_238_MOESM7_ESM.zip › Figure 5/5D/western MLKL.pptx]

## Slide 1
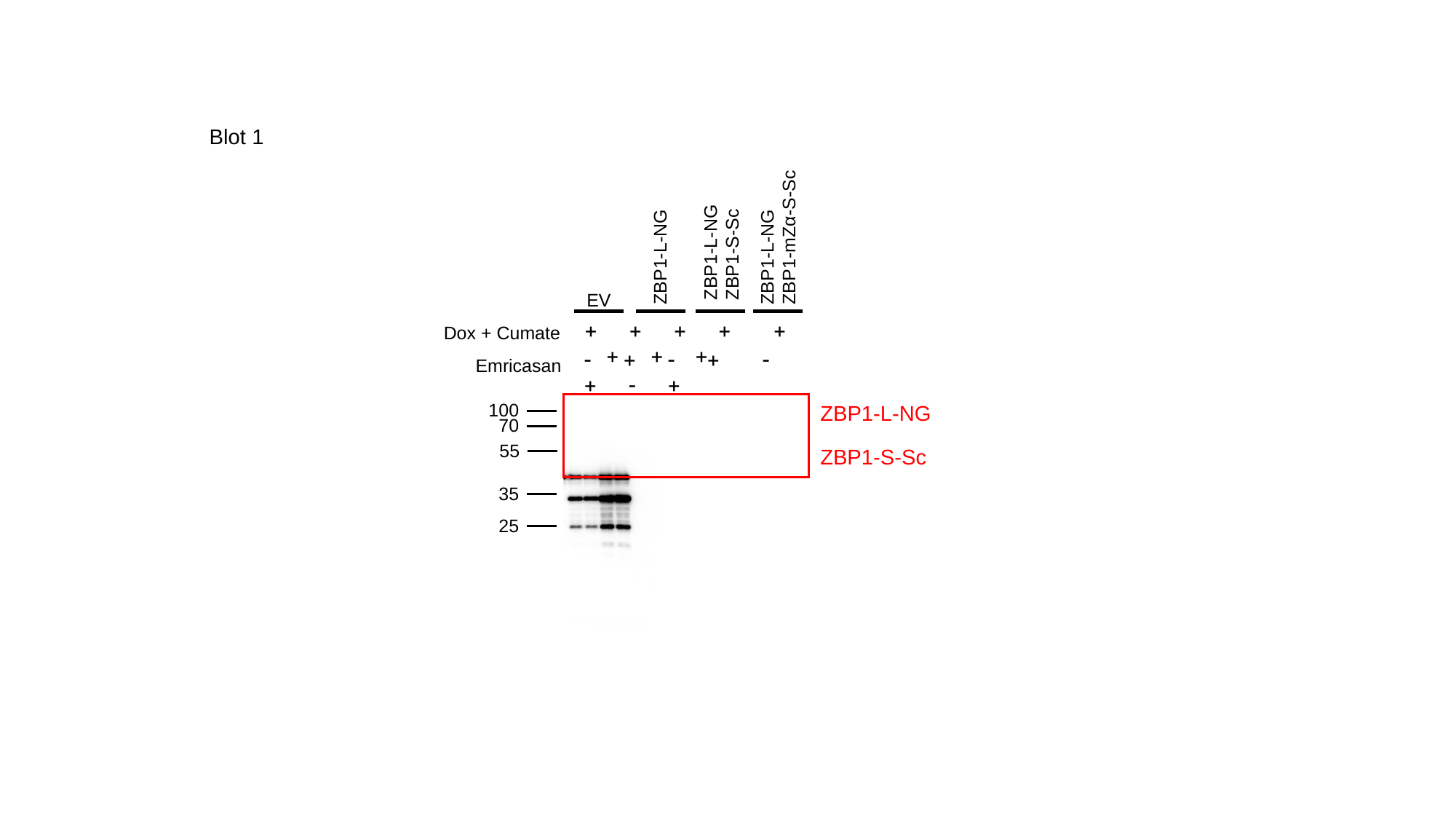

Blot 1
ZBP1-L-NG
ZBP1-mZα-S-Sc
ZBP1-L-NG
ZBP1-S-Sc
ZBP1-L-NG
EV
+ + + + + + + +
Dox + Cumate
- + - + - + - +
Emricasan
100
ZBP1-L-NG
70
55
ZBP1-S-Sc
35
25

Supplement: Supplementary file 7 — Source data Fig. 5 [file 44318_2024_238_MOESM7_ESM.zip › Figure 5/5D/western ZBP1.pptx]

## Slide 1
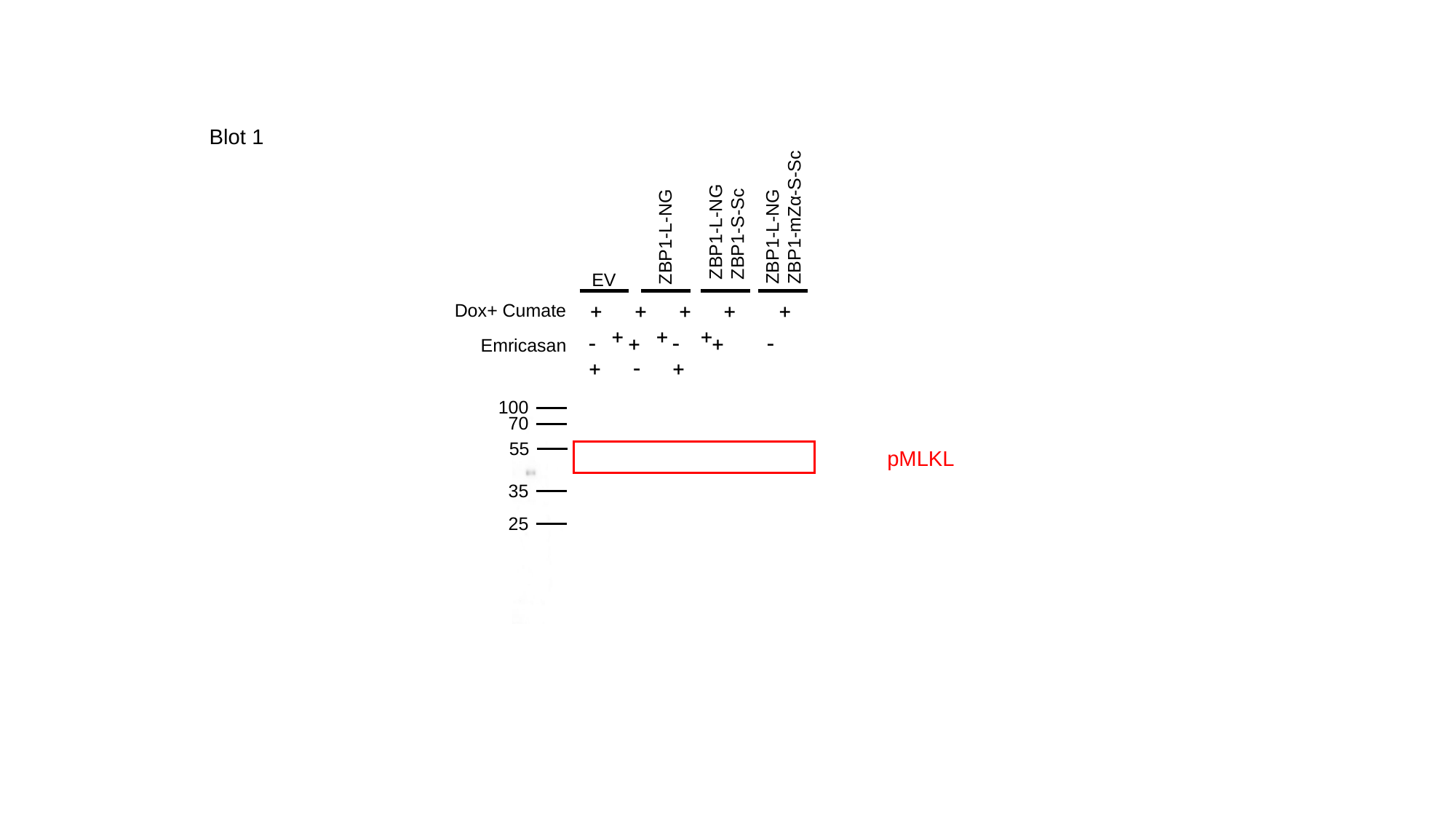

Blot 1
ZBP1-L-NG
ZBP1-mZα-S-Sc
ZBP1-L-NG
ZBP1-S-Sc
ZBP1-L-NG
EV
+ + + + + + + +
Dox+ Cumate
- + - + - + - +
Emricasan
100
70
55
pMLKL
35
25

Supplement: Supplementary file 7 — Source data Fig. 5 [file 44318_2024_238_MOESM7_ESM.zip › Figure 5/5D/western pMLKL.pptx]

## Slide 1
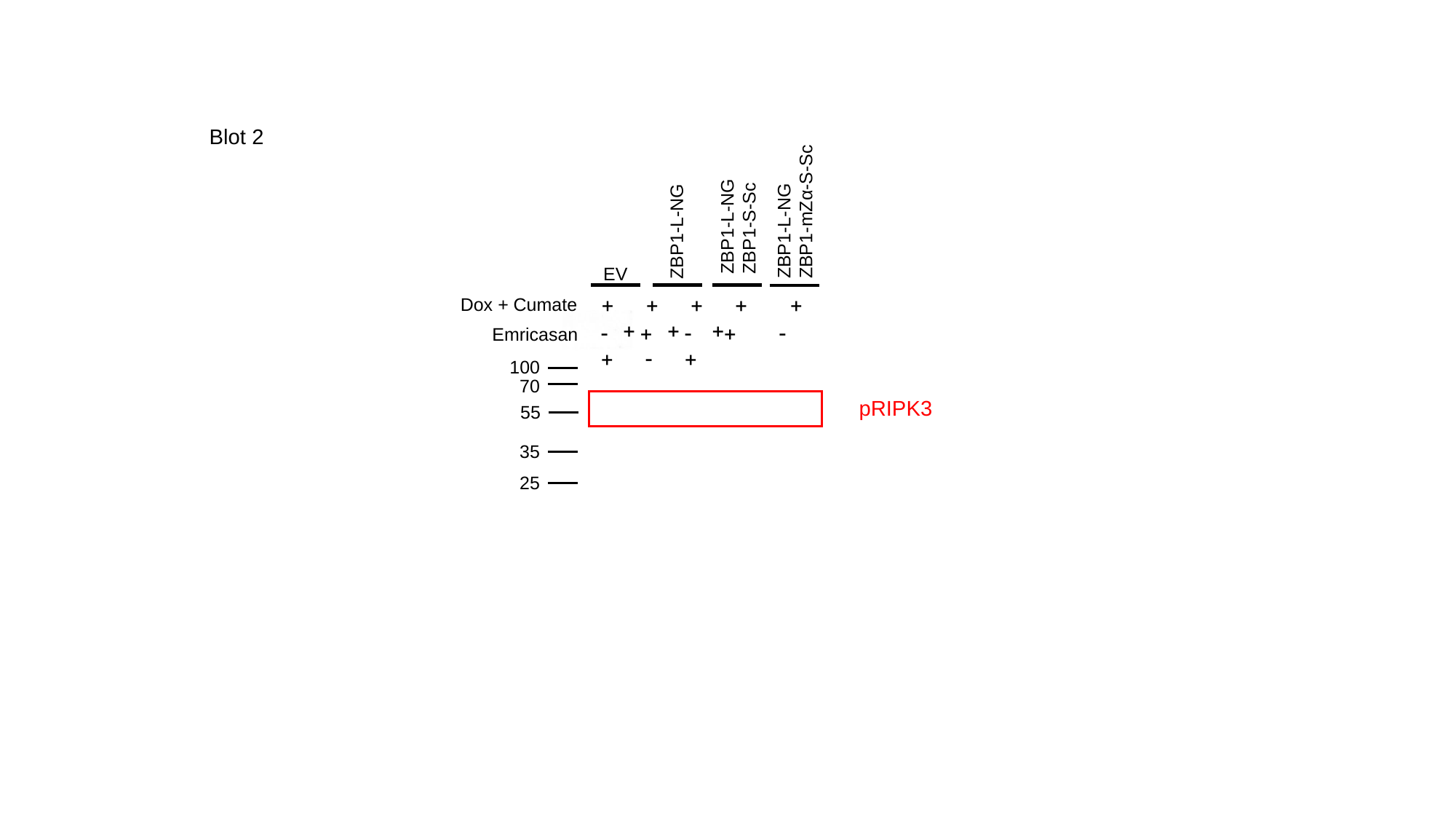

Blot 2
ZBP1-L-NG
ZBP1-mZα-S-Sc
ZBP1-L-NG
ZBP1-S-Sc
ZBP1-L-NG
EV
+ + + + + + + +
Dox + Cumate
- + - + - + - +
Emricasan
100
70
pRIPK3
55
35
25

Supplement: Supplementary file 7 — Source data Fig. 5 [file 44318_2024_238_MOESM7_ESM.zip › Figure 5/5D/western pRIPK3.pptx]

## Slide 1
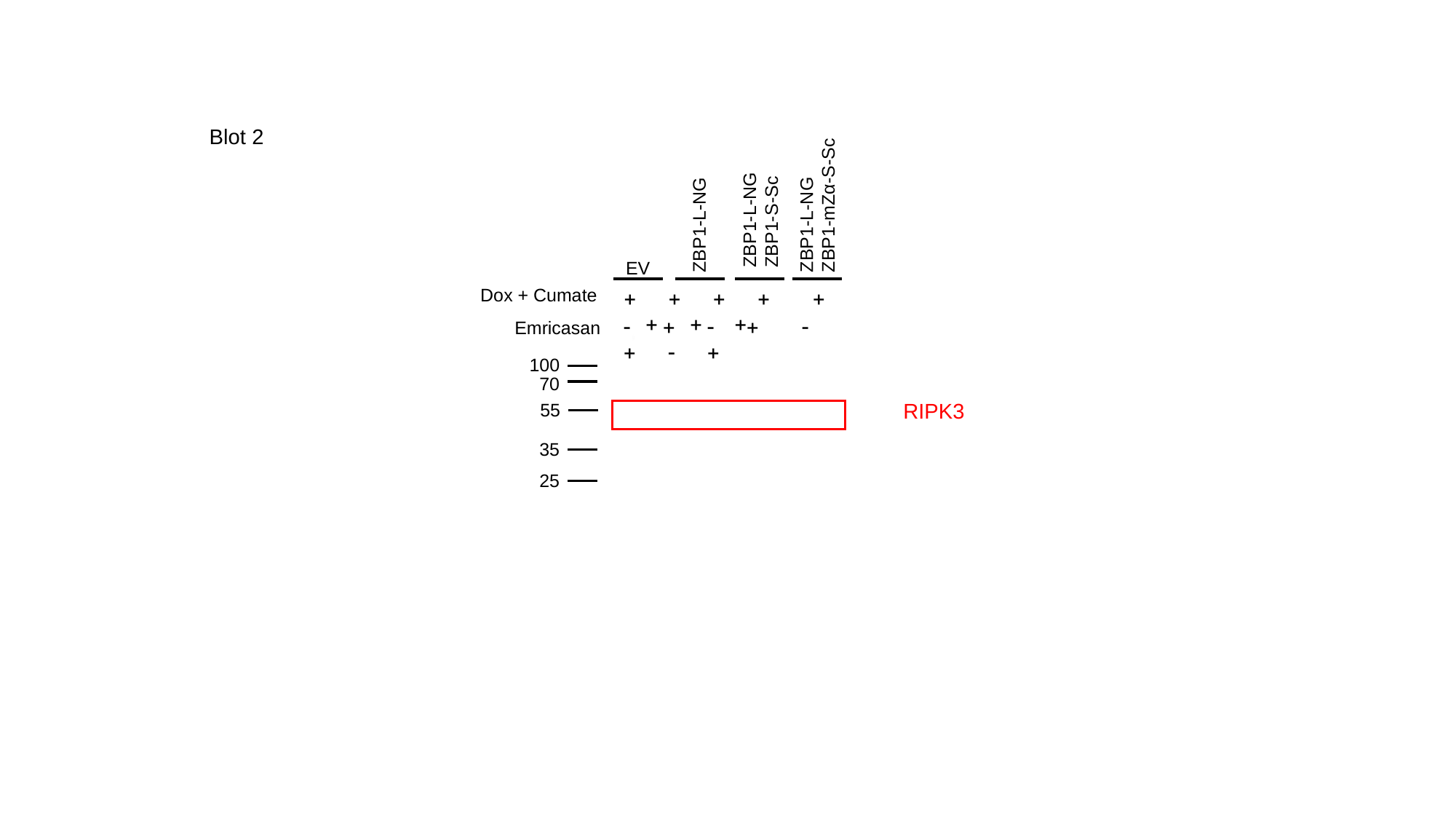

Blot 2
ZBP1-L-NG
ZBP1-mZα-S-Sc
ZBP1-L-NG
ZBP1-S-Sc
ZBP1-L-NG
EV
Dox + Cumate
+ + + + + + + +
- + - + - + - +
Emricasan
100
70
RIPK3
55
35
25

Supplement: Supplementary file 7 — Source data Fig. 5 [file 44318_2024_238_MOESM7_ESM.zip › Figure 5/5D/western RIPK3.pptx]

## Slide 1
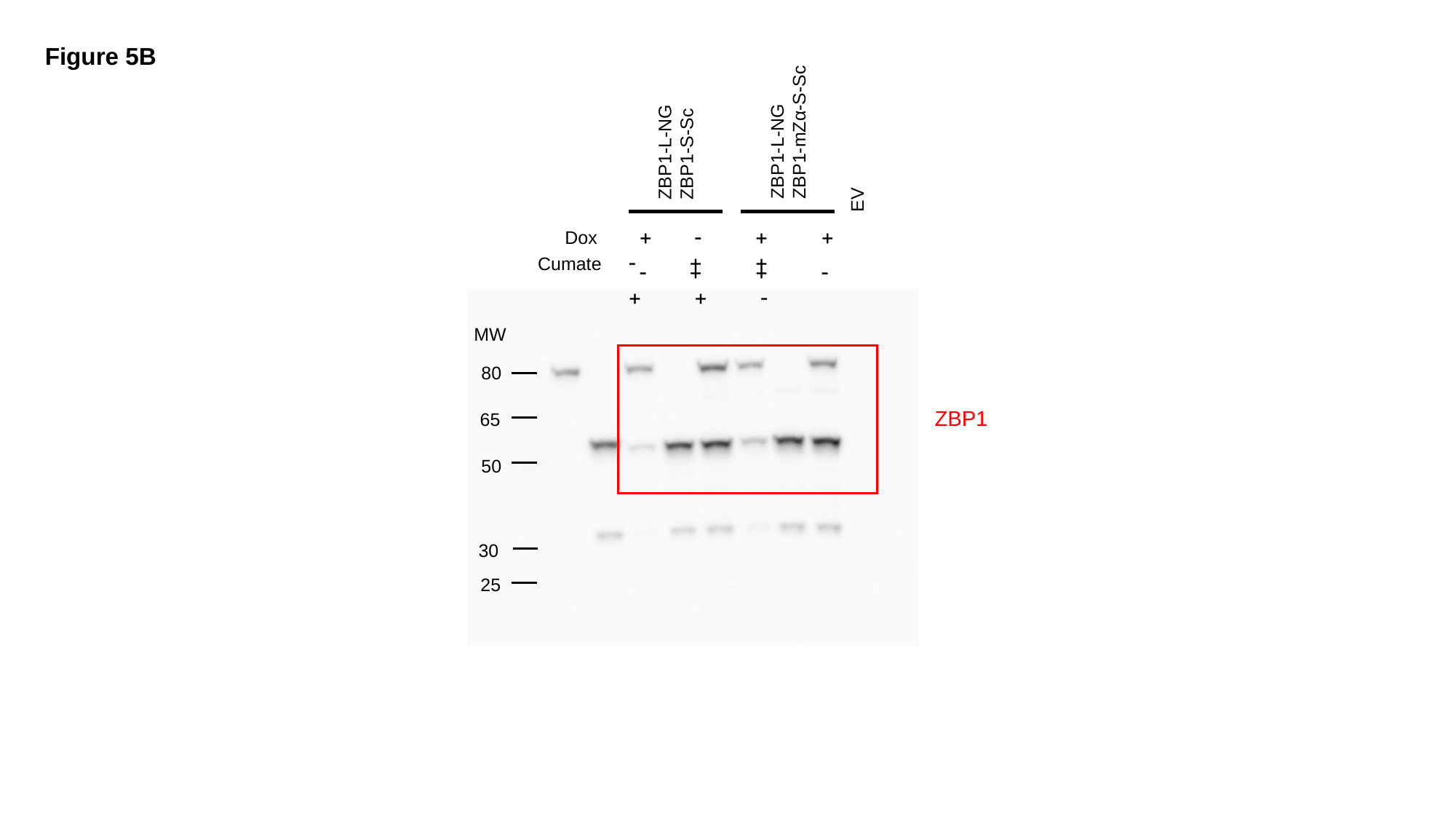

Figure 5B
ZBP1-L-NG
ZBP1-mZα-S-Sc
ZBP1-L-NG
ZBP1-S-Sc
EV
 + - + + - + +
Dox
Cumate
 - + + - + + -
MW
80
ZBP1
65
50
30
25

Supplement: Supplementary file 7 — Source data Fig. 5 [file 44318_2024_238_MOESM7_ESM.zip › Figure 5/5B/western ZBP1.pptx]

## Slide 1
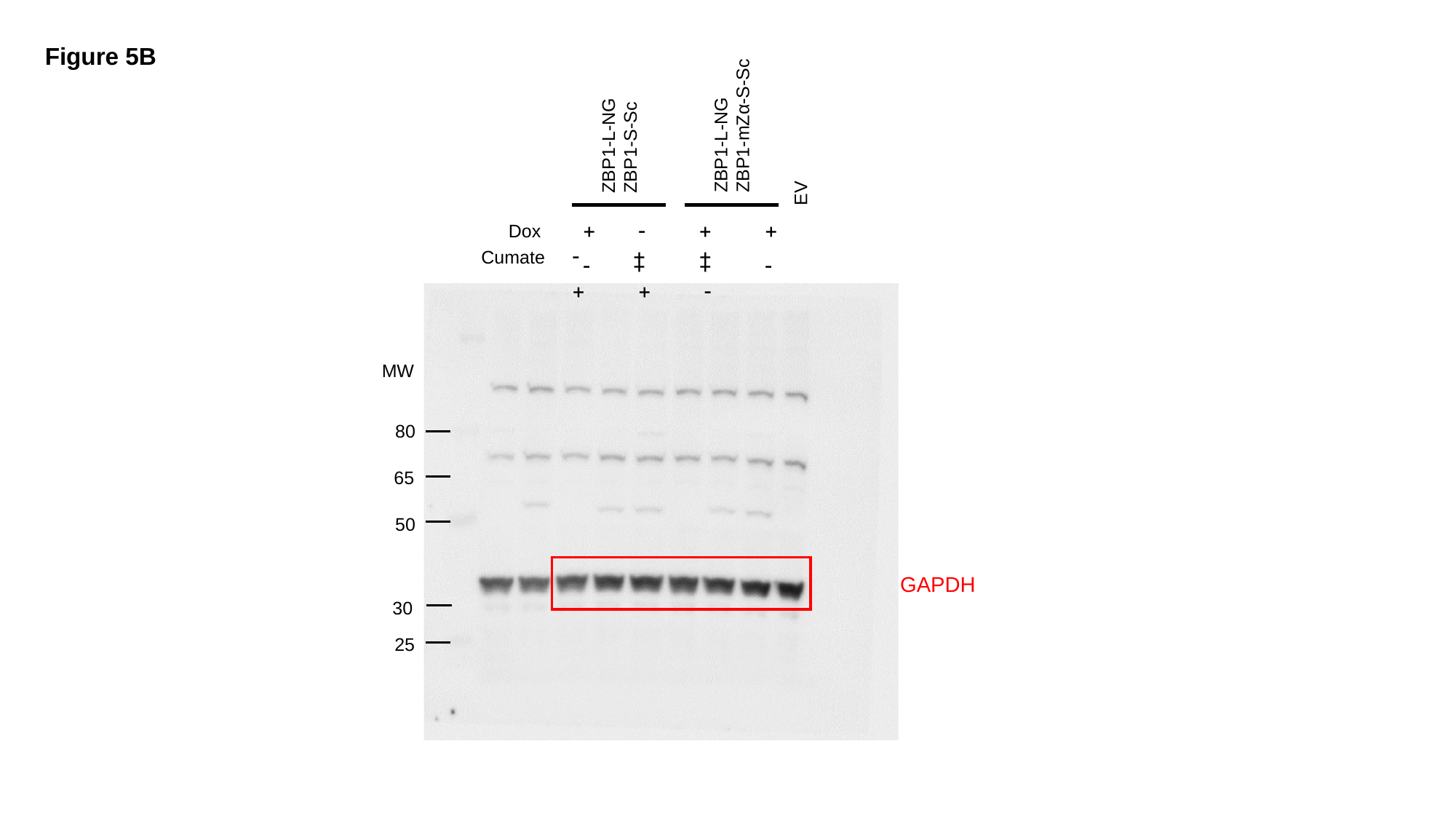

Figure 5B
ZBP1-L-NG
ZBP1-mZα-S-Sc
ZBP1-L-NG
ZBP1-S-Sc
EV
 + - + + - + +
Dox
Cumate
 - + + - + + -
MW
80
65
50
GAPDH
30
25

Supplement: Supplementary file 7 — Source data Fig. 5 [file 44318_2024_238_MOESM7_ESM.zip › Figure 5/5B/western GAPDH.pptx]

## Slide 1
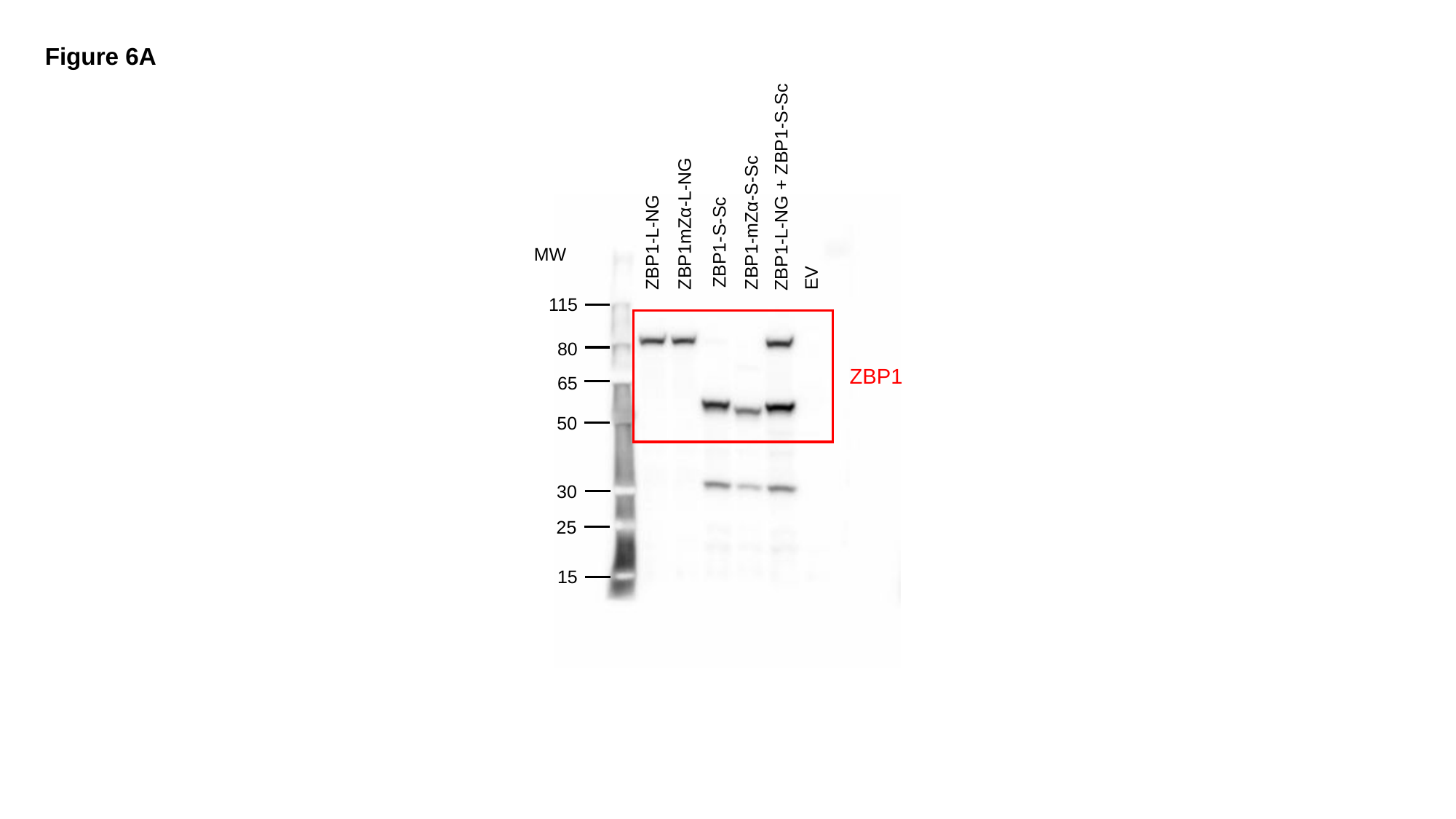

Figure 6A
ZBP1-L-NG + ZBP1-S-Sc
ZBP1-mZα-S-Sc
ZBP1mZα-L-NG
ZBP1-S-Sc
ZBP1-L-NG
MW
EV
115
80
ZBP1
65
50
30
25
15

Supplement: Supplementary file 8 — Source data Fig. 6 [file 44318_2024_238_MOESM8_ESM.zip › Figure 6/6A/ZBP1.pptx]

## Slide 1
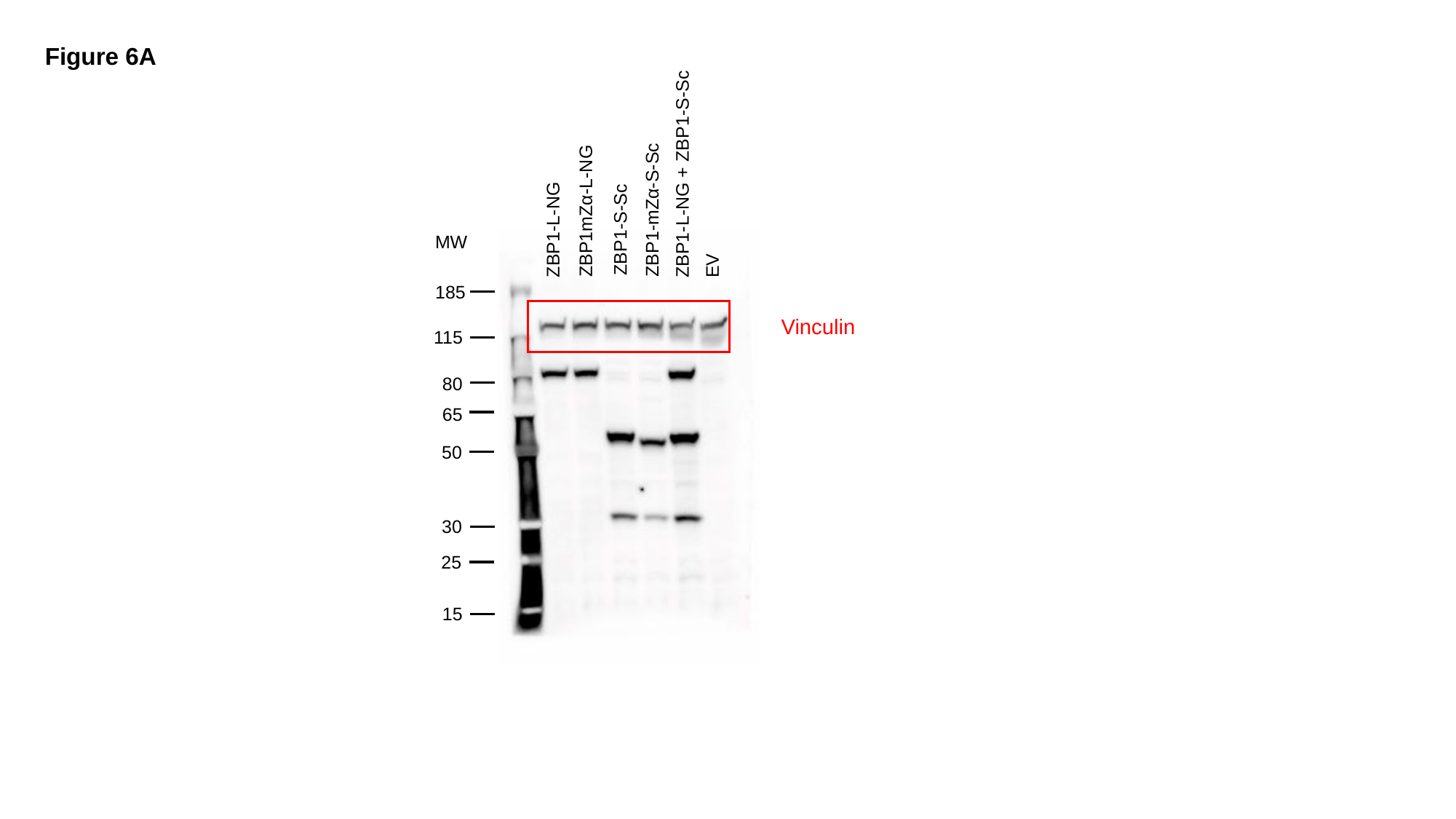

Figure 6A
ZBP1-L-NG + ZBP1-S-Sc
ZBP1-mZα-S-Sc
ZBP1mZα-L-NG
ZBP1-S-Sc
ZBP1-L-NG
MW
EV
185
Vinculin
115
80
65
50
30
25
15

Supplement: Supplementary file 8 — Source data Fig. 6 [file 44318_2024_238_MOESM8_ESM.zip › Figure 6/6A/Vinculin.pptx]

## Slide 1
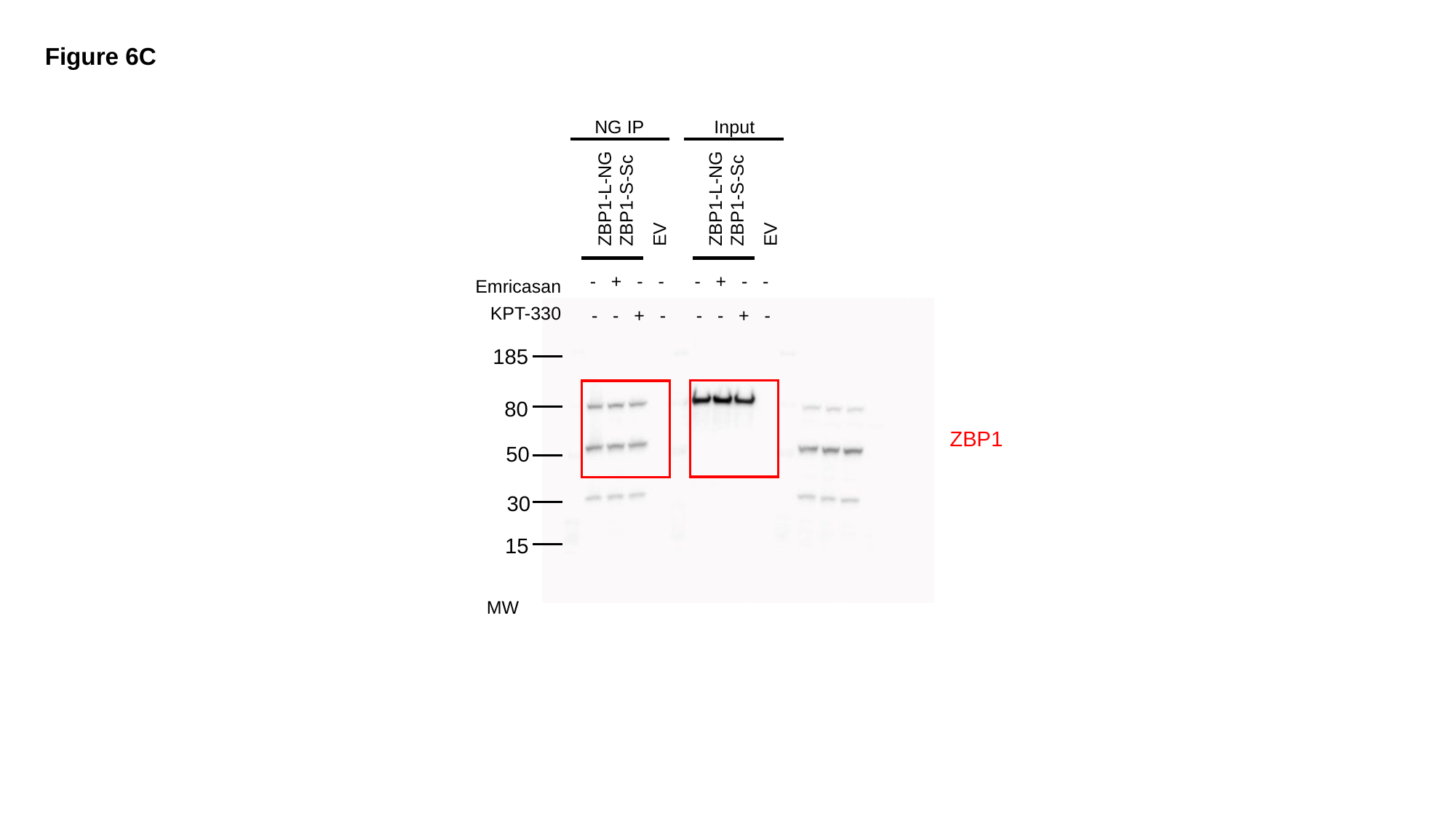

Figure 6C
NG IP
Input
ZBP1-L-NG
ZBP1-S-Sc
ZBP1-L-NG
ZBP1-S-Sc
EV
EV
- + - - - + - -
Emricasan
KPT-330
- - + - - - + -
185
80
ZBP1
50
30
15
MW

Supplement: Supplementary file 8 — Source data Fig. 6 [file 44318_2024_238_MOESM8_ESM.zip › Figure 6/6C/Neon Green IP/western ZBP1.pptx]

## Slide 1
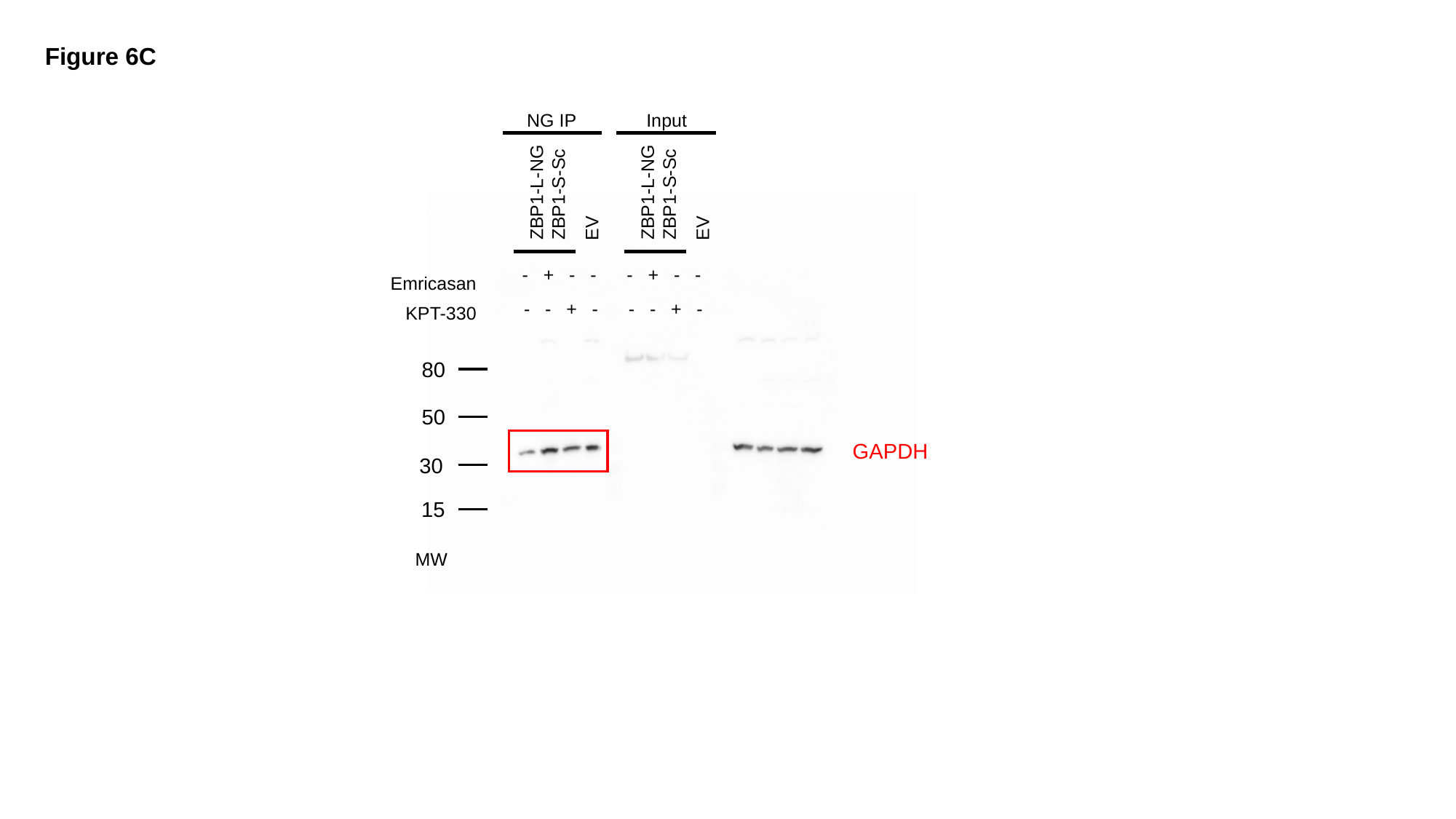

Figure 6C
NG IP
Input
ZBP1-L-NG
ZBP1-S-Sc
ZBP1-L-NG
ZBP1-S-Sc
EV
EV
- + - - - + - -
Emricasan
- - + - - - + -
KPT-330
80
50
GAPDH
30
15
MW

Supplement: Supplementary file 8 — Source data Fig. 6 [file 44318_2024_238_MOESM8_ESM.zip › Figure 6/6C/Neon Green IP/western GAPDH.pptx]

## Slide 1
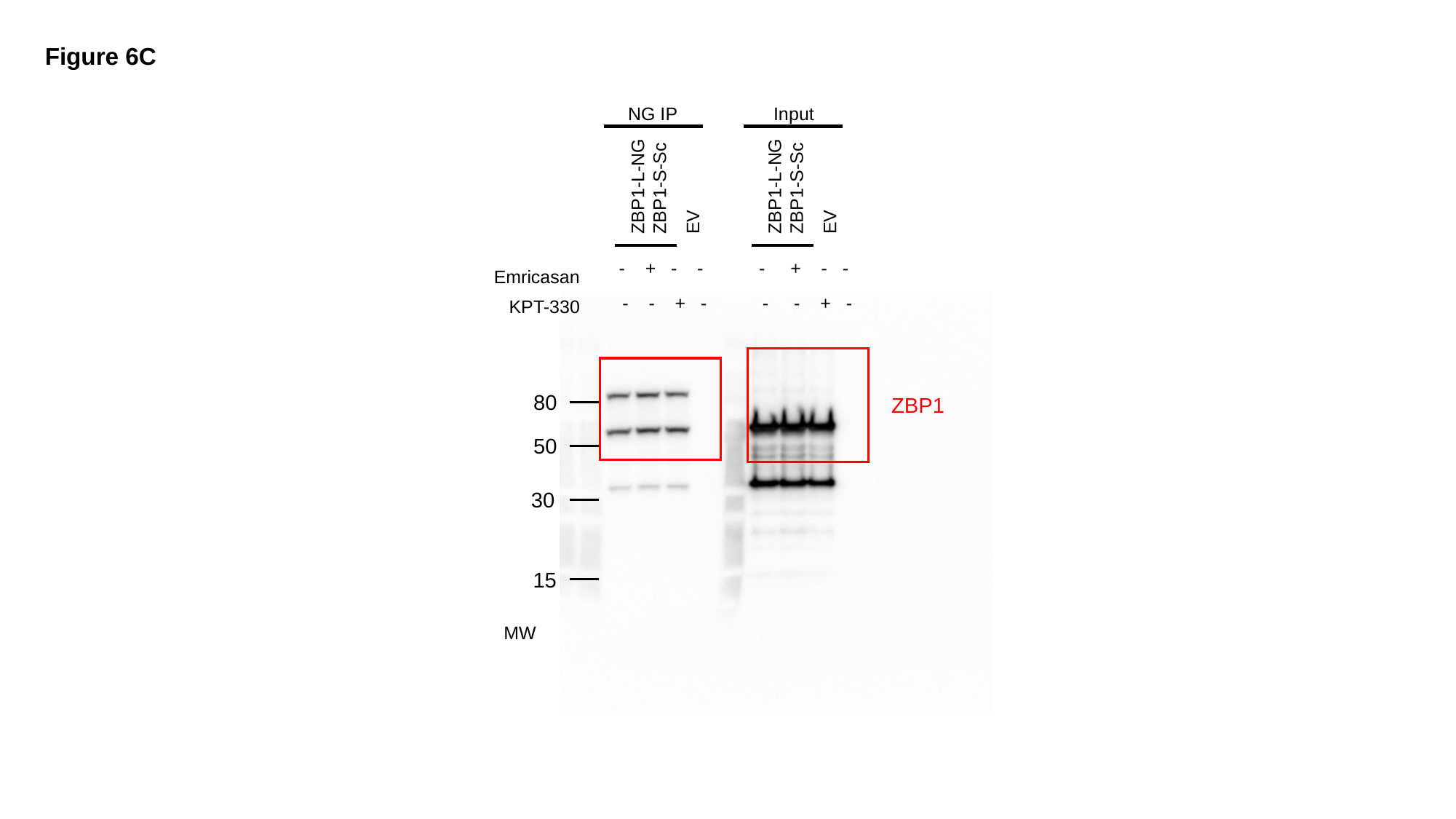

Figure 6C
NG IP
Input
ZBP1-L-NG
ZBP1-S-Sc
ZBP1-L-NG
ZBP1-S-Sc
EV
EV
- + - - - + - -
Emricasan
- - + - - - + -
KPT-330
80
ZBP1
50
30
15
MW

Supplement: Supplementary file 8 — Source data Fig. 6 [file 44318_2024_238_MOESM8_ESM.zip › Figure 6/6C/Scarlet IP/western ZBP1.pptx]

## Slide 1
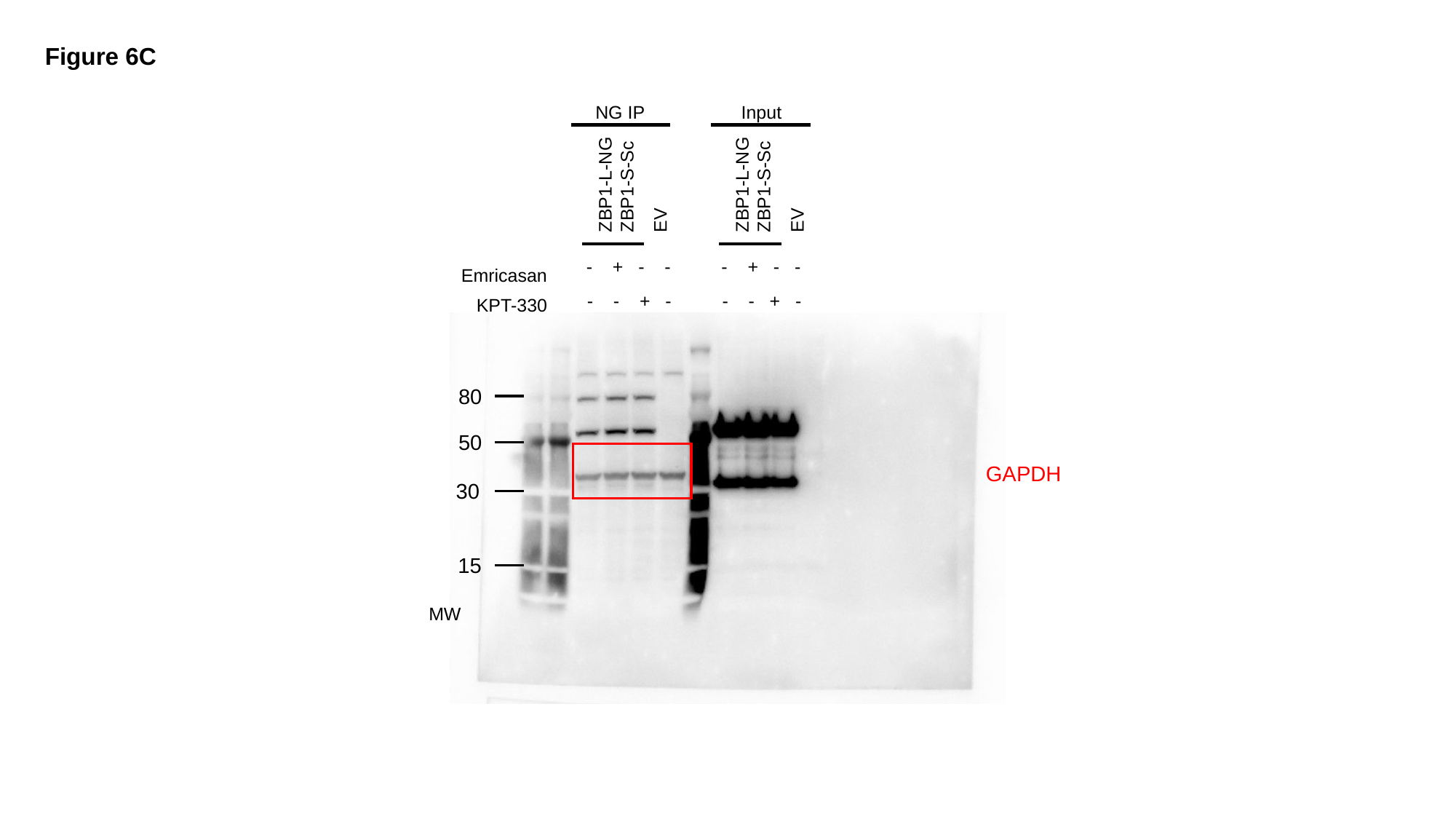

Figure 6C
NG IP
Input
ZBP1-L-NG
ZBP1-S-Sc
ZBP1-L-NG
ZBP1-S-Sc
EV
EV
- + - - - + - -
Emricasan
- - + - - - + -
KPT-330
80
50
GAPDH
30
15
MW

Supplement: Supplementary file 8 — Source data Fig. 6 [file 44318_2024_238_MOESM8_ESM.zip › Figure 6/6C/Scarlet IP/western GAPDH.pptx]

## Slide 1
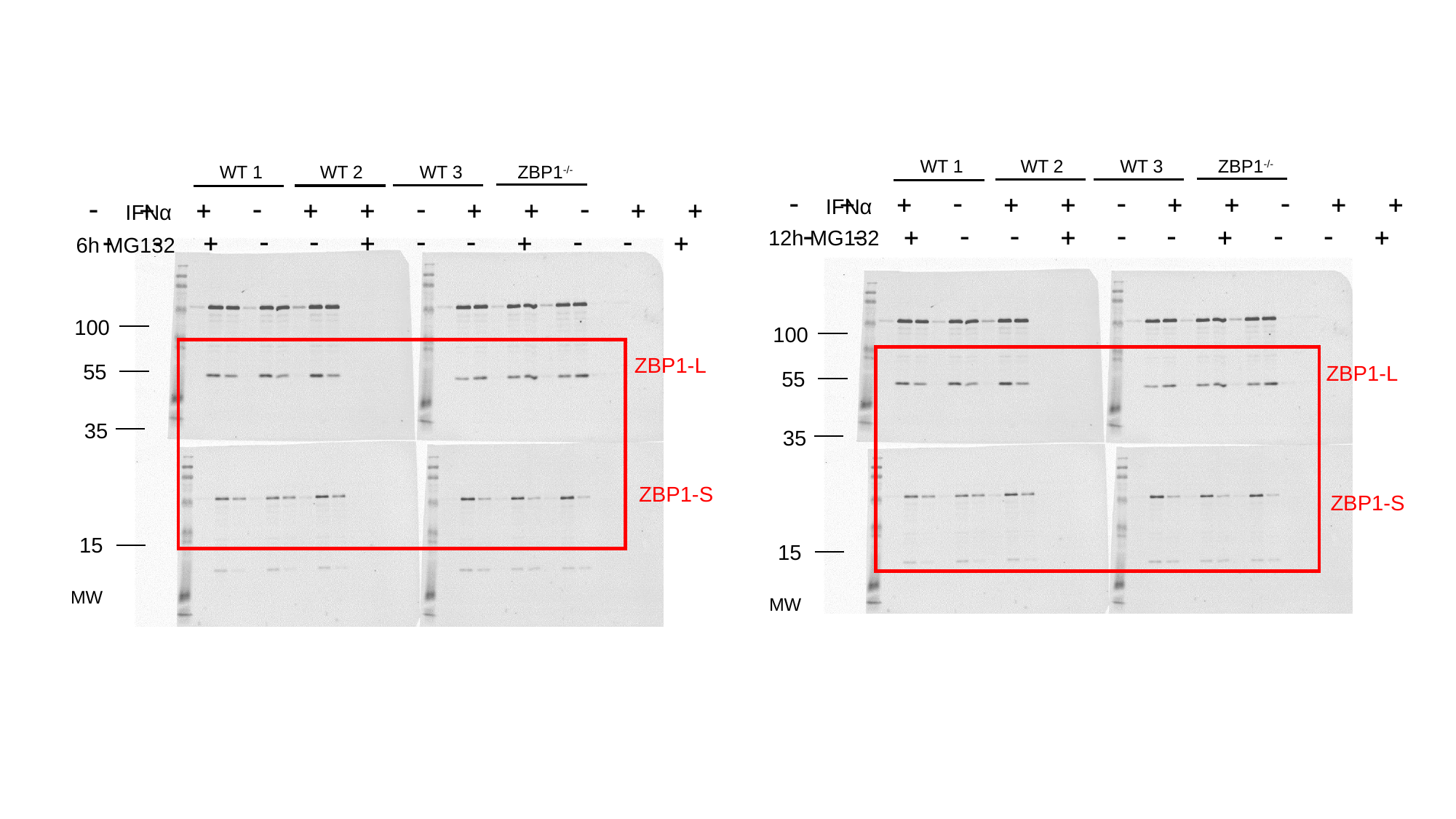

WT 1
WT 2
WT 3
ZBP1-/-
WT 1
WT 2
WT 3
ZBP1-/-
- + + - + + - + + - + +
- + + - + + - + + - + +
IFNα
IFNα
- - + - - + - - + - - +
12h MG132
- - + - - + - - + - - +
6h MG132
100
100
ZBP1-L
55
ZBP1-L
55
35
35
ZBP1-S
ZBP1-S
15
15
MW
MW

Supplement: Supplementary file 9 — EV Figure Source Data [file 44318_2024_238_MOESM9_ESM.zip › Figure EV1/1D/western ZBP1.pptx]

## Slide 1
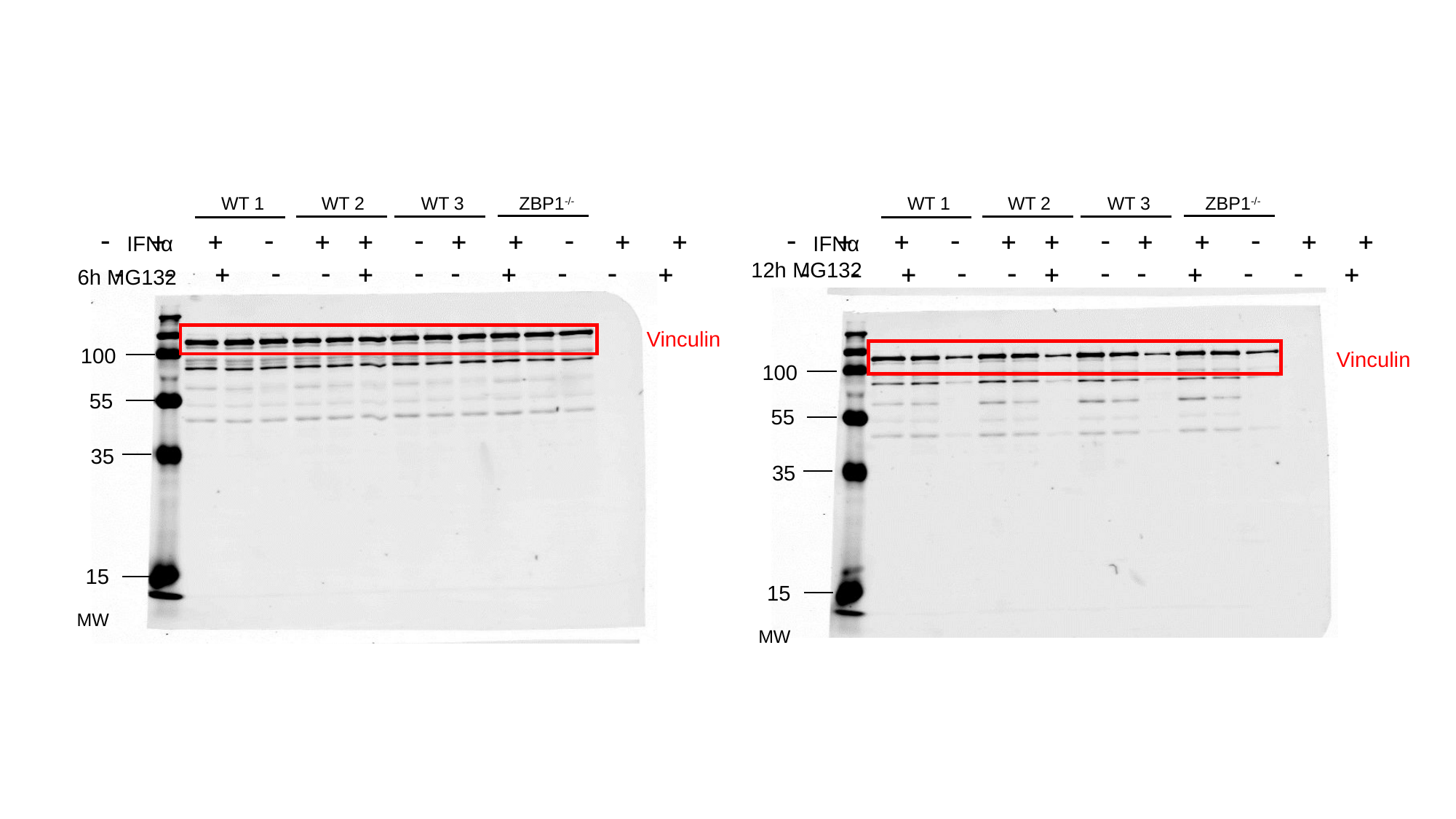

WT 1
WT 1
WT 2
WT 2
WT 3
WT 3
ZBP1-/-
ZBP1-/-
- + + - + + - + + - + +
- + + - + + - + + - + +
IFNα
IFNα
- - + - - + - - + - - +
- - + - - + - - + - - +
12h MG132
6h MG132
Vinculin
100
Vinculin
100
55
55
35
35
15
15
MW
MW

Supplement: Supplementary file 9 — EV Figure Source Data [file 44318_2024_238_MOESM9_ESM.zip › Figure EV1/1D/western VINCULIN.pptx]

## Slide 1
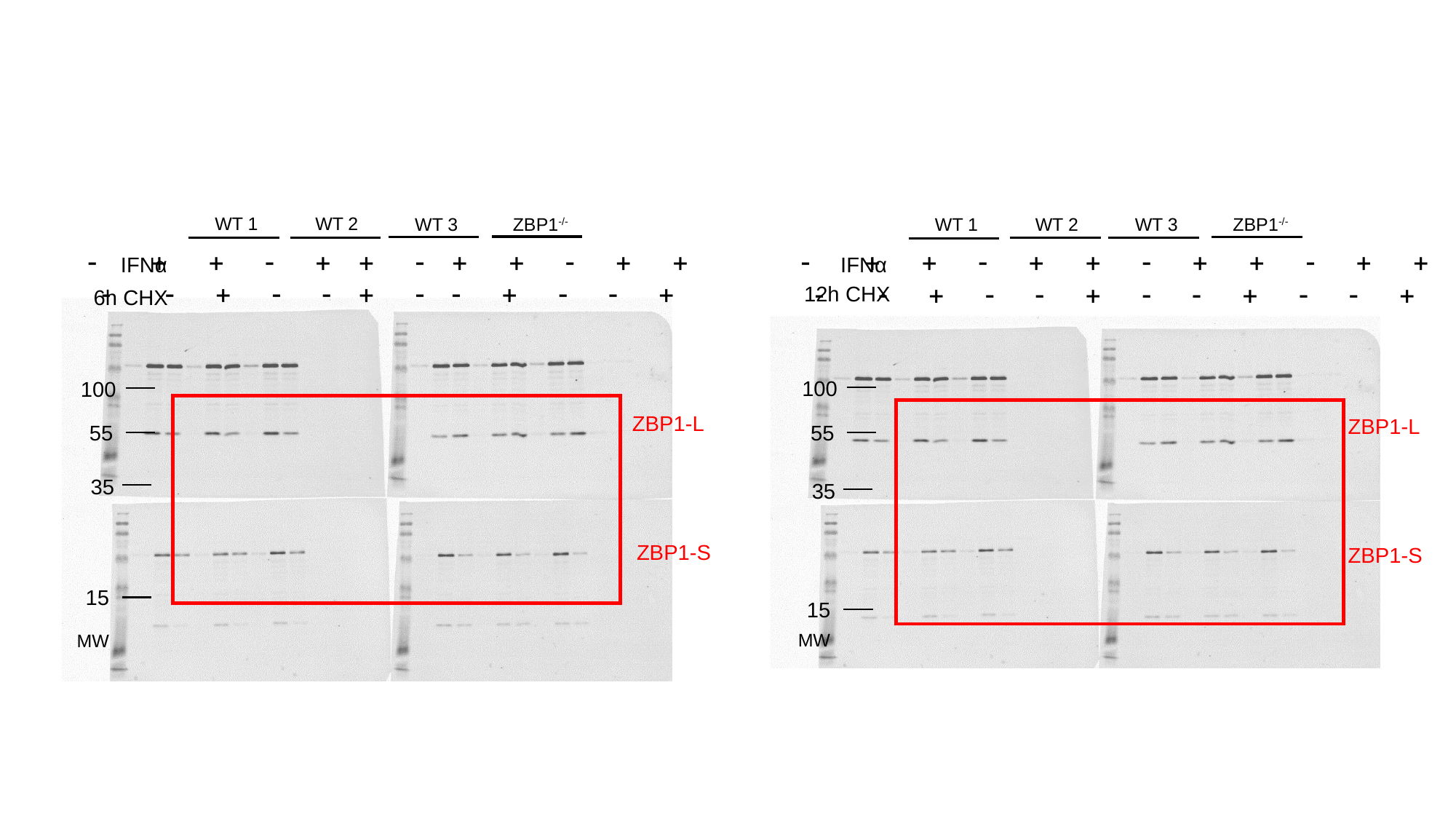

WT 1
WT 2
WT 3
ZBP1-/-
WT 1
WT 2
WT 3
ZBP1-/-
- + + - + + - + + - + +
- + + - + + - + + - + +
IFNα
IFNα
- - + - - + - - + - - +
- - + - - + - - + - - +
12h CHX
6h CHX
100
100
ZBP1-L
ZBP1-L
55
55
35
35
ZBP1-S
ZBP1-S
15
15
MW
MW

Supplement: Supplementary file 9 — EV Figure Source Data [file 44318_2024_238_MOESM9_ESM.zip › Figure EV1/1C/western ZBP1.pptx]

## Slide 1
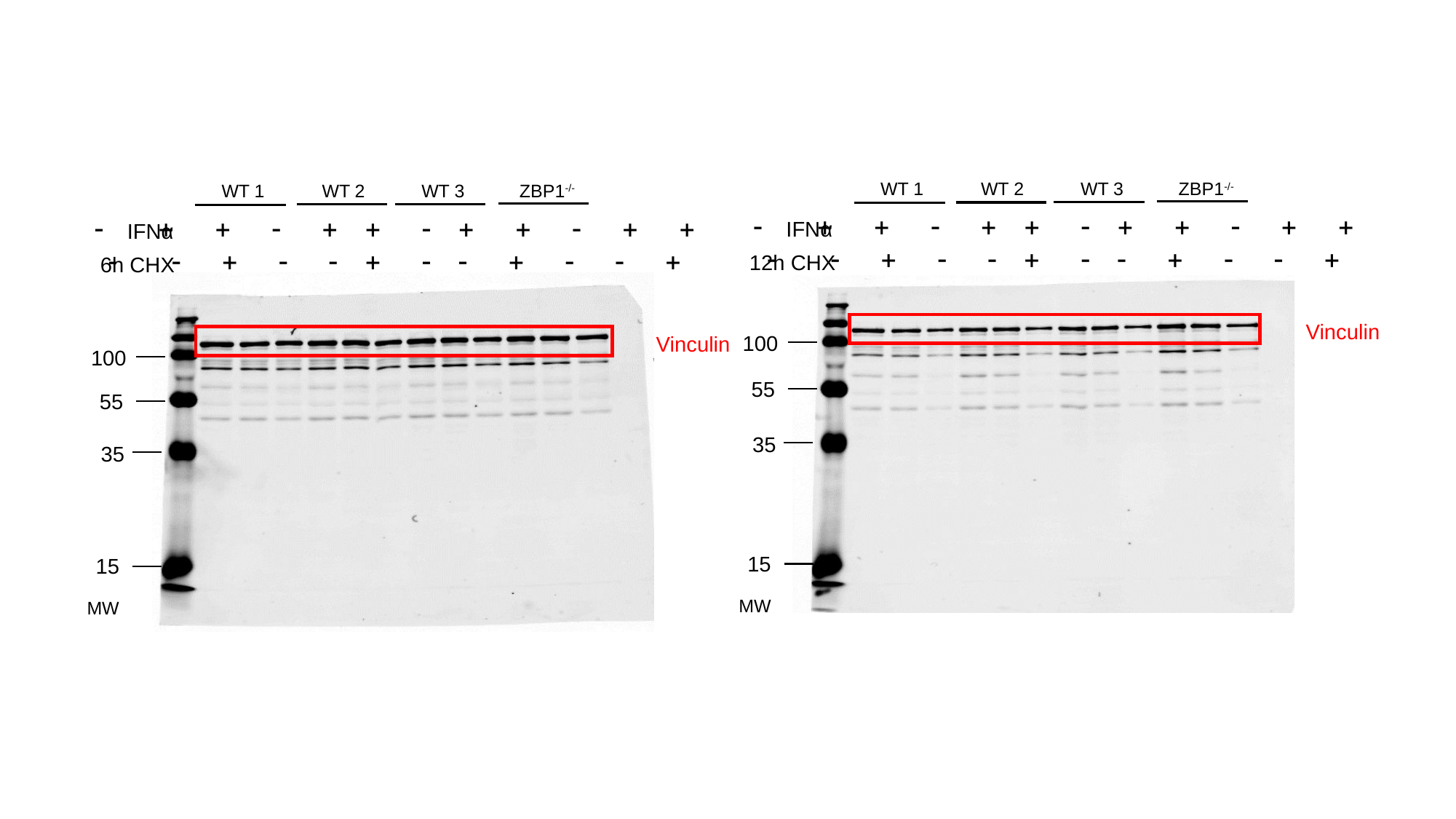

WT 1
WT 2
WT 3
ZBP1-/-
WT 1
WT 2
WT 3
ZBP1-/-
- + + - + + - + + - + +
- + + - + + - + + - + +
IFNα
IFNα
- - + - - + - - + - - +
- - + - - + - - + - - +
12h CHX
6h CHX
Vinculin
100
Vinculin
100
55
55
35
35
15
15
MW
MW

Supplement: Supplementary file 9 — EV Figure Source Data [file 44318_2024_238_MOESM9_ESM.zip › Figure EV1/1C/western VINCULIN.pptx]

## Slide 1
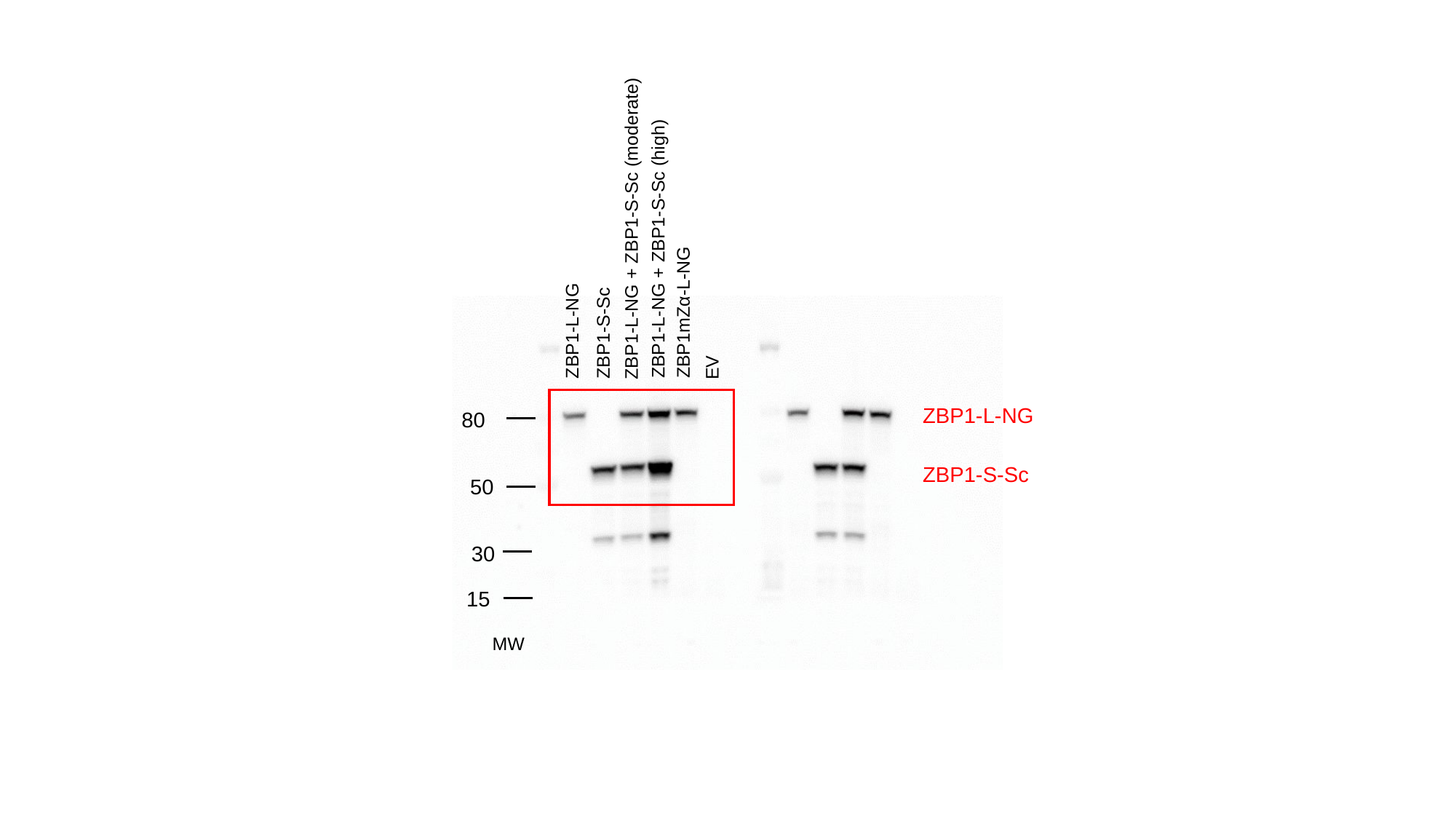

ZBP1-L-NG + ZBP1-S-Sc (high)
ZBP1-L-NG + ZBP1-S-Sc (moderate)
ZBP1mZα-L-NG
ZBP1-L-NG
ZBP1-S-Sc
EV
ZBP1-L-NG
80
ZBP1-S-Sc
50
30
15
MW

Supplement: Supplementary file 9 — EV Figure Source Data [file 44318_2024_238_MOESM9_ESM.zip › Figure EV3/EV3B/western ZBP1.pptx]

## Slide 1
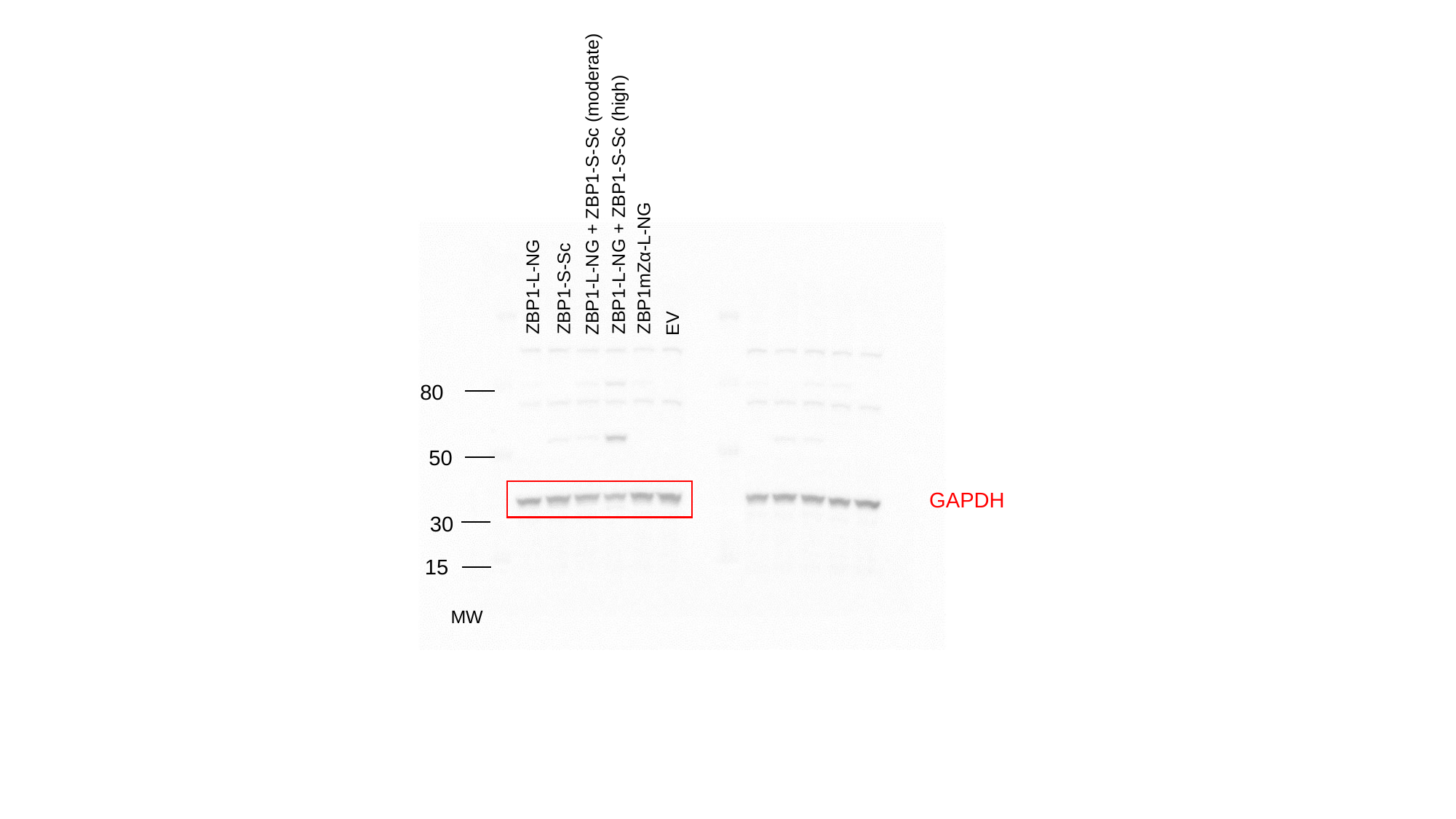

ZBP1-L-NG + ZBP1-S-Sc (high)
ZBP1-L-NG + ZBP1-S-Sc (moderate)
ZBP1mZα-L-NG
ZBP1-L-NG
ZBP1-S-Sc
EV
80
50
GAPDH
30
15
MW

Supplement: Supplementary file 9 — EV Figure Source Data [file 44318_2024_238_MOESM9_ESM.zip › Figure EV3/EV3B/western GAPDH.pptx]
