# Supplementary material for: A shorter splicing isoform antagonizes ZBP1 to modulate cell death and inflammatory responses
Source: EMBO J. 2024 Sep 19;43(21):12. doi: 10.1038/s44318-024-00238-7 (PMC11535224; doi:10.1038/s44318-024-00238-7)
Supplement: Supplementary file 5 — Source data Fig. 3 [file 44318_2024_238_MOESM5_ESM.zip › Figure 3/3A/Mice pictures.pptx]

## Slide 1
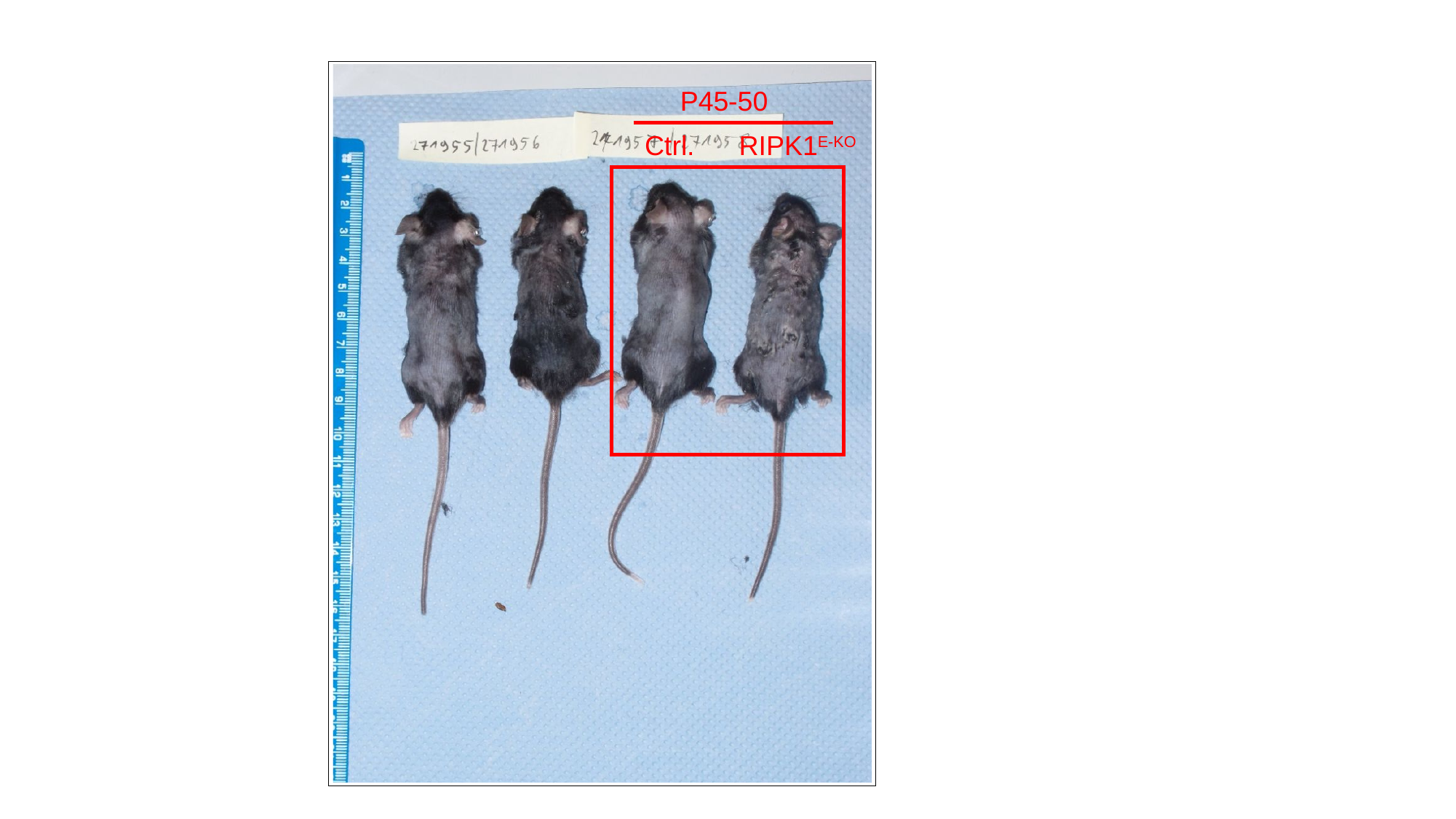

P45-50
RIPK1E-KO
Ctrl.

## Slide 2
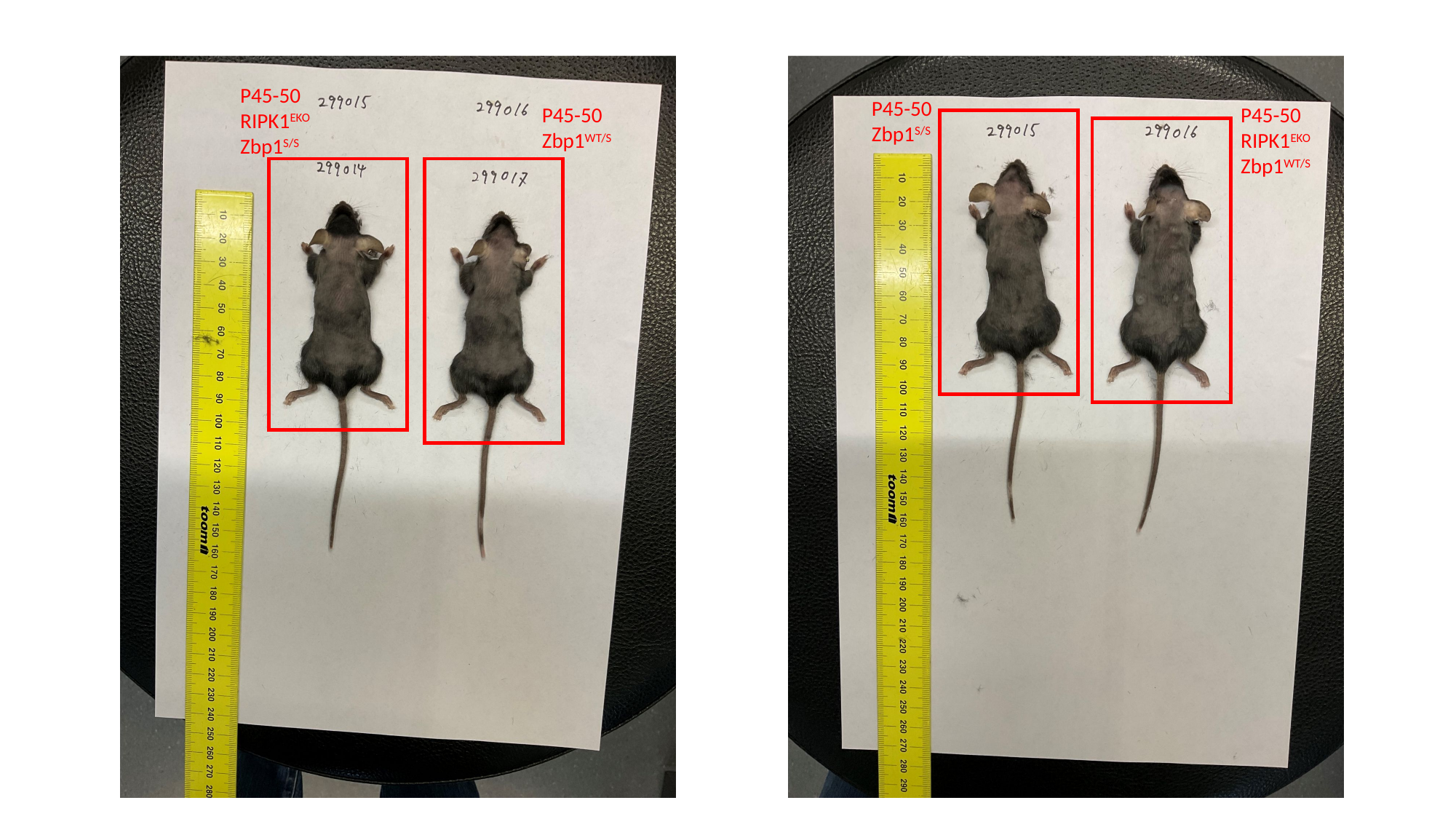

P45-50
RIPK1EKO
Zbp1S/S
P45-50
Zbp1S/S
P45-50
Zbp1WT/S
P45-50
RIPK1EKO
Zbp1WT/S

## Slide 3
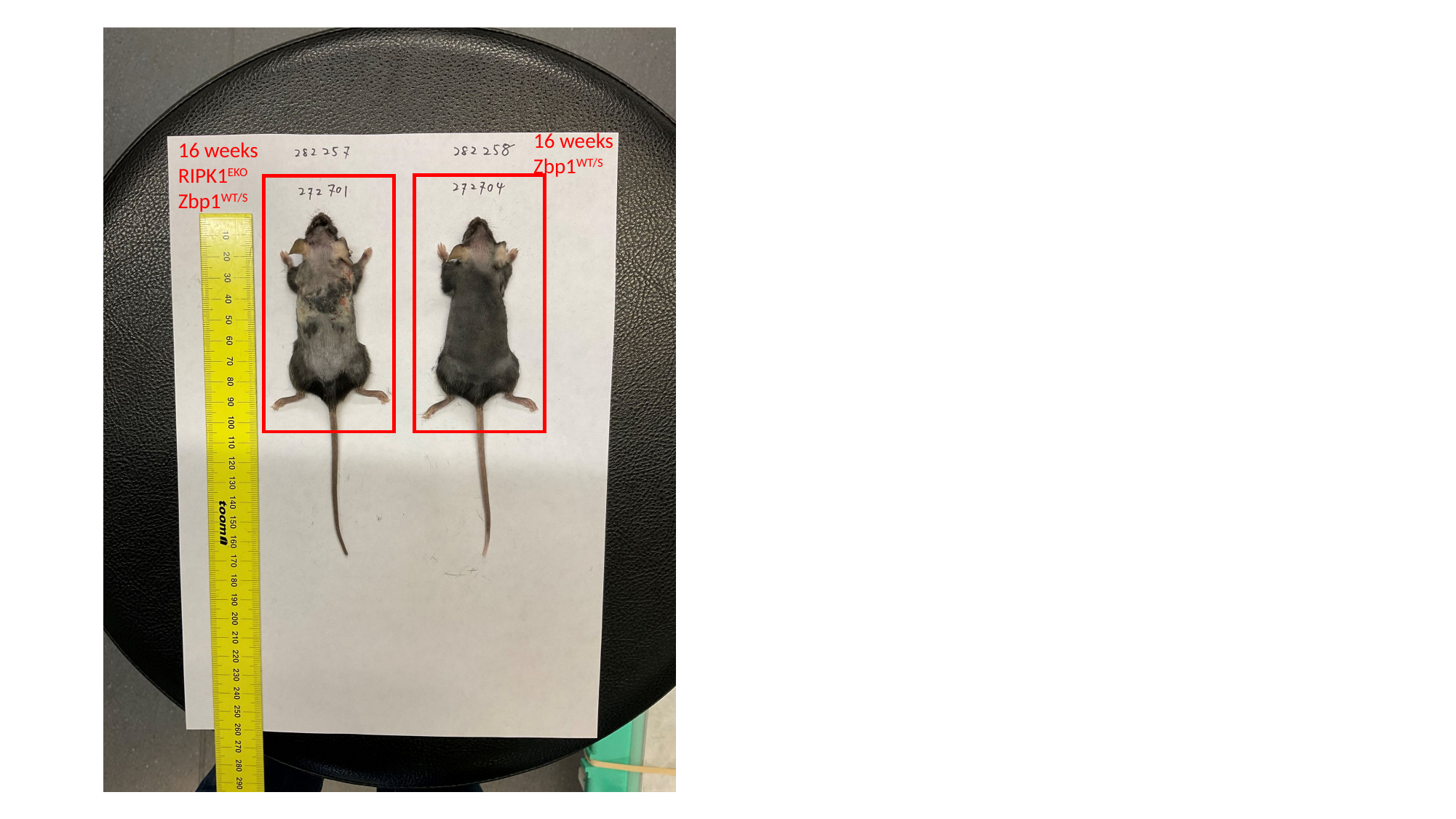

16 weeks
Zbp1WT/S
16 weeks
RIPK1EKO
Zbp1WT/S
