# Supplementary material for: A shorter splicing isoform antagonizes ZBP1 to modulate cell death and inflammatory responses
Source: EMBO J. 2024 Sep 19;43(21):12. doi: 10.1038/s44318-024-00238-7 (PMC11535224; doi:10.1038/s44318-024-00238-7)
Supplement: Supplementary file 6 — Source data Fig. 4 [file 44318_2024_238_MOESM6_ESM.zip › Figure 4/4A/Mice pictures.pptx]

## Slide 1
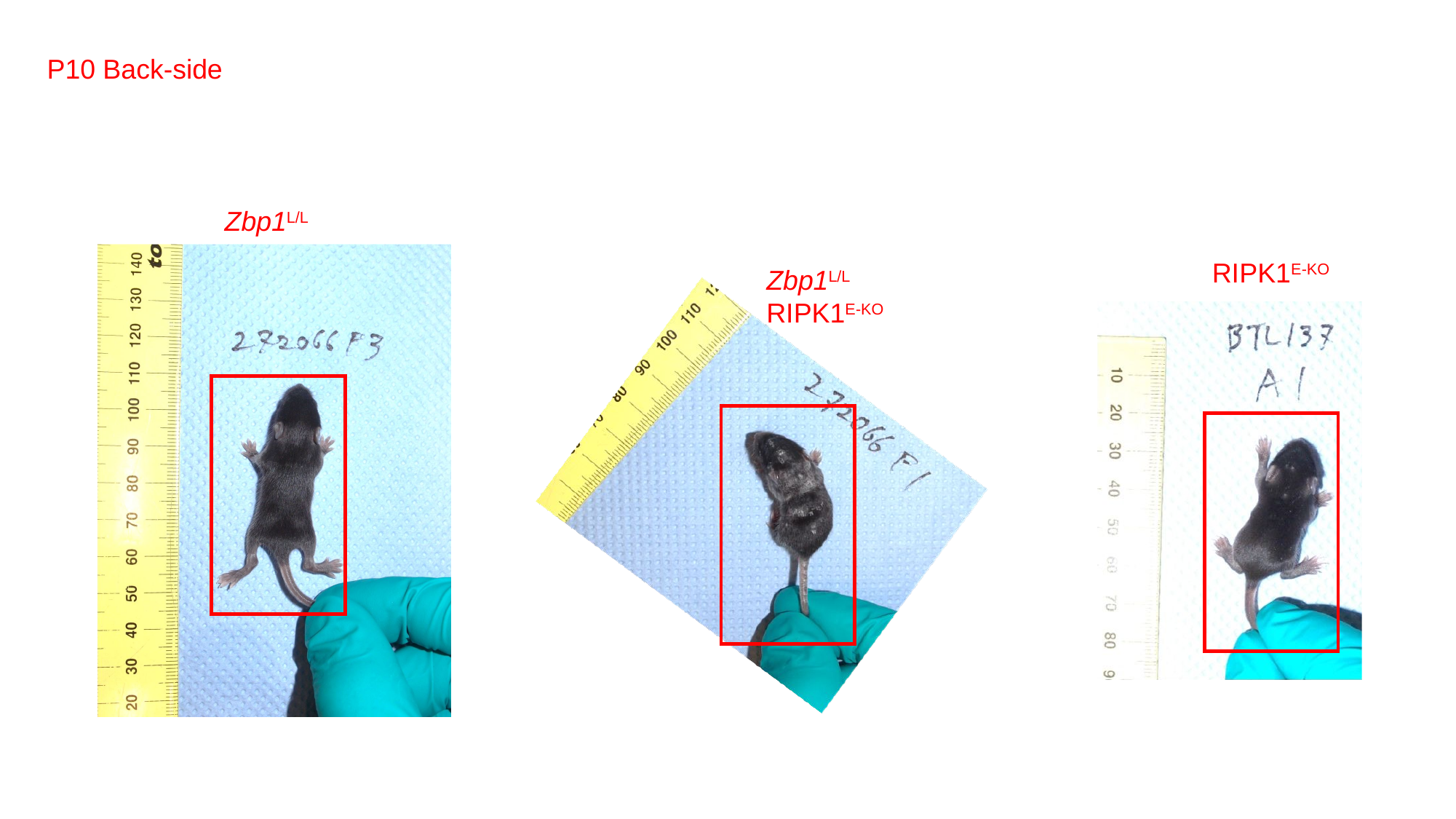

P10 Back-side
Zbp1L/L
RIPK1E-KO
Zbp1L/L
RIPK1E-KO

## Slide 2
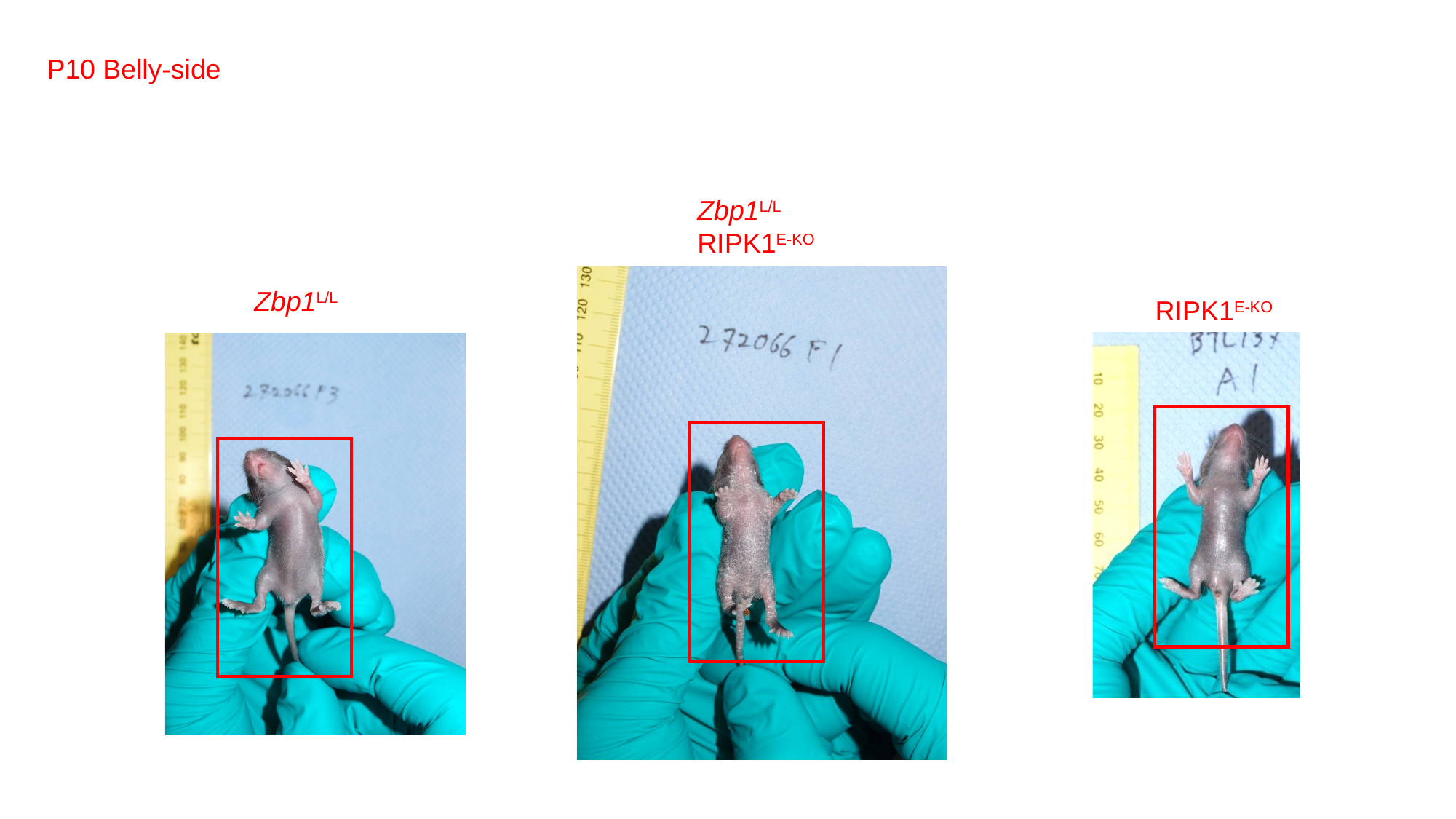

P10 Belly-side
Zbp1L/L
RIPK1E-KO
Zbp1L/L
RIPK1E-KO

## Slide 3
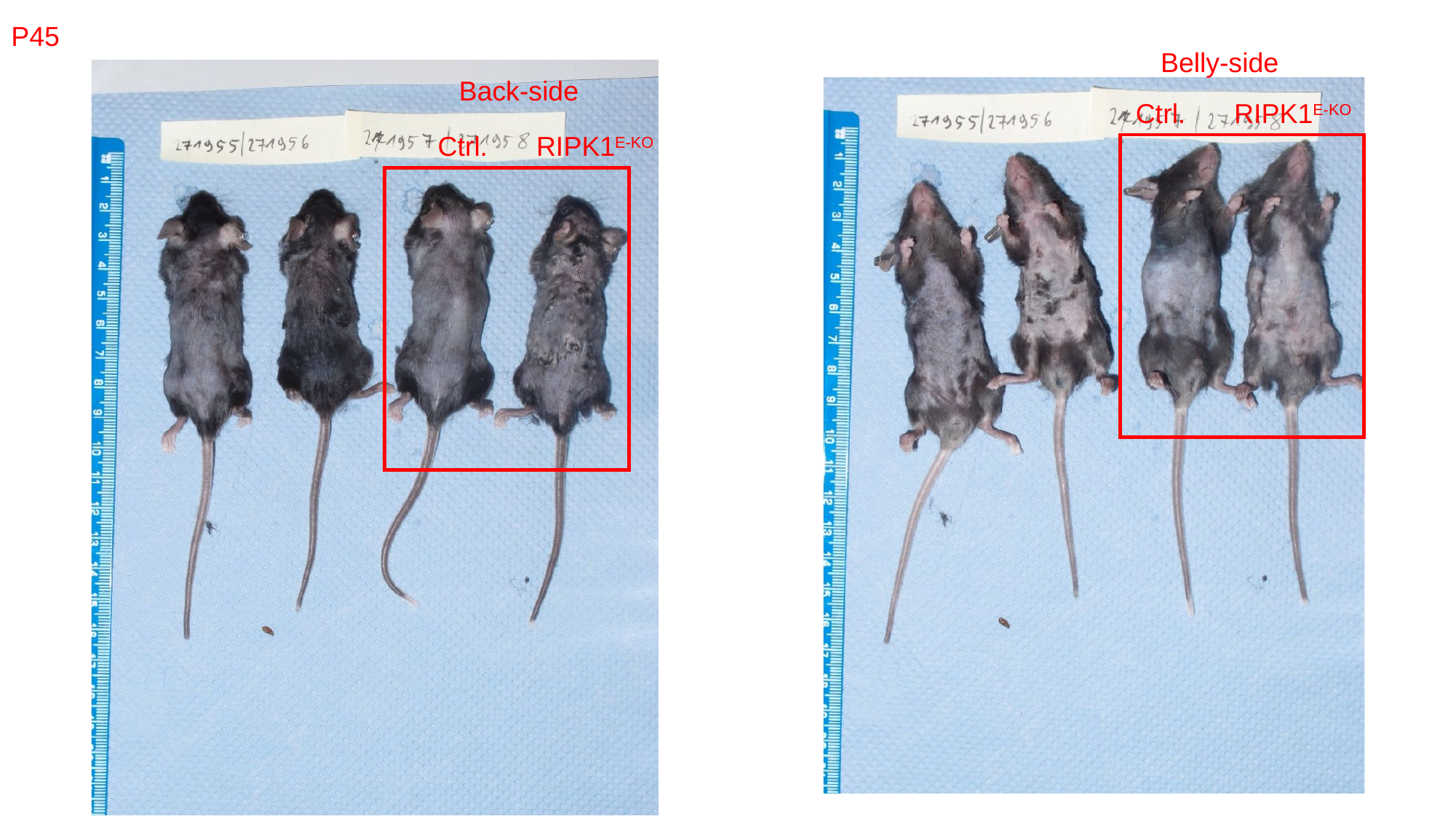

P45
Belly-side
Back-side
Ctrl.
RIPK1E-KO
Ctrl.
RIPK1E-KO
