# Supplementary material for: A shorter splicing isoform antagonizes ZBP1 to modulate cell death and inflammatory responses
Source: EMBO J. 2024 Sep 19;43(21):12. doi: 10.1038/s44318-024-00238-7 (PMC11535224; doi:10.1038/s44318-024-00238-7)
Supplement: Supplementary file 7 — Source data Fig. 5 [file 44318_2024_238_MOESM7_ESM.zip › Figure 5/5F/western Tubulin.pptx]

## Slide 1
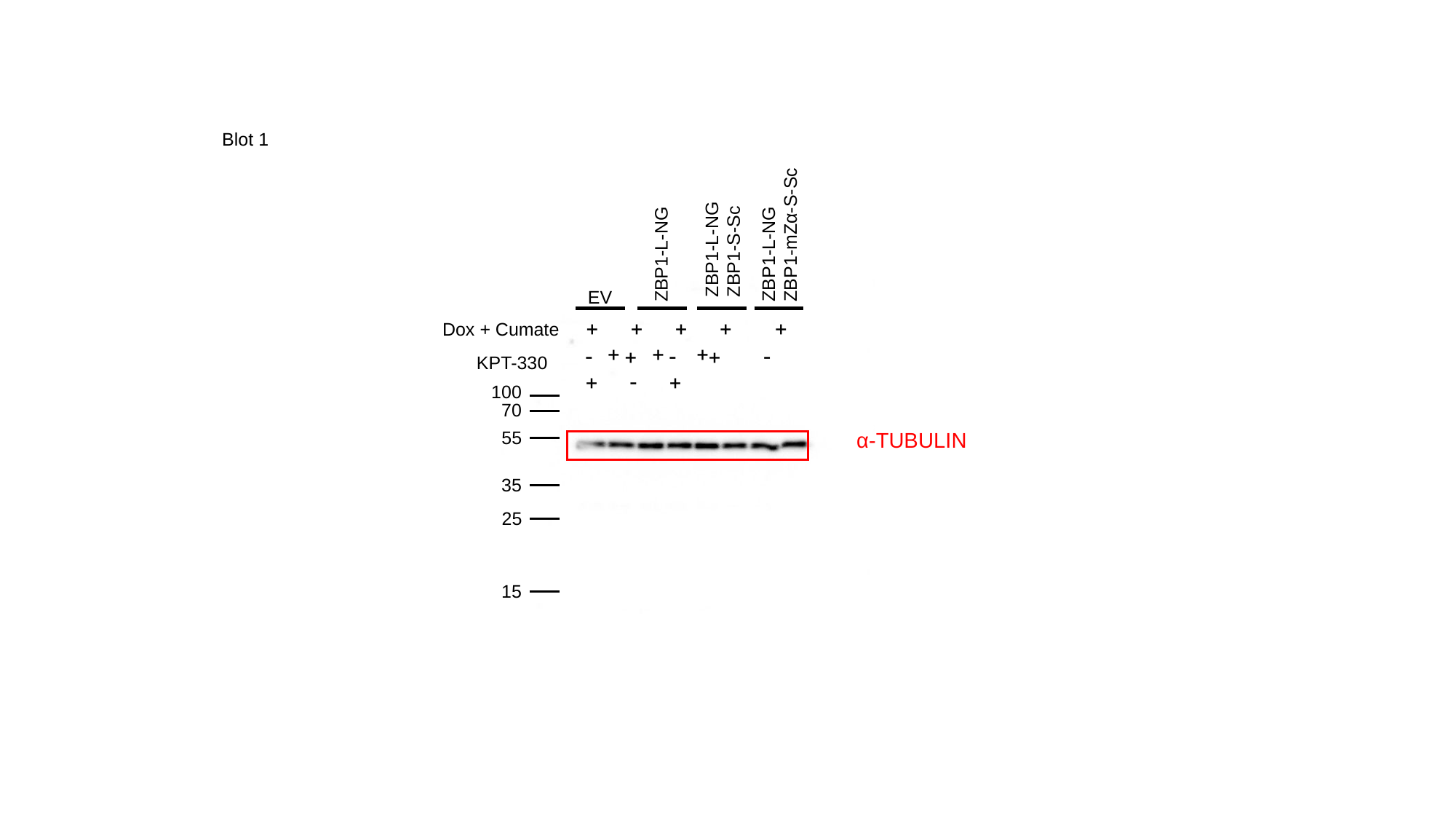

Blot 1
ZBP1-L-NG
ZBP1-mZα-S-Sc
ZBP1-L-NG
ZBP1-S-Sc
ZBP1-L-NG
EV
+ + + + + + + +
Dox + Cumate
- + - + - + - +
KPT-330
100
70
55
α-TUBULIN
35
25
15

## Slide 2
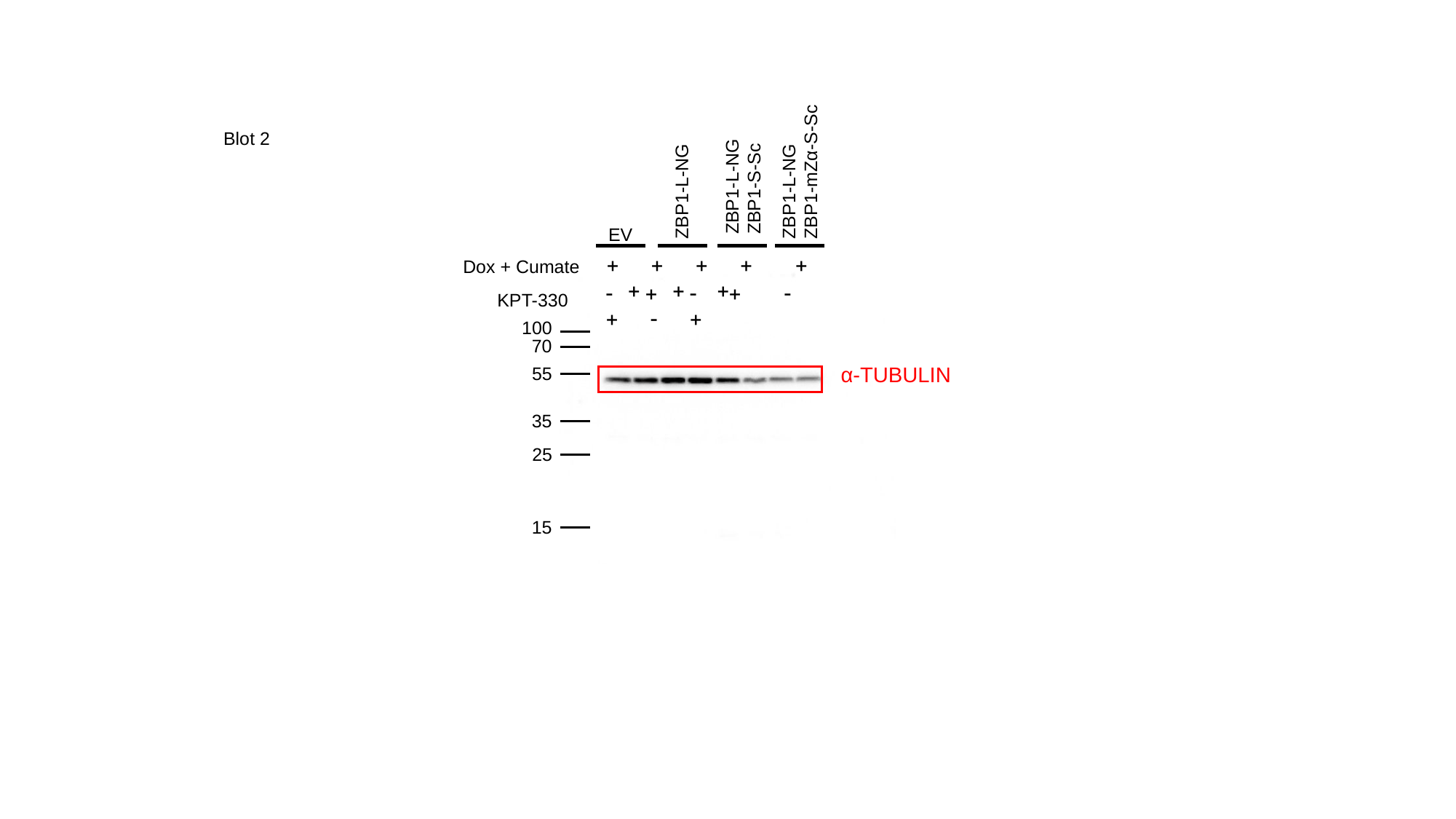

Blot 2
ZBP1-L-NG
ZBP1-mZα-S-Sc
ZBP1-L-NG
ZBP1-S-Sc
ZBP1-L-NG
EV
+ + + + + + + +
Dox + Cumate
- + - + - + - +
KPT-330
100
70
α-TUBULIN
55
35
25
15

## Slide 3
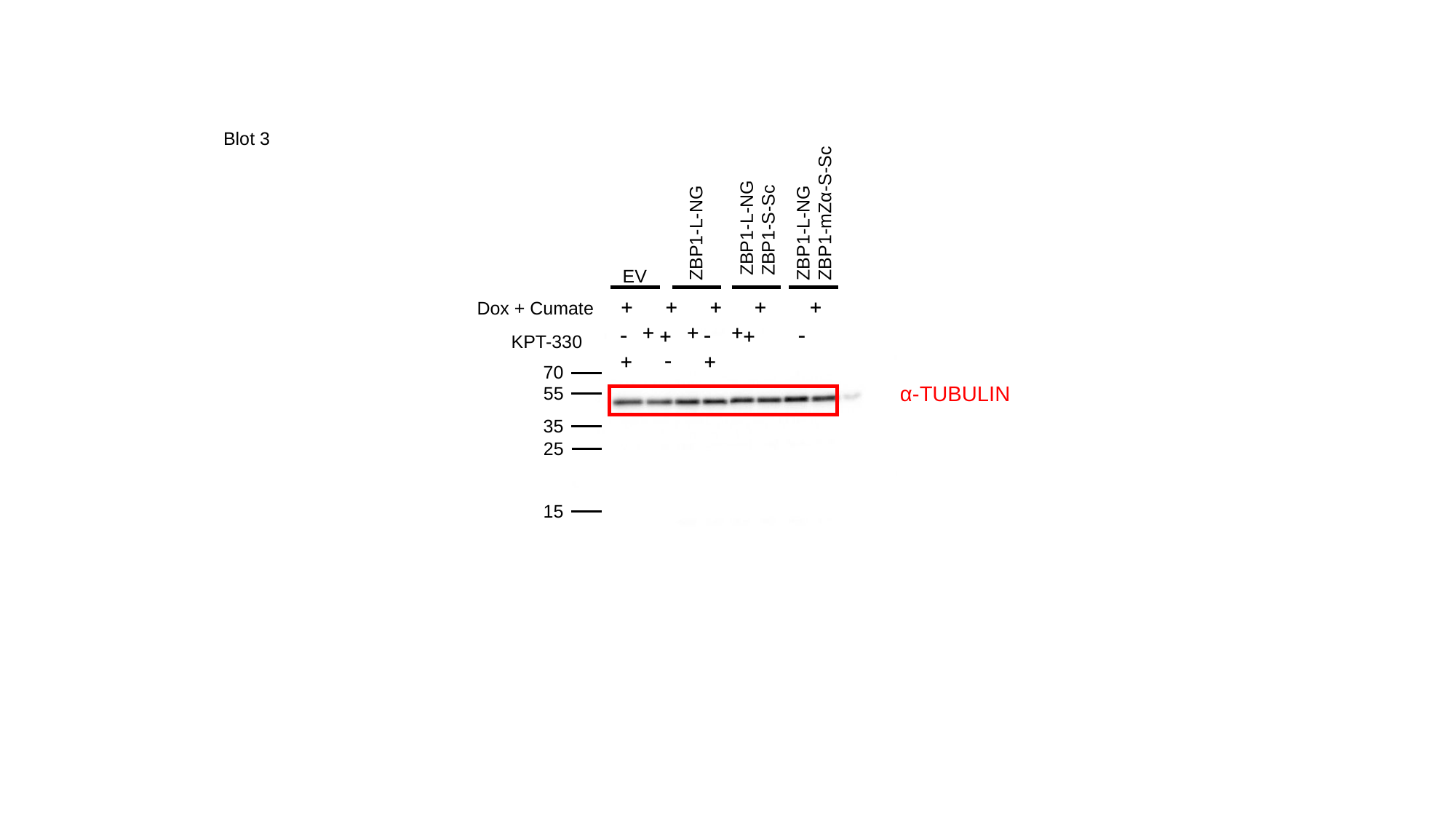

Blot 3
ZBP1-L-NG
ZBP1-mZα-S-Sc
ZBP1-L-NG
ZBP1-S-Sc
ZBP1-L-NG
EV
+ + + + + + + +
Dox + Cumate
- + - + - + - +
KPT-330
70
α-TUBULIN
55
35
25
15

## Slide 4
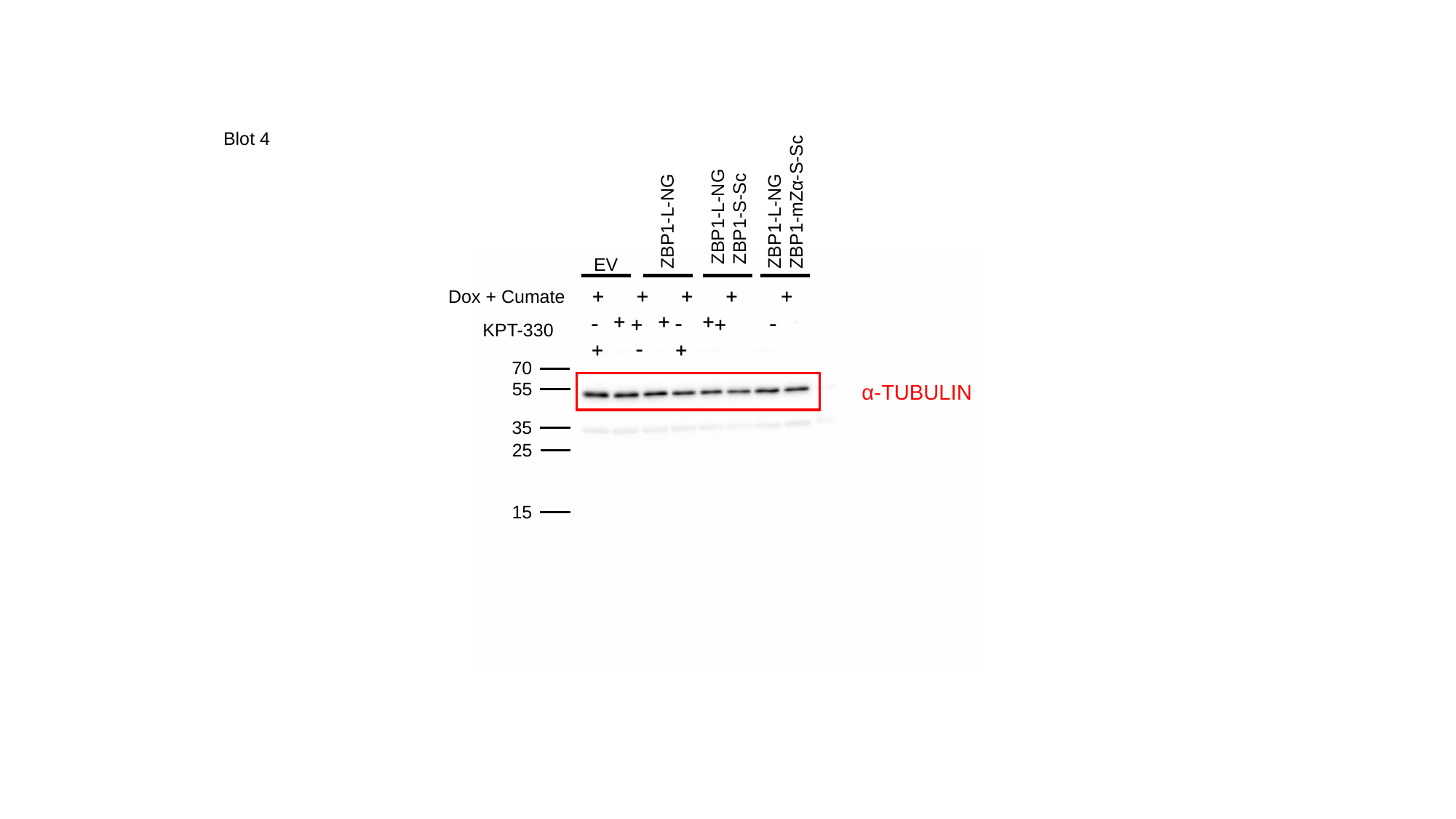

Blot 4
ZBP1-L-NG
ZBP1-mZα-S-Sc
ZBP1-L-NG
ZBP1-S-Sc
ZBP1-L-NG
EV
+ + + + + + + +
Dox + Cumate
- + - + - + - +
KPT-330
70
55
α-TUBULIN
35
25
15
