# Supplementary material for: A shorter splicing isoform antagonizes ZBP1 to modulate cell death and inflammatory responses
Source: EMBO J. 2024 Sep 19;43(21):12. doi: 10.1038/s44318-024-00238-7 (PMC11535224; doi:10.1038/s44318-024-00238-7)
Supplement: Supplementary file 7 — Source data Fig. 5 [file 44318_2024_238_MOESM7_ESM.zip › Figure 5/5D/western GAPDH.pptx]

## Slide 1
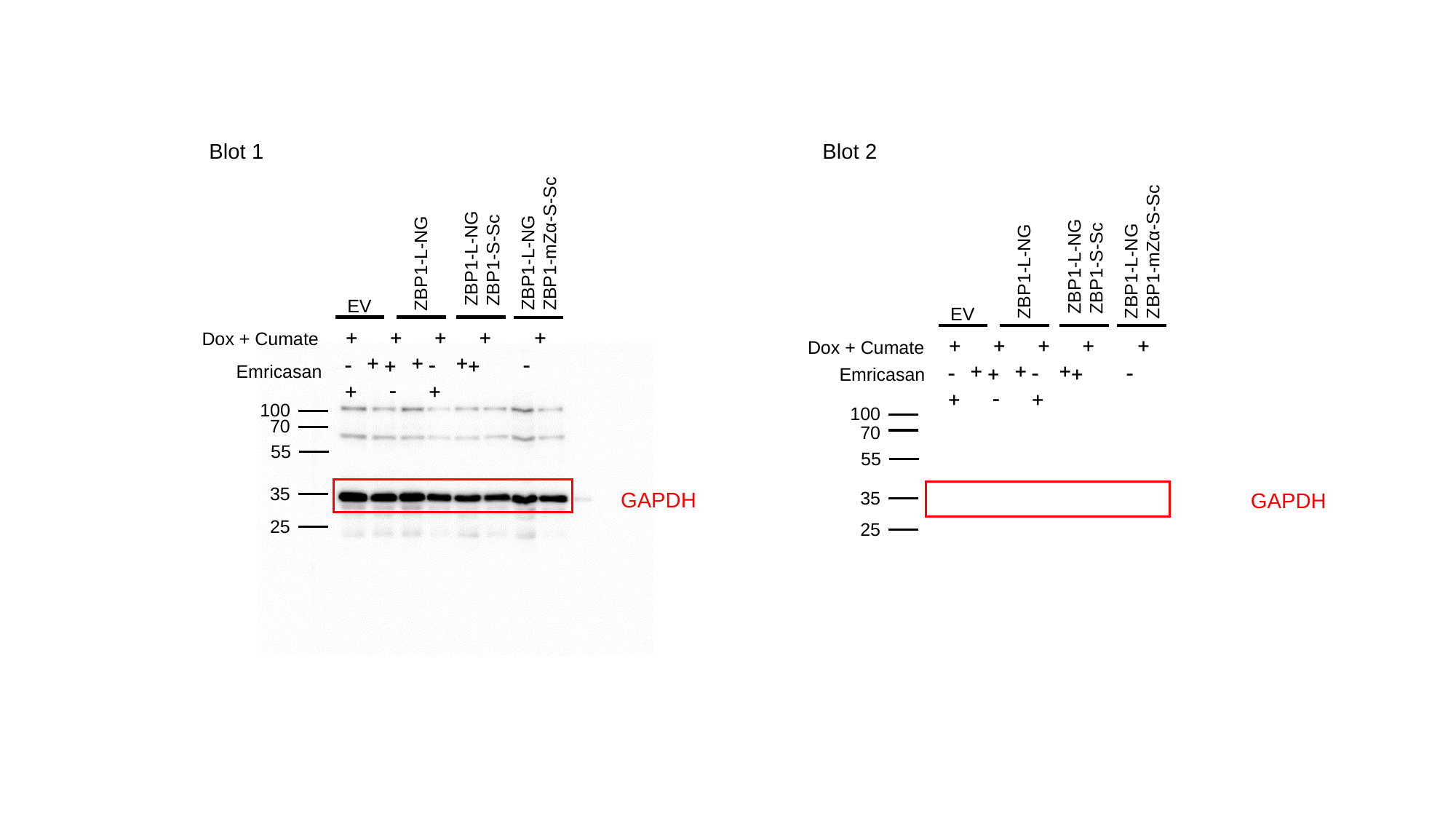

Blot 1
Blot 2
ZBP1-L-NG
ZBP1-mZα-S-Sc
ZBP1-L-NG
ZBP1-mZα-S-Sc
ZBP1-L-NG
ZBP1-S-Sc
ZBP1-L-NG
ZBP1-S-Sc
ZBP1-L-NG
ZBP1-L-NG
EV
EV
+ + + + + + + +
Dox + Cumate
+ + + + + + + +
Dox + Cumate
- + - + - + - +
Emricasan
- + - + - + - +
Emricasan
100
100
70
70
55
55
35
GAPDH
35
GAPDH
25
25
