# Supplementary material for: A shorter splicing isoform antagonizes ZBP1 to modulate cell death and inflammatory responses
Source: EMBO J. 2024 Sep 19;43(21):12. doi: 10.1038/s44318-024-00238-7 (PMC11535224; doi:10.1038/s44318-024-00238-7)
Supplement: Supplementary file 8 — Source data Fig. 6 [file 44318_2024_238_MOESM8_ESM.zip › Figure 6/6C/Neon Green IP/western RIPK3.pptx]

## Slide 1
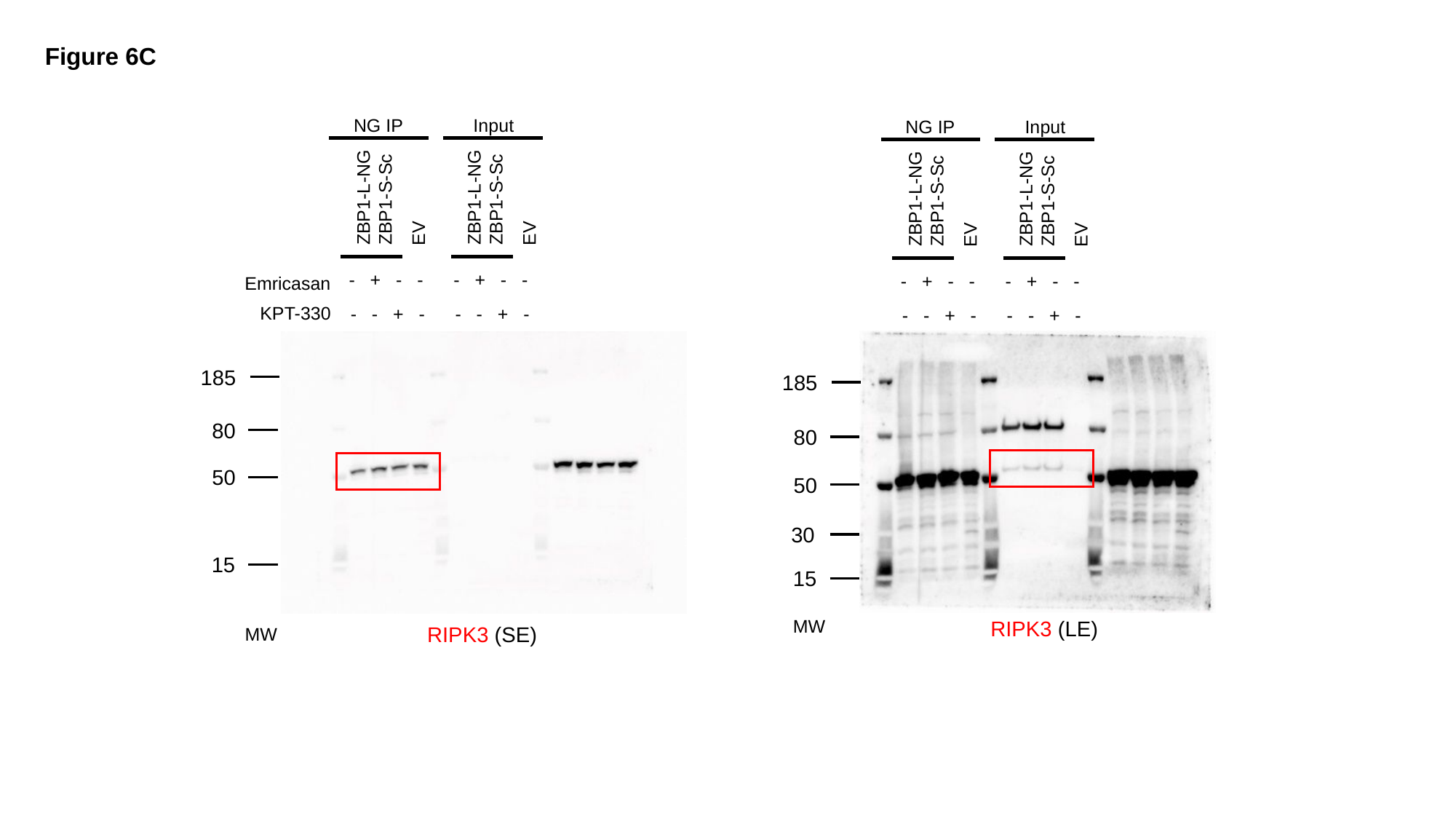

Figure 6C
NG IP
Input
NG IP
Input
ZBP1-L-NG
ZBP1-S-Sc
ZBP1-L-NG
ZBP1-S-Sc
ZBP1-L-NG
ZBP1-S-Sc
ZBP1-L-NG
ZBP1-S-Sc
EV
EV
EV
EV
- + - - - + - -
- + - - - + - -
Emricasan
KPT-330
- - + - - - + -
- - + - - - + -
185
185
80
80
50
50
30
15
15
MW
RIPK3 (LE)
RIPK3 (SE)
MW
