# Supplementary material for: A shorter splicing isoform antagonizes ZBP1 to modulate cell death and inflammatory responses
Source: EMBO J. 2024 Sep 19;43(21):12. doi: 10.1038/s44318-024-00238-7 (PMC11535224; doi:10.1038/s44318-024-00238-7)
Supplement: Supplementary file 9 — EV Figure Source Data [file 44318_2024_238_MOESM9_ESM.zip › Figure EV3/EV3F/Western CASP3.pptx]

## Slide 1
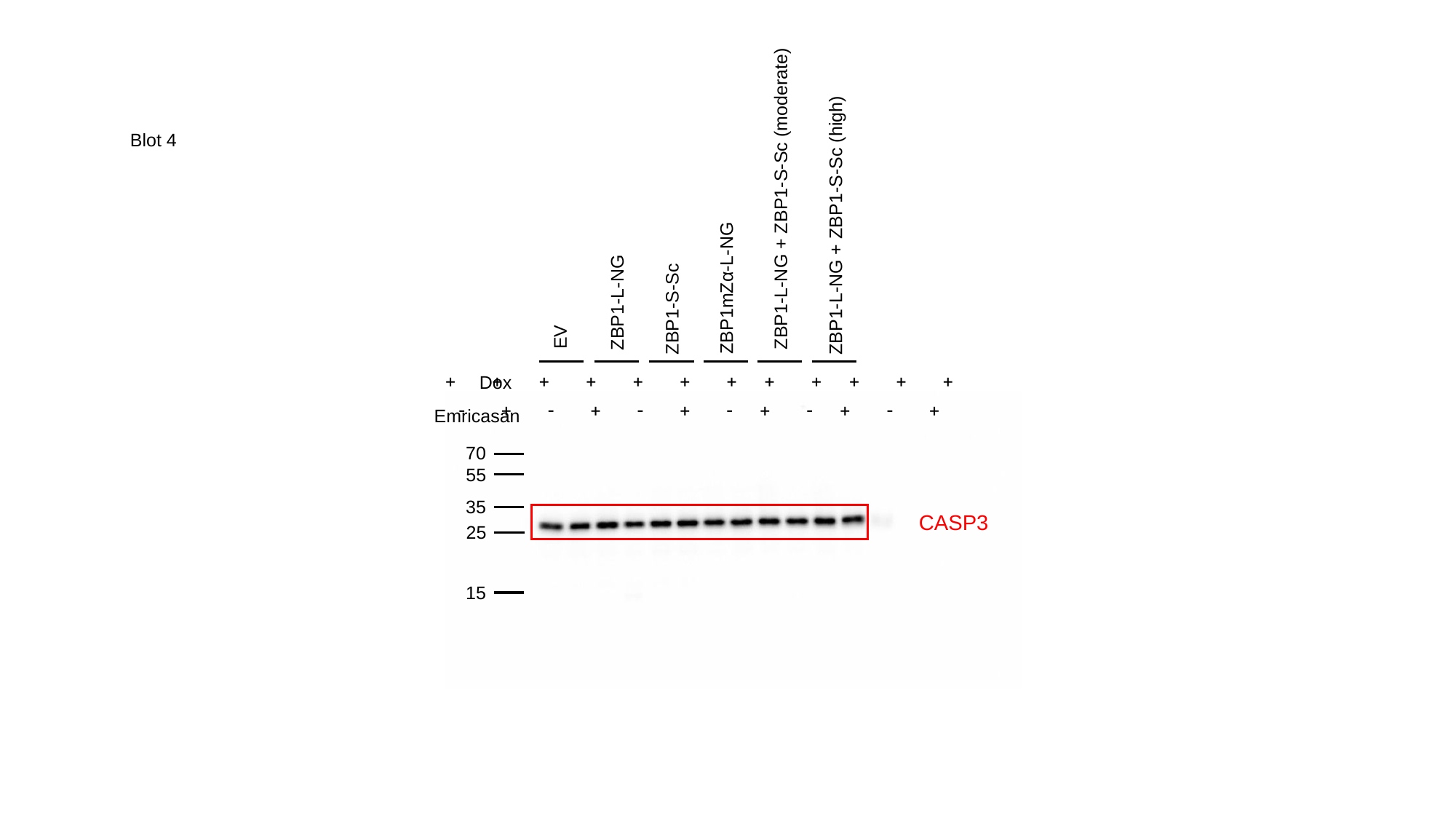

Blot 4
ZBP1-L-NG + ZBP1-S-Sc (moderate)
ZBP1-L-NG + ZBP1-S-Sc (high)
ZBP1mZα-L-NG
ZBP1-L-NG
ZBP1-S-Sc
EV
+ + + + + + + + + + + +
Dox
- + - + - + - + - + - +
Emricasan
70
55
35
CASP3
25
15
