# Supplementary material for: A shorter splicing isoform antagonizes ZBP1 to modulate cell death and inflammatory responses
Source: EMBO J. 2024 Sep 19;43(21):12. doi: 10.1038/s44318-024-00238-7 (PMC11535224; doi:10.1038/s44318-024-00238-7)
Supplement: Supplementary file 10 — Expanded View Figures [file 44318_2024_238_MOESM10_ESM.pdf]

Expanded View Figures

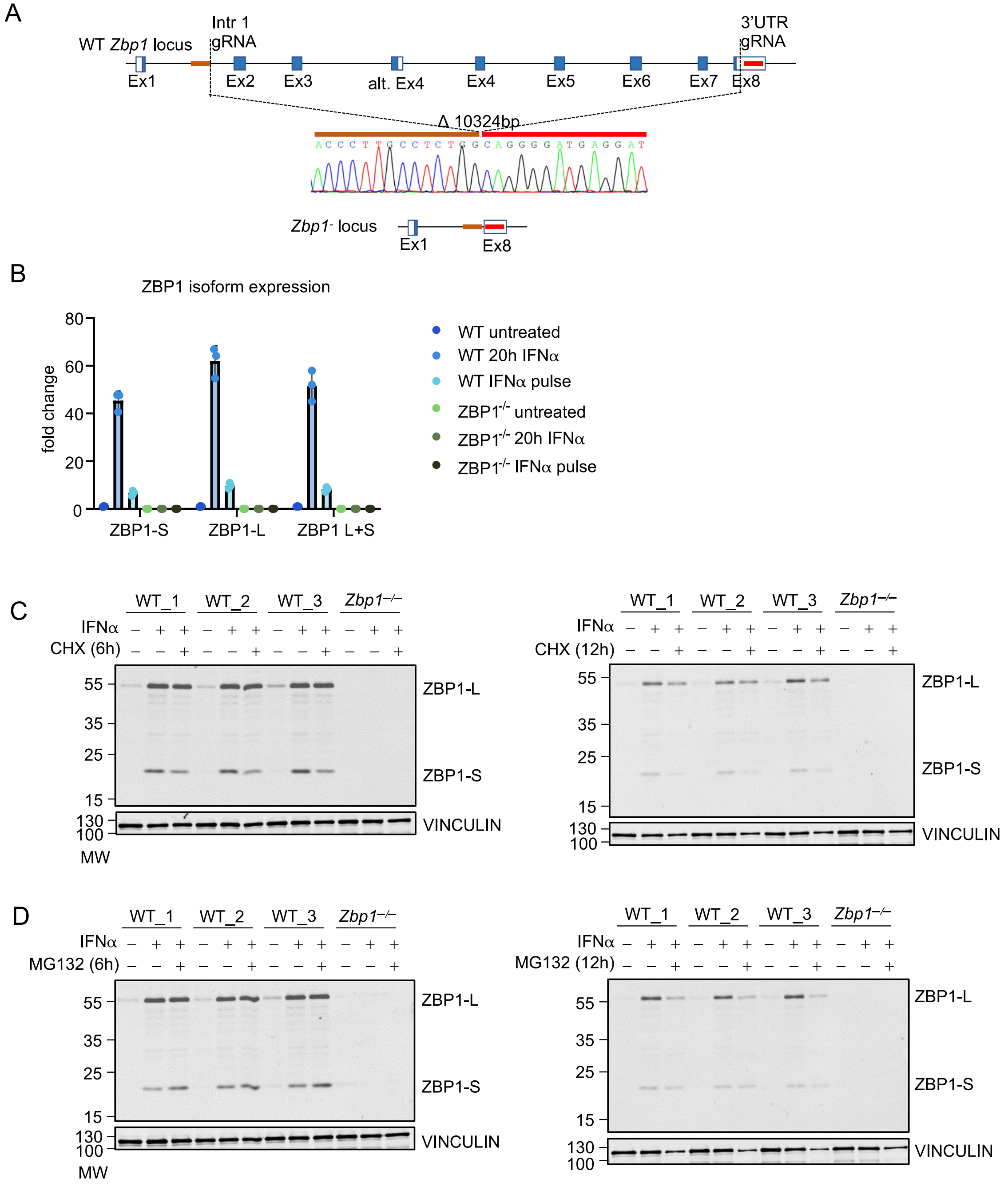

Figure EV1. Assessment of ZBP1-S and ZBP1-L mRNA and protein expression.

(**A**) Schematic depicting the generation of novel ZBP1-deficient mice (*Zbp1*$^{-/-}$) using CRISPR-Cas9-mediated gene targeting in C57BL/6N zygotes, as indicated. To generate *Zbp1*$^{-/-}$ mice, most of the *Zbp1* gene was removed by cutting once upstream of exon 2 and once within the 3′UTR in exon 8, leading to loss of the intervening region via nonhomologous end joining. The deletion event was confirmed by Sanger sequencing as indicated. (**B**) qPCR on RNA extracted from primary lung fibroblasts from three WT and three ZBP1$^{-/-}$ mice. Cells were either untreated, treated for 20 h with IFNα, or pulsed with IFNα (20 h IFNα treatment followed by washing and 24 h without IFNα), and primers were used to amplify only the short (ZBP1 Short), only the long (ZBP1 Long) or both (ZBP1 L + S) *Zbp1* isoforms. Data were presented as fold change relative to untreated WT samples. Dots represent individual mice. Mean ± SD is shown. (**C**, **D**). Immunoblot analysis with total lysates of lung fibroblasts from three WT mice and one *Zbp1*$^{-/-}$ mouse treated with IFNα (18 h pretreatment) and indicated chemicals (CHX, 10 μg/ml (**C**) or MG132, 5 μM (**D**)) for 6 or 12 h. Data information: Data were representative of 2 (**B**) independent experiments. Source data are available online for this figure.

© The Author(s)

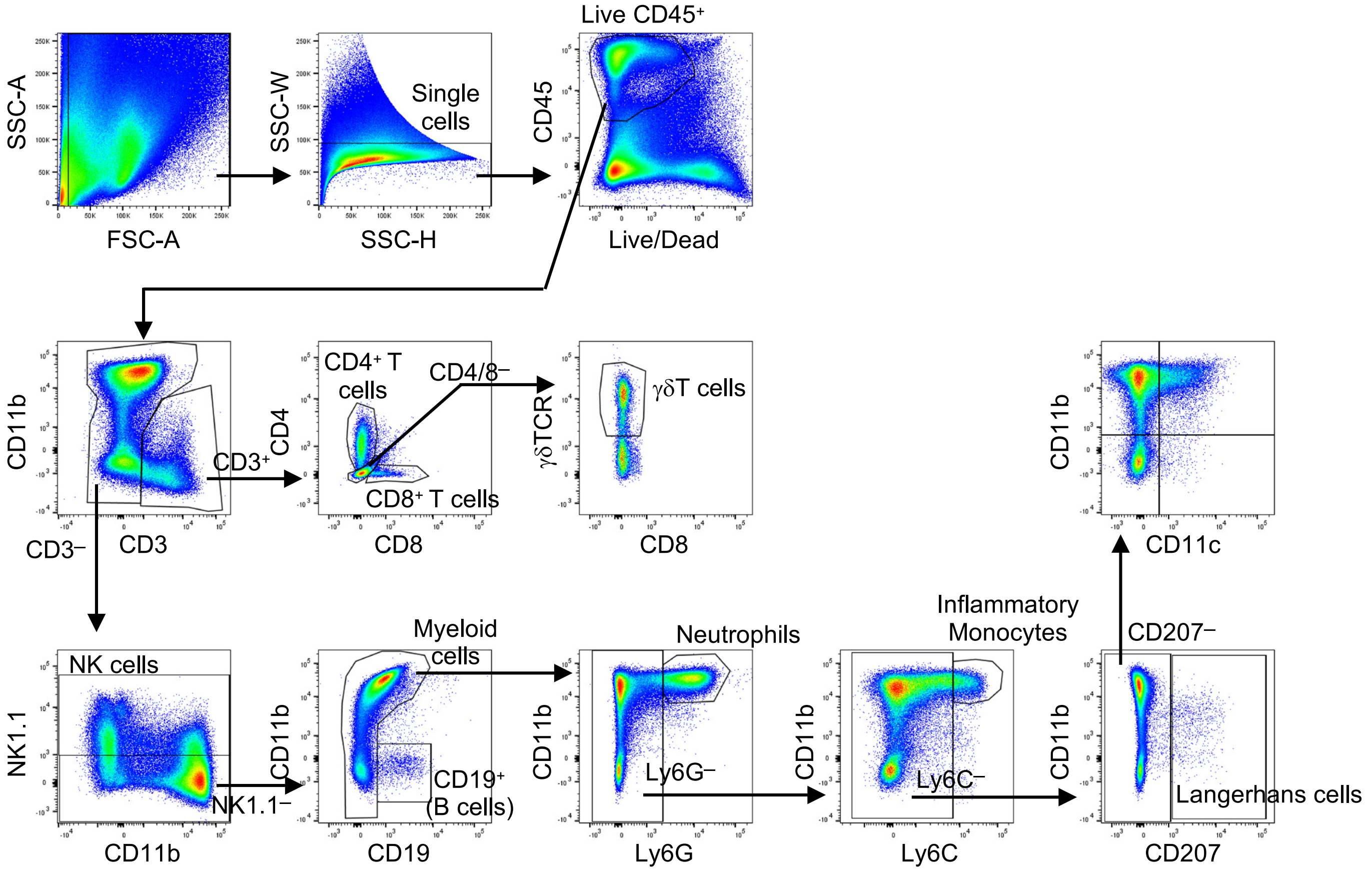

Figure EV2. Gating strategy for FACS analysis of immune cells in skin tissues.

Gating strategy for analysing immune cells in digested skin with flow cytometry. The dot plots from RIPK1$^{E\text{-}KO}$ *Zbp1*$^{L/L}$ mouse is used as representative. The viability of cells is determined by the staining with LIVE/DEAD^{TM} Fixable Near-IR Dead Cell Stain Kit (Live/Dead). Dead cells are presented as Live/Dead^{+}, and live cells are presented as Live/Dead^{-}. FSC forward scatter, SSC side scatter.

 © The Author(s)

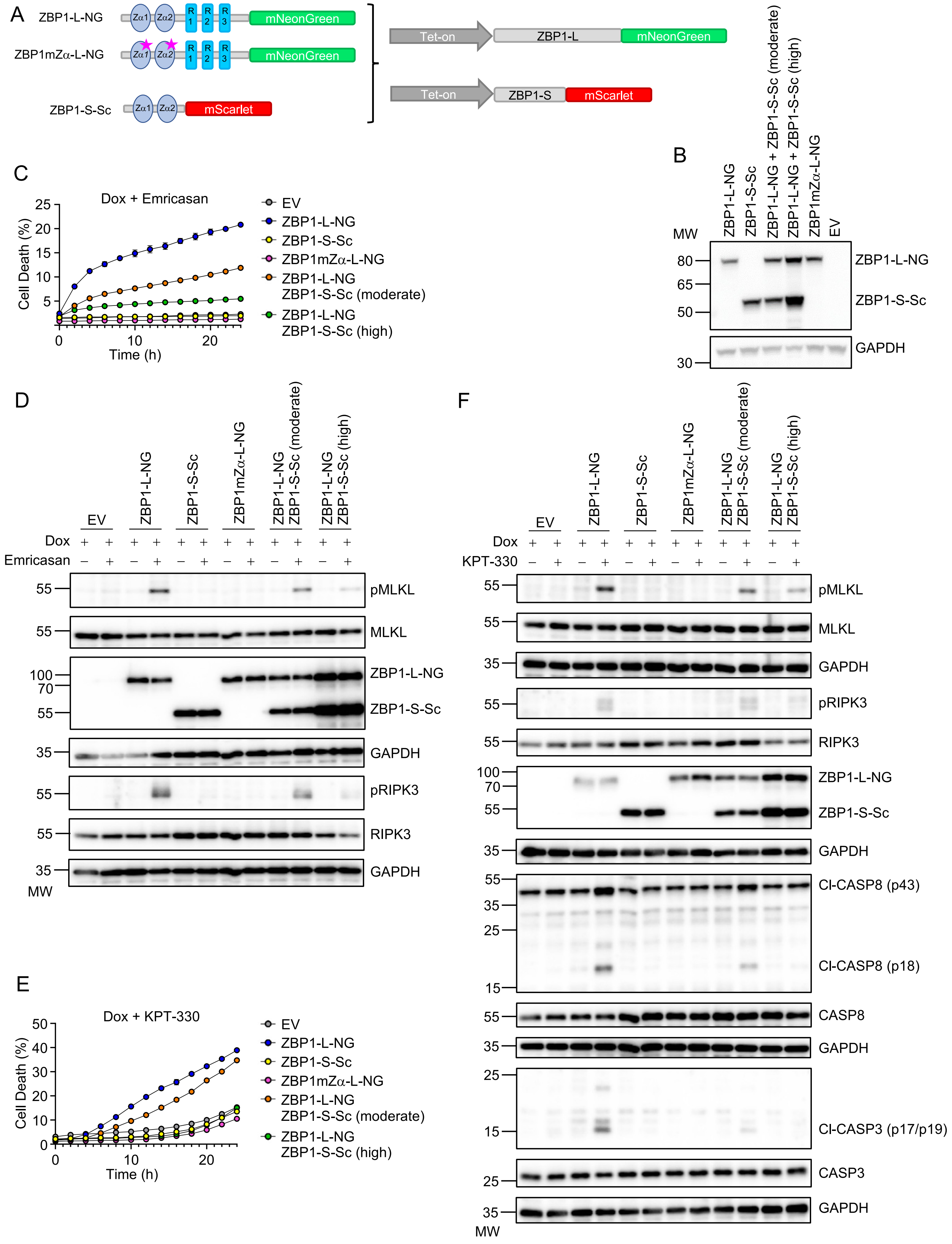
A
ZBP1-L-NG
ZBP1mZα-L-NG
ZBP1-S-Sc
mNeonGreen
mScarlet
Tet-on
ZBP1-L
ZBP1-S
B
ZBP1-L-NG
ZBP1-S-Sc
ZBP1-L-NG + ZBP1-S-Sc (moderate)
ZBP1-L-NG + ZBP1-S-Sc (high)
ZBP1mZα-L-NG
EV
MW
GAPDH
C
Dox + Emricasan
Cell Death (%)
Time (h)
ZBP1-L-NG ZBP1-S-Sc (moderate)
ZBP1-L-NG ZBP1-S-Sc (high)
D
Dox
Emricasan
pMLKL
MLKL
pRIPK3
RIPK3
E
Dox + KPT-330
F
KPT-330
Cl-CASP8 (p43)
Cl-CASP8 (p18)
CASP8
Cl-CASP3 (p17/p19)
CASP3

© The Author(s)

◀ **Figure EV3. ZBP1-S expression levels correlate with its capacity to suppress ZBP1-L-mediated cell death.**

(**A**) Schematic depicting the fusion proteins of ZBP1-L-NG, ZBP1mZα-L-NG, or ZBP1-S-Sc and the Dox-inducible systems. (**B**) Immunoblot analysis of total lysates from immortalized MEFs expressing doxycycline (dox)-inducible ZBP1-L-NG, ZBP1-S-Sc, ZBP1-L-NG with moderate or high expression levels of ZBP1-S-Sc, ZBP1mZα-L-NG, and empty vector (EV) stimulated with doxycycline for 24 h. (**C**, **D**) Cell death measured by DRAQ7 uptake (**C**) and immunoblot analysis of total lysates (**D**) in iMEFs expressing the indicated fusion proteins stimulated with doxycycline (1 μg/ml) (24 h pretreatment) and Emricasan (5 μM). (**E**, **F**) Cell death measured by DRAQ7 uptake (**E**) and immunoblot analysis of total lysates (**F**) in iMEFs expressing the indicated fusion proteins stimulated with doxycycline (1 μg/ml) (24 h pretreatment) and KPT-330 (10 μM). Cell death graphs show the percentage of cell death normalized to the total cell number obtained by 0.1% Triton X-100-induced cell lysis. Each value is presented as mean ± SEM from triplicate wells for each cell population ($n = 3$). For immunoblot analyses, α-TUBULIN (**D**) and GAPDH (**F**) were used as loading controls. Data information: Data were representative of 1 (**B**), 3 (**C**–**E**), 4 (**F**) independent experiments. Source data are available online for this figure.

 © The Author(s)
